# Regulation of early endosomes across eukaryotes: Evolution and functional homology of Vps9 proteins

**DOI:** 10.1111/tra.12570

**Published:** 2018-04-25

**Authors:** Emily K. Herman, Moazzam Ali, Mark C. Field, Joel B. Dacks

**Affiliations:** ^1^ Department of Cell Biology, Faculty of Medicine and Dentistry University of Alberta Edmonton Canada; ^2^ School of Life Sciences University of Dundee Dundee UK

**Keywords:** endosomes, LECA, membrane‐trafficking, phylogeny, Rab, *Trypanosoma*

## Abstract

Endocytosis is a crucial process in eukaryotic cells. The GTPases Rab 5, 21 and 22 that mediate endocytosis are ancient eukaryotic features and all available evidence suggests retained conserved function. In animals and fungi, these GTPases are regulated in part by proteins possessing Vps9 domains. However, the diversity, evolution and functions of Vps9 proteins beyond animals or fungi are poorly explored. Here we report a comprehensive analysis of the Vps9 family of GTPase regulators, combining molecular evolutionary data with functional characterization in the non‐opisthokont model organism *Trypanosoma brucei.* At least 3 subfamilies, Alsin, Varp and Rabex5 + GAPVD1, are found across eukaryotes, suggesting that all are ancient features of regulation of endocytic Rab protein function. There are examples of lineage‐specific Vps9 subfamily member expansions and novel domain combinations, suggesting diversity in precise regulatory mechanisms between individual lineages. Characterization of the Rabex5 + GAPVD1 and Alsin orthologues in T. brucei demonstrates that both proteins are involved in endocytosis, and that simultaneous knockdown prevents membrane recruitment of Rab5 and Rab21, indicating conservation of function. These data demonstrate that, for the Vps9‐domain family at least, modulation of Rab function is mediated by evolutionarily conserved protein‐protein interactions.

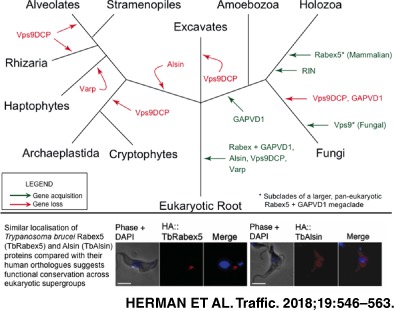

## 1 INTRODUCTION

During endocytosis, cells take up and deliver extracellular and surface material to early endosomes. These subsequently undergo a maturation process, together with sorting of cargo, prior to either lysosomal degradation, recycling to the surface, or transport elsewhere within the cell. The Rab5 GTPase subfamily and its close relatives Rab21 and Rab22 are key regulators of multiple endocytic steps and processes. They participate in fusion of plasma membrane‐derived clathrin‐coated vesicles with endosomes, endosomal maturation and also homotypic endosome fusion. Furthermore, Rab proteins define membrane subdomains within endosomal structures, which facilitate many of these processes. Rab5 also interacts with vesicle cargo, the cytoskeleton, and signal transduction factors to coordinate vesicular movement and organellar maturation within the early endocytic system.

Comparative genomic and phylogenetic analyses have demonstrated the presence of Rab5, Rab21 and Rab22 subfamily members across the breadth of eukaryotes^1^, ^2^ indicating that these Rabs were likely present in the Last Eukaryotic Common Ancestor (LECA). This is confirmed by the characterization of Rab5 orthologues in diverse eukaryotes representing each of the 5 eukaryotic supergroups,^3^ and highlights the importance of this particular Rab protein and its functions to eukaryotic cells in general.

The GTP cycle of Rabs is regulated by GTPase‐activating proteins (GAPs) and Guanine nucleotide Exchange Factors (GEFs), which accelerate the hydrolysis of GTP and exchange of GDP for GTP, respectively. While the evolution of Rab GAP protein families has been examined,^4^ the distribution and evolution of Rab GEFs is less well‐understood. Numerous Rab5 subfamily effectors have been identified in animal and fungal organisms, suggesting a complex network of interacting proteins.^5^ The known Rab5 GEFs are characterized by a Vps9 domain. The core catalytic region of human Vps9 proteins is comprised of a bundle of 4 alpha‐helices N‐terminal to the six‐helix Vps9 domain, with a final C‐terminal helix.^6^ Rab5 binds in a shallow hydrophobic groove between the V4 and V6 helices of the Vps9 domain in both human^6^, ^7^ and plant^8^ structural analyses. As proteins containing Vps9 domains have been characterized or identified from organisms in different eukaryotic supergroups, some form of Vps9 protein was likely present in the LECA.^9^, ^10^, ^11^, ^12^

The LECA possessed a sophisticated membrane trafficking system (MTS), at least as complex in terms of the number of components, and by inference pathways, as that of most extant eukaryotes,^13^ with lineage‐specific gene family expansions of MTS machinery generating organellar and trafficking pathway diversity. While there is a canonical pan‐eukaryotic complement of Rabs and Rab effectors, there have also been numerous lineage‐specific expansions and contractions, likely as part of the process of adaptation to specific environments.^1^, ^14^ In line with this, though Rab5 is ancient and found across eukaryotes, it has undergone independent gene duplication events in many lineages, suggesting ongoing specialization of the early endocytic system.^14^, ^15^ Like Rab5, multiple Vps9 domain‐containing GEFs are present in mammals: Vps9/Rabex5, VPS9‐ankyrin‐repeat protein (Varp), Ras and Rab Interactors (RIN) 1‐3, Alsin, GAPVD1 and Vps9 domain‐containing protein 1 (Vps9DCP1).^16^ Each of these proteins is involved in interactions with Rab5, or its close relatives, and plays a role in endocytosis in animal or yeast cells. However, the evolution of these families remains unexplored, and hence the level of conservation in Rab5 regulation and coordination in diverse eukaryotes remains to be examined.

We used comparative genomics to identify Vps9 domain proteins across eukaryotes and classified them using a Scrollsaw‐style phylogenetics approach.^1^ Then, in order to determine how Vps9 family proteins may function in organisms outside of the Opisthokonta (animals and fungi), we performed localization and knockdown experiments of the 2 Vps9 paralogues in the excavate parasite *Trypanosoma brucei*, a divergent organism with a comparatively well‐characterized endosomal system.

## 2 RESULTS

### 2.1 Vps9 domain‐containing proteins are pan‐eukaryotic

**Figure 1:**
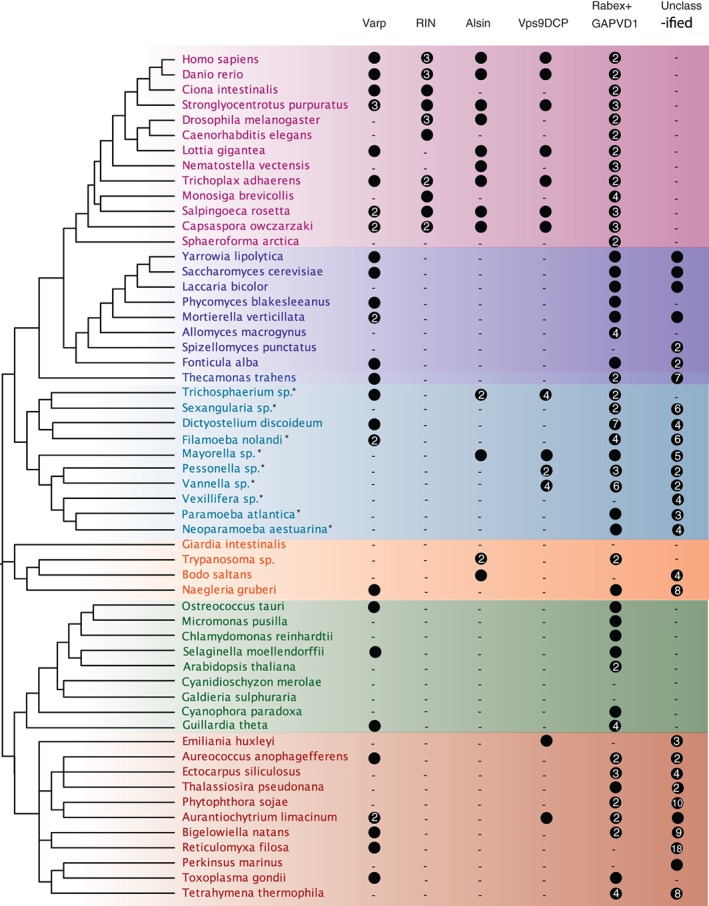
Phylogenetic classification of Vps9 family proteins across the eukaryotes. A general phylogeny is shown on the left, with an agnostic root (based on References 17, 18, 19. Supergroups and large clades are coloured as follows: pink, Holozoa; purple, Fungi; dark blue, Apusozoa (outgroup of Obazoa); light blue, Amoebozoa; orange, Excavata; green, Archaeplastida and Cryptophyta; red, SAR and Haptophyta. A filled circle indicates the presence of a protein with numbers indicating the number of paralogues, as classified by phylogenetics. A dash indicates failure to identify a sequence in that clade. It should be noted that many of the amoebozoan datasets are transcriptomes, denoted by an asterisk; therefore no statements of absence can be made for these taxa

We interrogated predicted proteomes from 48 genomes and 9 amoebozoan transcriptomes, because the Amoebozoa are represented by fewer fully sequenced genomes than the remaining supergroups. We used functionally characterized human Vps9 domain‐containing proteins as BLASTp queries, followed by a more stringent search, where the Pfam Vps9 domain hidden Markov model was used in HMMer searches. Using these methods, 311 Vps9 domain‐containing proteins were identified (Figure 1, Table S1). Most genomes encoded more than 1 Vps9 domain‐containing protein, and only a few apparently lack Vps9‐domain proteins, specifically the excavate *Giardia intestinalis,* and the red algae *Cyanidioschyzon merolae* and *Galdieria sulphuraria.*

Initial attempts to classify these sequences using phylogenetics generated trees with no resolution, owing to the relatively short Vps9 domain (104 amino acids) and high rate of lineage‐specific sequence evolution. We therefore took the following approach. A well‐curated alignment of metazoan Vps9 domain sequences was created, which generated Bayesian and maximum‐likelihood phylogenies with strong backbone support for individual clades. These clades largely contain sequences with identical domain organization to their human orthologues, and which retrieve the human orthologue as the top hit in BLASTp searches into *Homo sapiens.* Using this backbone alignment (excluding only 2 long‐branching sequences), we iteratively aligned individual sequences from each supergroup or subclade therein to generate a series of trees classifying all recovered Vps9 domain sequences. Clade‐specific phylogenies of all Vps9 domain‐containing sequences are found in Figure S1A‐R, while the original metazoan backbone tree is found in Figure S1S. While many Vps9 domain‐containing sequences across eukaryotes could be classified as orthologous to those characterized in human and yeast, others could not be classified in this way (Figure 1). In some cases, these sequences are clade‐specific expansions that have not yet been functionally characterized because of their absence from typical model organisms (eg, a clade of Stramenopile‐specific Vps9 domain‐containing proteins, Table S1). In other cases, the failure to classify these sequences may be because of high levels of sequence divergence, raising the possibility of neofunctionalization.

### 2.2 There were at least three primordial Vps9 family proteins in the LECA

**Figure 2:**
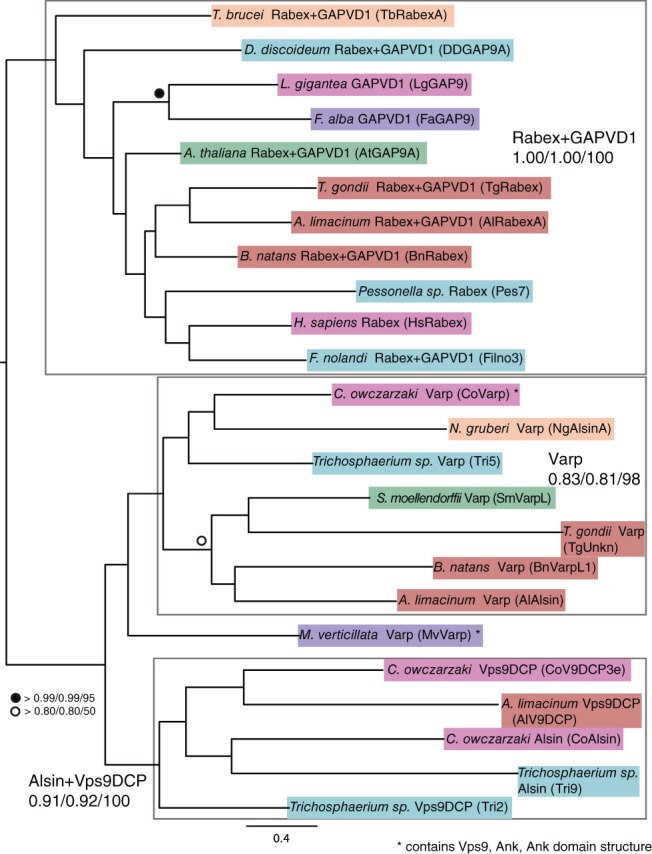
Scrollsaw tree of Vps9 family proteins. The shortest branching sequence of each supergroup or subclade for each Vps9 subfamily was included in the scrollsaw tree. The tree is shown with Phylobayes, MrBAYES and RAxML node support values (Phylobayes posterior probability/MrBAYES posterior probability/RAxML bootstrap) on the Phylobayes topology. Sequences are color‐coded as in Figure 1. Taxa are named as per their phylogenetically validated classification, with their unique identifier from Table S1 in parentheses. There are 3 obvious clades of Vps9 family proteins: Rabex5 + GAPVD1, Varp and Alsin+Vps9 domain‐containing protein, separated by grey boxes

In order to confirm that these trees reflect real evolutionary relationships between the sequences, we then used the Scrollsaw method^1^ to generate a pan‐eukaryotic phylogeny of Vps9 sequence evolution. The shortest‐branching taxon from each subclade in each MrBAYES‐generated supergroup tree was selected and carefully aligned. In cases where trees were generated for multiple subclades within a supergroup, the human RIN2 sequence was used as a standard against which branch lengths were compared, as RIN is clearly restricted to the Holozoa, and therefore the branch lengths within this clade should remain relatively stable between trees. Phylogenetic trees were generated from this alignment, showing that there are at least 3 Vps9 clades that are pan‐eukaryotic: Vps9DCP + Alsin, Rabex5 + GAPVD1 and Varp (Figure 2). The Rabex5 + GAPVD1 clade splits into clear Rabex5 and GAPVD1 sequences in the Amorphea (Amoebozoa and Opisthokonta, Figure S1A‐K), suggesting an ancient, but post‐LECA, gene duplication event prior to the split of the Amoebozoa and Opisthokonta supergroups. However, we found no evidence for such a post‐LECA duplication within Vps9DCP + Alsin megaclade. Rather, both Alsin and Vps9DCP were clearly distinct from Varp and Rabex5 + GAPVD1 and based on their pan‐eukaryotic distributions (see below) may well represent individual LECA clades, despite their being united in an unresolved clade in Figure 2.

We therefore conservatively deduce at least 3 (and possibly 4) Vps9 domain‐containing proteins in the LECA, and through the radiation of eukaryotes, this family has undergone expansion, innovation, maintenance and loss. In order to gain more detailed evolutionary insight into the Vps9 subfamilies, each was treated in turn.

### 2.3 Varp and Vps9 domain‐containing protein are pan‐eukaryotic, but with patchy distribution

Varp contains 2 sets of 4 ankyrin repeats, followed by a Vps9 domain. Varp appears to link multiple stages of endocytosis. Although it has weak GEF activity for Rab5 in vitro, it is required for activation and endosomal localization of Rab21.^20^ In addition to Rab5 interactions, Varp and the retromer coat complex coordinate to promote recycling of cell surface receptors from endosomes.^21^ It interacts with Rab32 and the closely related Rab38,^22^, ^23^, ^24^, ^25^ which are involved in lysosome‐related organelle and autophagic vacuole biogenesis.

As shown by the individual supergroup and subclade trees (Figure S1A,C‐F,J‐P), and by Scrollsaw (Figure 2), Varp is present across the diversity of eukaryotes, albeit lacking ankyrin repeats outside of Opisthokonta. Furthermore, we generated a tree containing all candidate Varp sequences and metazoan backbone sequences, and all form a well‐supported clade with the metazoan sequences (Figure S1Q). There are examples of Varp orthologues with the Vps9‐ankyrin domain structure in basal Fungi (*Fonticula alba*) and basal Holozoa (*Capsaspora owczarzaki*), so presumably a Varp protein with this domain structure was present in their common ancestor. Searches into the genome of *Thecamonas trahens*, an apusozoan organism that diverged prior to the split of animals and fungi, also identified a Varp homolog (Figure S1Q), but it appears to lack ankyrin repeats (Table S1).

Several proteins were also identified with similar domain structure to mammalian Varp; however, phylogenetics shows them to have a distinct history. In *Dictyostelium discoideum*, a protein with the domain structure ankyrin‐ankyrin‐Vps9 was identified—a reversal of the mammalian domain organization—but this sequence was clearly excluded from the Varp clade in the *Dictyostelium* subclade trees (Figure S1D, sequence DdVarpL2). Furthermore, in the stramenopiles *Ectocarpus siliculosus* and *Aureococcus anophagefferens*, 2 proteins were identified with DUF47‐Vps9‐Ankyrin‐Ankyrin (Esi0135_0039) and Vps9‐Ankyrin‐Ankyrin (70964) domain organization, respectively. Phylogenetic analysis revealed that these sequences are part of the Rabex + GAPVD1 clade rather than Varp (Figure S1P). This is a clear example of convergent evolution in the Vps9 family of proteins, where Vps9 together with ankyrin repeat domain proteins were acquired independently at least 3 times. This repeated origin suggests that the combination is functionally relevant; however, it is not clear whether these additional Vps9/ANK‐domain proteins are functionally analogous to opisthokont Varp proteins or have distinct activity.

Varp is recruited to endosomes by direct interactions with retromer subunit Vps29 through 2 CHPLCxCxxC motifs.^21^ This critical motif appears to be conserved in Holozoa, as it is present in *C. owczarzaki.* While a Varp homologue was identified in *S. cerevisiae* (both here and by Bean et al^26^), it lacks this motif. The motif is also absent from Varp‐like homologues identified in other eukaryotes indicating that the cysteine‐rich motif appears to be a Holozoa‐specific innovation.

Unlike the other Vps9 proteins, little is known functionally about Vps9DCP1 in mammals or yeast. We consistently recovered clades of Vps9 domain‐containing proteins (Vps9DCPs) in supergroup and subclade trees that appear to be orthologous to the human Vps9 domain‐containing protein (Figure S1A,E‐G,J,N,O). Orthologues have been positively identified in members of the Holozoa, Amoebozoa, Stramenopile clade, and in the haptophyte *Emiliania huxleyi.* However, they have been lost independently in many lineages, including Fungi, excavates, archaeplastids, alveolates and Rhizaria.

### 2.4 Alsin is present in Opisthokonta, Amoebozoa and Excavata, and lost in the ancestor of stramenopiles, alveolates, and Rhizaria (SAR) and Archaeplastida

Another ancient Vps9 subfamily is Alsin. Alsin has a C‐terminal Vps9 domain which promotes the dissociation of GDP from Rab5,^27^ and it has been shown to colocalize with early endosome antigen 1 (EEA1) and stimulate endosome‐endosome fusion.^28^ Mutations in the Alsin gene are associated with ALS,^29^ juvenile‐onset primary lateral sclerosis,^30^ and infantile‐onset hereditary spastic paraplegia.^31^ Through its central diffuse B‐cell lymphoma (Dbl), diffuse homology (DH) and plekstrin homology (PH) domains, which interact specifically with the Rho family protein Rac1,^28^ Alsin promotes macropinocytosis and macropinosome‐endosome fusion.^32^ In addition to the role in endosome‐endosome and macropinosome‐endosome fusion, Alsin and its interacting partner Rac1 are enriched in membrane ruffles and lamellipodia of migrating cells. Between the DH/PH and Vps9 domains are 8 membrane occupation and recognition nexus (MORN) motifs, implicated in plasma membrane binding.^33^

We identified Alsin in the Holozoa and in the amoebozoans *Trichosphaerium*, *Mayorella,* and potentially in *Sexangularia* (Figure S1F,I,J). Interestingly, Alsin appears to be lost in the Fungi (Figure S1B). One of the characteristic features of human Alsin is the presence of MORN repeats. Only a few amoebozoan Alsin homologues have both MORN repeats and a Vps9 domain, while others possess only a Vps9 domain (Table S1). In the Excavata, there are clear Alsin orthologues in the trypanosomatids and *Bodo saltans*, which retain the MORN‐Vps9 domain structure (Figure S1K). Two *N. gruberi* sequences were also identified with these domains, but their phylogenetic position could not be resolved. Alsin orthologues could not be identified in any SAR or archaeplastid taxa. This suggests that an ancestral Alsin homolog was present in the LECA but subsequently lost from SAR and plants.

### 2.5 Rabex5 and GAPVD1 evolution in Amorphea

The final ancient Vps9 subfamily is the clade of Rabex5 and GAPVD1. Originally identified in the yeast *Saccharomyces cerevisiae* as VPS9 because of its mutation resulting in impaired vacuole acidification (class D vps mutant),^34^ both yeast Vps9p and the mammalian orthologue Rabex‐5 contain domains allowing them to bind ubiquitinated cargo (coupling of ubiquitin to ER degradation (CUE) domain and A20‐like Cys_2_/Cys_2_ zinc‐finger, respectively).^35^, ^36^, ^37^, ^38^ Through recruitment of Vps21p^39^ or Rab5,^40^ Rabex5 proteins are involved in endosome maturation and multivesicular body biogenesis. GTPase activating protein and VPS9 domain‐containing protein 1 (GAPVD1), like other Vps9 proteins, interacts with Rab5, but is thought to function prior to the early endosome, as it does not colocalize with EEA1 in *Caenorhabditis elegans.* Rabex5 (or Vps9p in yeast) acts downstream, and in complex with the Rab5 effector Rabaptin‐5, it is essential for homotypic endosome fusion.^40^ In addition to Rab5, Rabex5 interacts with other Rabs in mammals, including Rab17, Rab21 and Rab22.^41^, ^42^ GAPVD1 is named as such in humans owing to its N‐terminal RasGAP domain that has specific activity for H‐RAS, and the C‐terminal Rab5‐interacting Vps9 domain.^43^ It was originally identified in *C. elegans* (and named RME‐6) by Sato et al, who demonstrated its specific interaction with Rab5, and interaction with the clathrin adaptor complex protein AP‐2α.^44^ It is localized to the cell surface in a clathrin‐dependent manner and functions in the initial stages of endocytosis.

A single clade‐containing Rabex5 + GAPVD1 sequences was recovered in our Scrollsaw analysis (Figure 2). In each subclade tree (Figure S1), we consistently identified outgroup sequences to the individual Rabex5 and GAPVD1 clades, and which often formed strongly supported Rabex5 + GAPVD1 megaclades. These Rabex5 + GAPVD1‐related sequences are found in nearly every organism with Vps9 domain‐containing proteins, and often in multiple copies, suggesting an important and potentially conserved role. The sequences grouping in this megaclade that are excluded from Rabex5 and GAPVD1 subclades typically have only a Vps9 domain, although there are some lineage‐specific exceptions (Table S1). Based on phylogenetic analysis of individual subclades, clear orthologues of human Rabex5 and GAPVD1 appear only in the Amorphea. GAPVD1 proteins are found in the amoebozoans *Trichosphaerium* sp., and *D. discoideum* (Figure S1D,J,R), and the latter contains both a RasGAP and Vps9 domain, identical to the domain organization of the human orthologue. A further analysis of Rabex5‐ and GAPVD1‐related sequences in Amorphea revealed another GAPVD1 homolog in the amoebozoan *Sexangularia* sp. (Figure S1I). There is also a GAPVD1 in the basal holomycotan organism *F. alba* (Figure S1B), but not in any other fungal genomes searched. Interestingly, the RasGAP domain has been patchily lost from holozoan GAPVD1 sequences, and the entire GAPVD1 subfamily appears to have been lost in the Fungi, after the divergence of *F. alba.* The GAPVD1 clade's origin and presence of the eponymous RasGAP domain can be placed at least as far back as the split of the Amorphea, but it may be even more ancient, as there is a single putative GAPVD1 sequence in *Naegleria gruberi*, with RasGAP and Vps9 domains, but has limited phylogenetic support (Figure S1K).

### 2.6 RIN is a holozoan innovation

However, not all Vps9 subfamilies are ancient. RIN proteins are capable of binding Ras, and play roles in cell signaling,^43^ migration and adhesion,^45^, ^46^, ^47^ thereby linking Rab5‐mediated endocytosis and cell signaling.^48^, ^49^ In addition to GEF activity for Rab5, RIN proteins are involved in Ras binding, as well as endocytosis of various signalling molecules at the cell surface. There are 3 members in humans, RIN 1‐3, which are characterized by an N‐terminal SH2 domain, a Vps9 domain and a C‐terminal Ras‐association domain.

From the limited metazoan taxa analysed, the duplication leading to several paralogues in human appears to have occurred prior to the divergence of bony fish (Figure S1A). RIN orthologues could only be reliably identified in Holozoa, with a Vps9 and RAS association domain‐containing RIN homolog in the filasterian *C. owczarzaki* (Figure S1A). However, there is low support for a potential RIN in *D. discoideum* (XP_641280.1), which groups with the metazoan RIN clade in a *D. discoideum* subclade tree (Figure S1D). A conservative assessment of RIN proteins would place the origin of RIN prior to the divergence of the Filasteria. Given that RIN proteins are involved in cell migration through their interaction with Ras, resulting in endocytosis of cell surface proteins that mediate extracellular matrix contact and cell‐cell adhesion, it is unsurprising that this subfamily of Vps9 proteins is restricted to multicellular animals and their single‐celled relatives.

### 2.7 Novel domain innovation in Vps9 proteins across eukaryotes

Just as RIN appears to be a holozoan innovation, there are several examples of Vps9 family expansions or novel domain acquisitions in various eukaryotic lineages. The most obvious example is the previously discussed zinc finger and CUE domain additions to Rabex5 family proteins in Holozoa and Fungi, respectively. In ochrophyte stramenopiles, there are Vps9 family proteins with a PDZ domain, followed by a PX domain, and a C‐terminal Vps9 domain (Table S1). PDZ and PX domains both target proteins to cell membranes, anchoring membrane proteins to cytoskeletal components and binding phosphatidylinositol 3‐phosphate phospholipids, respectively. This suggests another independent example of Vps9 family proteins potentially linking endocytosis with membrane and cytoskeleton interactions. Additionally, there are numerous examples of Vps9 proteins in individual taxa that include additional domains, such as P‐loop NTPase, RasGAP domains, CAP‐Gly domains, PH domains, zinc finger and kelch domains, although the relationship of these proteins with metazoan Vps9 sequences is often unclear. It is also possible that genome misassembly may be the source of some of the novel domain additions observed; however, this is unlikely, particularly when seen across multiple taxa.

### 2.8 Identification and annotation of Vps9 proteins in *T. brucei*

**Figure 3:**
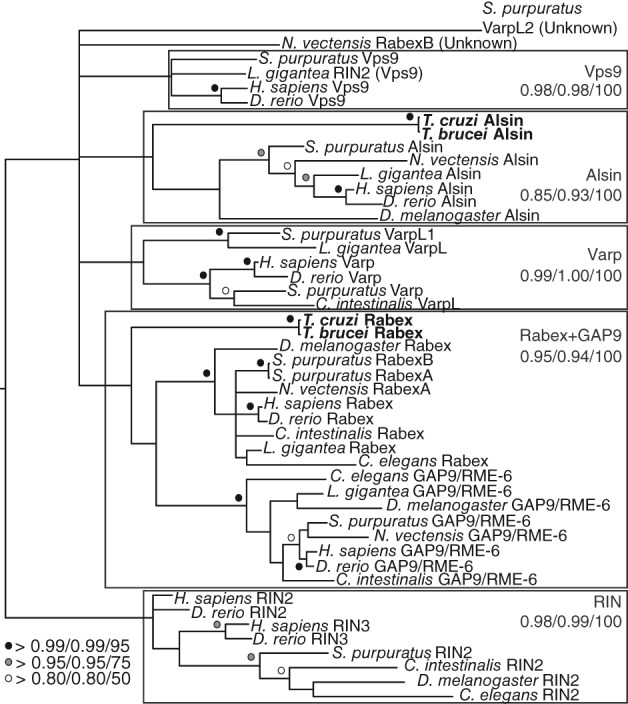
Metazoa backbone tree including T. brucei and T. cruzi Vps9 proteins. Trypanosome Vps9 sequences were classified using the metazoa backbone tree shown in Figure S1S. In cases where multiple members of a subfamily are present per taxon, the unique identifier from Table S1 is given in parentheses. The tree is shown with Phylobayes, MrBAYES and RAxML node support values (Phylobayes posterior probability/MrBAYES posterior probability/RAxML bootstrap) on the Phylobayes topology. Trypanosome sequences are bolded. TbRabex5 (TbRabexA) and TcRabexA group within the Rabex5 + GAPVD1 clade, and TbAlsin (TbRabexB) and TcRabexB group within the Alsin clade, all with significant support

Nearly all characterization of Vps9 domain proteins has been in animal and fungal models. To provide complementary functional information from a distantly related model organism, we chose to characterize 2 Vps9 family proteins in *T. brucei.* As excavate subclade phylogenies were poorly resolved (Figure S1K), in order to classify the *T. brucei* Vps9 proteins, we constructed trees with only the trypanosome and metazoa backbone sequences. We found that these sequences group with Alsin (Tb927.3.2430, designated here TbAlsin) and Rabex5 + GAPVD1 (Tb927.10.10020, designated here TbRabex5) (Figure 3). Domain analysis shows that TbAlsin contains MORN repeats, similar to Alsin‐like sequences from other non‐metazoans (Table S1). Transcription of both genes in bloodstream and insect stages was confirmed by qRT‐PCR (Figure S2A,B), confirming previously published transcriptome data.^50^, ^51^

### 2.9 Subcellular localizations of T. brucei Vps9 proteins

**Figure 4:**
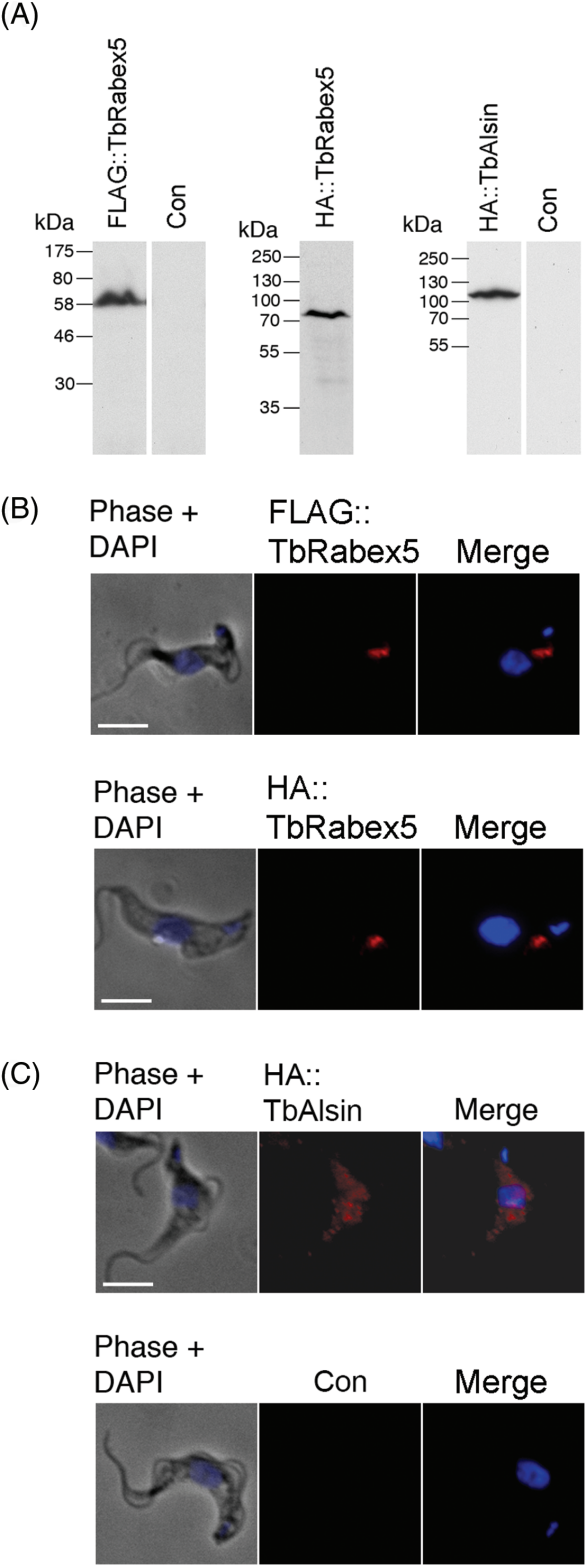
Intracellular localization of epitope tagged TbVps9 proteins in BSF cells. (A) Western blots showing expression and protein size of TbRabex5 fused to N‐terminal FLAG tag or HA tag, and TbAlsin fused to N‐terminal HA tag, along with negative controls. (B) IFA images showing ectopic expression of FLAG::TbRabex5 and HA::TbRabex5 and (C) HA::TbAlsin in bloodstream form cells. (B) Cells were labelled with monoclonal mouse anti‐FLAG and counterstained with anti‐mouse Alexa 568 (red). Nuclear and kinetoplast DNA were visualized using DAPI (blue). FLAG‐tagged TbRabex5 punctate staining localizes between the nucleus and kinetoplast in a region specific for endosomes. Scale bar: 5 μm. N‐terminal HA tagged TbRabex5 was expressed in BSF cells independently to control for mistargeting. HA‐TbVps9 is localized to punctate structures between nucleus and kinetoplast similar to FLAG‐tagged protein. (C) TbAlsin was ectopically expressed in BSF cells with N‐terminal HA tag (as in B), and show cytosolic staining. The lower panel shows BSF 427 cells alone as a negative control to rule out the possibility of non‐specific binding of anti‐HA antibody

TbAlsin and TbRabex5 were ectopically tagged to determine their subcellular locations. Immunofluorescence in BSF cells localized FLAG::TbRabex5 to puncta between the nucleus and kinetoplast, the region of the cell containing the flagellar pocket, endosomes and Golgi complex. In the vast majority of cells two separate puncta were observed: one closer to and anterior to the flagellar pocket and the other deeper and posterior to nucleus (Figure 4B). To rule out the possibility of mistargeting because of the epitope tag, HA‐tagged TbRabex5 was also localized, with very similar localization to the FLAG‐tagged form (Figure 4B). Furthermore, localization of this protein by endogenous genome tagging at TrypTag (http://tryptag.org) in the insect form is consistent with this location. The localization of TbRabex5 is similar to metazoan Rabex5, which resides on endosomes.^52^ Western blotting of cell lysates of cells expressing tagged TbRabex5 detected an antigen of 66 kDa, consistent with the predicted molecular weight (Figure 4A).

HA::TbAlsin was predominantly cytosolic (Figure 4C). Significantly, the human orthologues ALS2 and ALS2CL are also cytosolic in a majority of cells when overexpressed separately, but both MORN Vps9 GEFs localize to membranous compartments when coexpressed.^27^, ^53^, ^54^ Western blotting for HA‐epitope tagged TbAlsin identified a band of the expected size of 103 kDa (Figure 4A). Further analysis of the location of this protein proved inconclusive and the possibility that the protein is mislocalized because of overexpression cannot be excluded. Significantly localization by genomic tagging in the insect stage at TrypTag is consistent with endomembrane targeting, supporting this possibility (http://tryptag.org). Ectopic expression of both TbRabex5 and TbAlsin had no significant impact on proliferation (data not shown).

**Figure 5:**
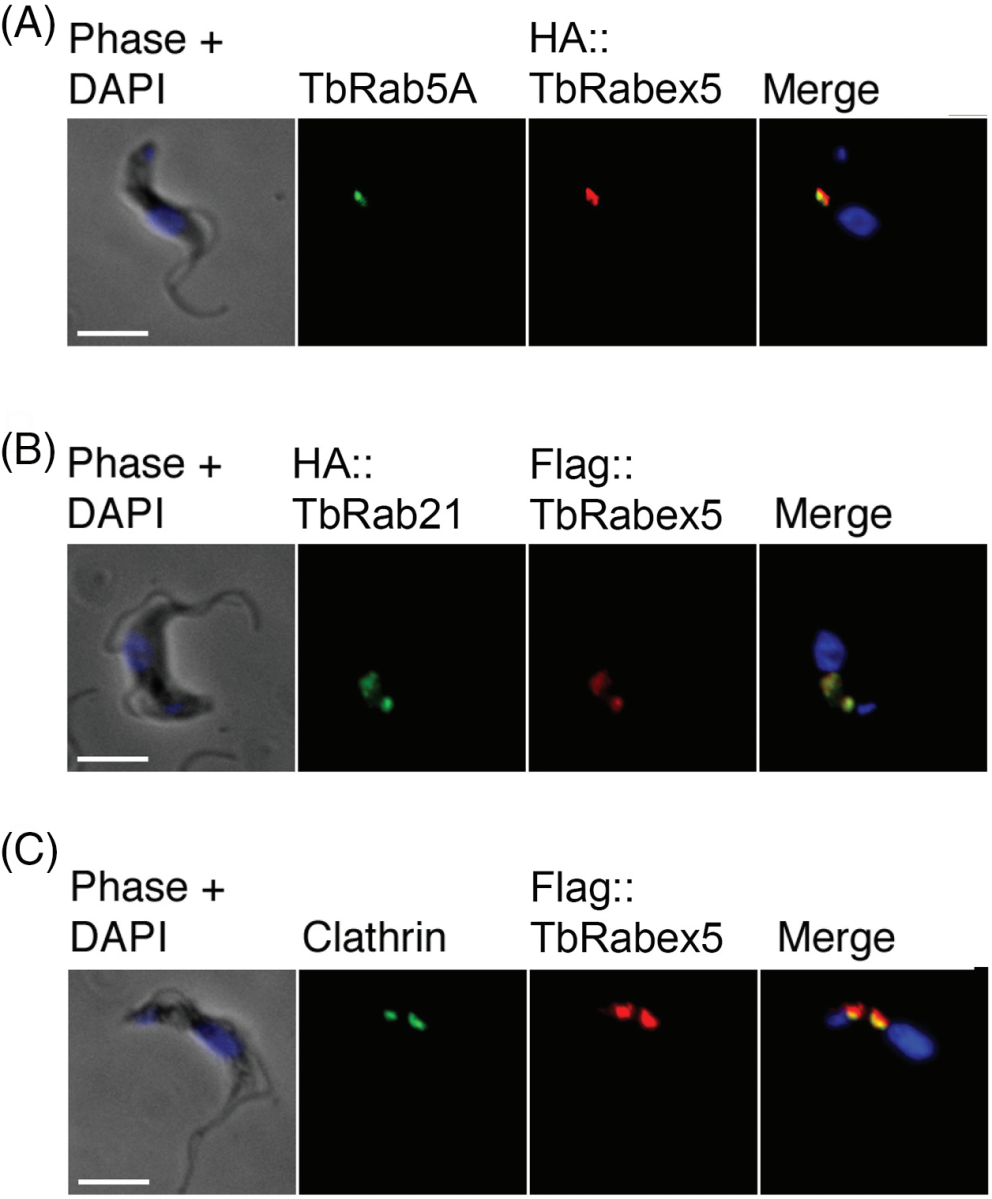
Colocalization of Vps9 domain proteins with known endosomal markers. BSF cells stably expressing TbRabex5 with N‐terminal HA or FLAG epitopes were co‐stained with markers of T. brucei endomembrane system. Cells were labelled with primary antibodies against epitope tags protein in addition to (A) TbRab5A, (B) HA‐tagged TbRab21 and (C) clathrin heavy chain. Proteins were visualized using appropriate Alexa Fluor conjugated secondary antibodies whereas DNA was visualized using DAPI. All images were captured at the same magnification. Scale bar: 5 μm

To determine the likely compartments with which TbRabex5 associated, cells were co‐stained with known *T. brucei* endosomal markers using specific antibodies. TbRabex5 partially overlapped with TbRab5A; a well characterized marker of early endosomes.^55^ In *T. brucei* early endosomes reside closer to the nucleus^56^ where TbRabex5 exhibited partial coincidence, while a distinct population was also seen close to the flagellar pocket in some cells (Figure 5A). Co‐staining of TbRabex5 and TbRab21 was performed using cells expressing tagged versions of both proteins, and tagged TbRab21 appeared to colocalize with TbRabex5 in the vast majority of cells (Figure 5B). In the absence of 3D reconstruction complete colocalization cannot be inferred, but the size, shape and number of puncta showing coincidence staining were identical for both TbRabex5 and TbRab21. The FLAG‐tagged TbRabex5 also showed partial colocalization with clathrin heavy chain, which was expected as clathrin associates with multiple endosomal compartments in trypanosomes (Figure 5C).^57^ If TbRabex5 is indeed a GEF for endocytic Rabs, the flagellar pocket localization and proximity/colocalization with TbRab5 and TbRab21 could indicate a role in Rab activation at sites of endocytic vesicle formation.

### 2.10 Roles of TbAlsin and TbRabex5 in endocytosis

**Figure 6:**
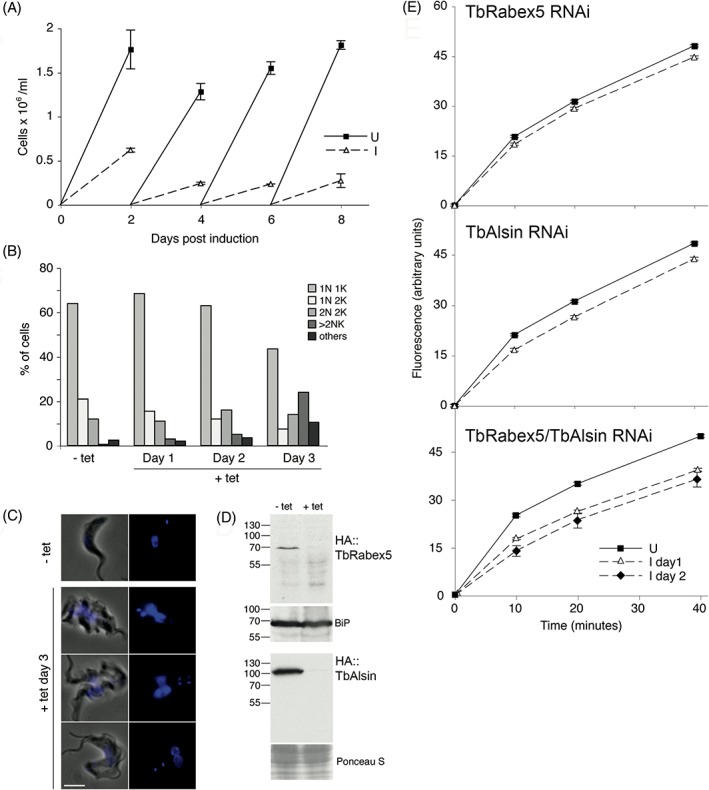
Effect of TbRabex5/Alsin double knockdown on BSF proliferation. (A) Growth curve of TbRabex5/Alsin double knockdown BSF parasites. SMB cells were transfected with RNAi plasmid p2T7 containing fused fragments of TbRabex5 and TbAlsin. RNAi cells were grown in the presence (broken lines) or absence (solid lines) of 1 μg/mL tetracycline and counted every 48 hours. Error bars shown are the SE of duplicate inductions. (B) Depletion of both Vps9 domain proteins results in a mild cytokinesis block. Cells were induced for 3 days, and a sample of cells fixed daily and stained using DAPI to visualize nuclei and kinetoplasts. At least 200 cells were observed under the microscope to categorize as 1N1K, 1N2K, 2N2K and cells having >2N or >2K. Cells that deviated from these categories were labelled “others”. (C) Phase and DAPI images of cells at day 3 of induction. Uninduced (− tet) cell shown as comparison to the variation observed in induced cells. Scale bar: 5 μm. (D) Validation of knockdown by western blot. The double knockdown cell line was transfected separately with N‐terminal HA‐tagged TbRabex5 or TbAlsin. Cells expressing tagged proteins were induced for RNAi and lysates were subjected to SDS‐PAGE followed by western blot using anti‐HA antibody. Both of the Vps9 domain proteins are shown to be lost specifically after induction. Loading control is shown as BiP or Ponceau S staining. (E) Concanavalin A uptake upon individual and double Vps9 knockdown. RNAi 1N1K cells were grown in the presence (broken lines) or absence (solid lines) of tetracycline, for 1 (triangles) and/or 2 days (diamonds), and fluorescein‐conjugated ConA uptake was measured. For each sample, 50 000 cells were counted and median fluorescence of ConA was plotted against time of uptake with error bars showing SE of median. (E, top) ConA uptake after 2 days of TbRabex5 RNAi induction, (E, middle) ConA uptake after TbAlsin RNAi and (E, bottom) ConA uptake upon TbRabex5/Alsin double knockdown

To gain functional insights into the roles of the 2 Vps9 proteins in trypanosomes, we silenced each using RNAi. For TbRabex5, a 50% reduction in the rate of BSF cell proliferation was observed following induction for 2 days (Figure S2C,D). Knockdown was validated by western blotting using the HA::TbAlsin RNAi cell line and after 24 hours induction TbRabex5 was undetectable. Similarly, knockdown of TbAlsin resulted in a roughly 50% reduction in BSF proliferation, with an >80% reduction in the level of HA‐tagged protein after 24 hours of induction (Figure S2E,F). Significantly, neither of these individual knockdowns resulted in an observable morphological defect. As Vps9 domain proteins in other systems are frequently redundant in terms of GEF activity against Rab5, a double knockdown was attempted. When induced, these TbAlsin/TbRabex5 knockdown cells exhibited a stronger proliferative defect compared to single knockdowns (Figure 6A), and western analysis revealed complete loss of both proteins after 24 hours of RNAi (Figure 6D). While it is possible that the defect observed in the double knockdown cells is the result of a synthetic interaction, this is unlikely to be the case given the high efficiency of both single knockdowns.

The morphology and cell cycle progression of the double Vps9 knockdown cells was examined daily post‐induction. By day 3 cultures showed an accumulation of cells with multiple kinetoplasts and nuclei (>2K/2N), compared to uninduced control cultures (Figure 6B). This accumulation suggested a cytokinesis defect but the late emergence of the phenotype also suggested a potential secondary defect. Enlargement of the flagellar pocket—the big eye phenotype related to silencing of early endosomal proteins like TbRab5A, TbRab5B and clathrin^58^, ^59^—was not observed following silencing of trypanosome Vps9 domain proteins, either individually or in combination (Figure 6C). This suggests that bulk membrane endocytosis is unimpaired, despite apparent localisation to the endosomes for TbRabex5 and probably also TbAlsin from TrypTag data.

We also monitored uptake of Concanavalin A (ConA), a mannose binding lectin that essentially reports on variant surface glycoprotein uptake.^59^ Uptake was unaffected in cells where TbRabex5 or TbAlsin were depleted by RNAi, but by contrast the double knockdown did result in a significant reduction in ConA uptake as early as 1 day post‐induction (Figure 6E). Reduced uptake became even clearer on day 2 and indicates a defect in endocytosis when both of the Vps9 domain proteins of *T. brucei* were ablated. This also suggests that the 2 Vps9 proteins in *T. brucei* likely have at least partly redundant functions.

**Figure 7:**
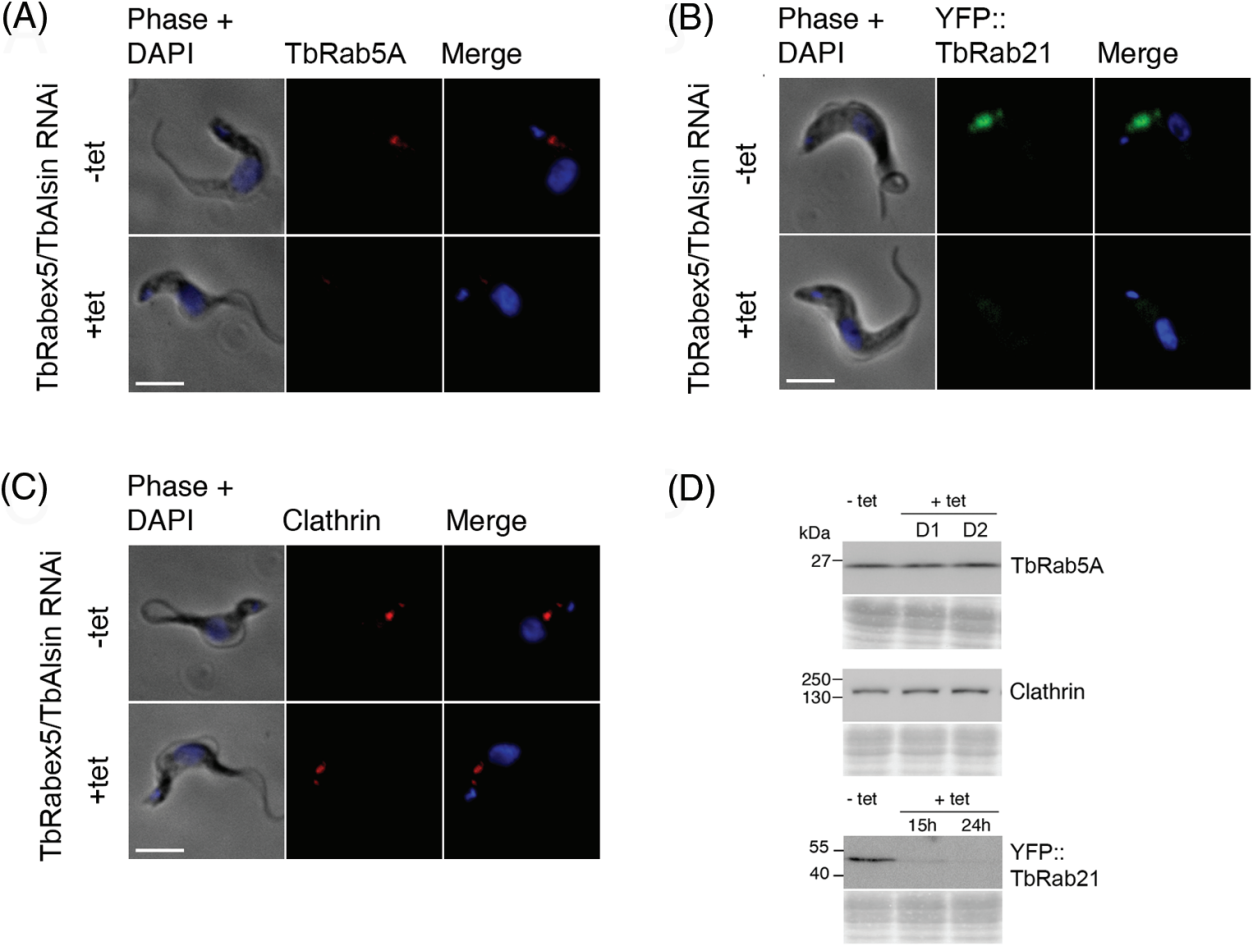
Double knockdown of TbVps9 proteins leads to loss of endosomal localization of both TbRab5A and TbRab21. (A) Immunofluorescence analysis of TbRab5A in TbVps9 double knockdown cells. Cells were fixed and stained with polyclonal antibodies against TbRab5A. Alexa Fluor 568‐labeled antibodies were used to visualize Rab5A and DNA was counterstained with DAPI. TbRab5A was reduced significantly from endosomes of induced cells. Scale bar: 5 μm. (B) Analysis of TbRab21 in TbVps9 double knockdown cells. SMB BSF TbVps9 double knockdown cells were transfected with N‐terminal YFP‐tagged TbRab21. Knockdown cells expressing recombinant YFP‐Rab21 were selected and induced for 15 hours to deplete both of TbVps9 domain proteins. Cells showed a significant reduction in the expression and endosomal localization of YFP tagged TbRab21 following 15 hours of Vps9 double knockdown. (C) IFA of clathrin in TbVps9 double knockdown cells. Clathrin location and expression remained unchanged even at a later post‐induction time of 48 hours. (D) Following knockdown of both of TbRabex5 and TbAlsin proteins, expression levels of TbRab5A, YFP‐TbRab21 and clathrin heavy chain were quantified. Averages of multiple inductions (duplicate/triplicate) were normalized to uninduced (100%). Error bars show SE of mean

To further explore this, we analysed the impact on the locations and expression levels of clathrin, TbRab5A and TbRab21 of knocking down TbRabex5 and TbAlsin. The loss of a GEF for a Rab results in decreased Rab activation and the inactive RabGDP tends to lose membrane localization, resulting in dispersal in the cytosol.^9^, ^20^ Specifically, Rab5 GEF defects are associated with a reduction in the size of early endosomes in *C. elegans* cells.^44^ Knockdown alone of either Vps9 protein failed to elicit any change in the endosomal distribution or total protein levels of clathrin heavy chain, TbRab5A or TbRab21 (Figures S3 and S4). By contrast, in a double knockdown, there was 50% reduction (*P* < .005, *n* = 40) in the amount of endosome‐associated TbRab5A compared to uninduced cells at 24 hours, whereas the total amount quantified by western blotting remained unchanged (Figure 7A,D). We presume that the Rab5A proteins that lost their membrane localization were not detectable in the cytoplasm as a result of the IFA staining procedure. Significantly, a rapid loss of TbRab21 was also observed by western blotting and in this case TbRab21 reduced to 10% of the uninduced level after 15 hours, and confirmed by immunofluorescence (Figure 7B,D). We suggest that these reductions in Rab5A and Rab21 endosomal association explain the reduced uptake of ConA. By contrast no change in the amount or subcellular distribution of clathrin was observed (Figure 7C,D), suggesting a very specific effect on early endosome functions and also consistent with the absence of an enlarged flagellar pocket.

### 2.11 Physical interaction between TbVps9 proteins and endocytic Rabs

**Figure 8:**
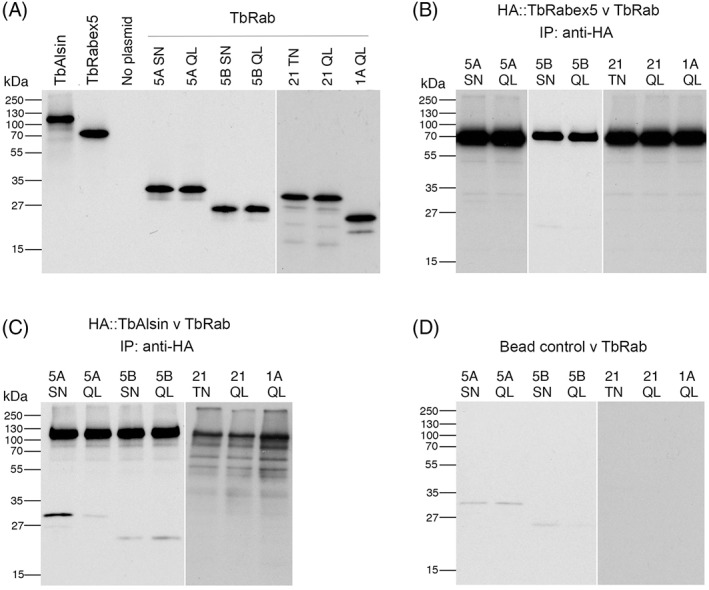
TbAlsinVps9 interacts specifically with GDP bound mutant of TbRab5A. (A) in vitro expression of TbVps9 proteins and TbRabs. Vps9 domain proteins are 20% of CoIP input while Rabs are 5%. No plasmid served as a control for reticulocyte lysate background labelling. All the autoradiographs were exposed for comparable time and rearranged for clarity. (B) Coimmunoprecipitation of TbRabex5 (bait) and a set of endosomal TbRabs (prey). Equal volumes of in vitro expressed bait and prey proteins were mixed and incubated for binding, following pull down using anti‐HA polyclonal antibodies. Proteins were eluted and separated on SDS‐PAGE followed by autoradiography. QL Rabs are GTP‐bound mutants while SN/TN are GDP‐bound mutants. Rab1A served as negative control. TbRabex5 full‐length protein was not able to bind any of the mutant Rabs under the conditions of the experiment. (C) Coimmunoprecipitation of TbAlsin (bait) and a set of endosomal TbRabs (prey). Equal volumes of in vitro expressed bait and prey proteins were incubated for binding and harvested with anti‐HA polyclonal antibodies. TbAlsin interacted with inactive GDP (SN) mutants of TbRab5A but not the QL mutant. Note: Expression of TbAlsin in Rab1A and Rab21 experiments is poor but was sufficient to pull down TbRab5A^SN^ in a separate experiment. (D) Controls for non‐specific bead/antibody binding. in vitro expressed TbRabs were pulled down using the same quantities of anti‐HA polyclonal antibodies and protein A beads as described for panels B and C

To search for direct evidence of interactions between trypanosome Vps9 domain proteins and endocytic Rabs coimmunoprecipitation experiments were performed. As the Rab GEFs bind only with inactive GDP‐bound Rabs, QL mutants of endocytic Rabs (GTP locked, active Rab mutant) and SN mutants (GDP locked, inactive Rab mutant) were analysed. TbAlsin and TbRabex5 were coexpressed in reticulocyte lysates with a cohort of Rabs including TbRab1A, TbRab5A, TbRab5B, TbRab21 and TbRab28. An interaction between TbRab5A^SN^ and TbAlsin was detected while the QL mutant failed to interact as expected. The result was highly reproducible and based on the number of [^35^S]‐labelled amino acids in each protein ~50% of TbAlsin bound to TbRab5A (Figure 8).

## 3 DISCUSSION

Vps9 domain‐containing proteins are central players in endocytosis in mammalian and yeast cells. While Vps9 proteins had been identified in a handful of other taxa, the full breadth of their diversity was unknown. Here we identified Vps9 domain proteins encoded in over 50 genomes and transcriptomes across the eukaryotic tree. Only 3 organisms had no identifiable Vps9 domain‐containing protein. The genomes of these 3 organisms are highly reduced because of adaptation; parasitism for *G. intestinalis,* 60 and extremophilia in the closely related red algae *C. merolae* and *G. sulphuraria.* 61 Furthermore, neither *Giardia* nor *C. merolae* possess known orthologues of Rab5 or Rab21,^1^, ^2^, ^62^ suggesting a loss of this entire arm of the endocytic pathway. By contrast, most genomes sampled encode more than 1 Vps9 domain‐containing protein, suggesting similar importance in other eukaryotes as for human cells. However, there does not appear to be any correlation with the presence of Rab21 and Rab22 and either number or type of Vps9 family proteins in eukaryotes. In a study of the Tre‐2/Bub2/Cdc16 (TBC)‐domain RabGAPs, it was noted that the GTPase regulators were found at a lower copy number than Rabs in a given genome,^4^ suggesting that the RabGAPs act promiscuously. This may also be the case for Vps9 proteins, raising the possibility of interactions with Rabs outside the Rab5 family, and additional roles in the endocytic system.

**Figure 9:**
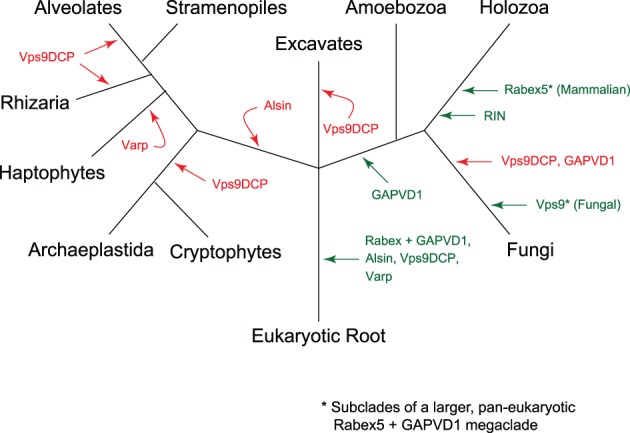
Evolution of the Vps9 protein family in eukaryotes. Gains are denoted by green arrows, whereas losses are denoted by red arrows. Although the fungal Vps9 clade and mammalian Rabex5 clade are part of a larger Rabex5‐like clade, they are shown here separately, as they have been studied independently and extensively in the literature

**Figure 10:**
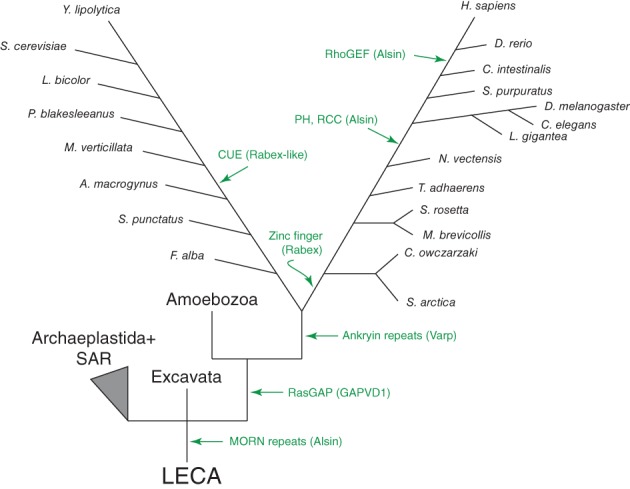
Evolutionary timeline of acquisition of domains present in mammalian Vps9 family proteins. Green arrows show the earliest known gain of domains for each protein subfamily shown in brackets. Archaeplastida and SAR are shown as a collapsed node for simplicity. While some domains were acquired recently in mammalian evolution, others predate supergroup divergence events or were present in the LECA

There were at least 3 primordial clades of Vps9 domain proteins in the LECA: Alsin, Varp and Rabex5 + GAPVD1 (Figure 9). The additional domains present in the human homologues of these proteins appear to have been acquired later in eukaryotic evolution, as have the non‐animal lineage‐specific domain acquisitions (Figure 10). However, there are 2 major exceptions of domains present in human Vps9 proteins that are found outside of the Opisthokonta, one being the MORN repeats found in the excavate and some amoebozoan Alsin homologues (Figure 10). The second exception is the domain acquisition in the Rabex + GAPVD1 subfamily. Based on our phylogenetic analyses, a Rabex + GAPVD1 preduplicate was likely present in the LECA (Figure 1 and Figure S1). We propose that a duplication of the Rabex + GAPVD1 sequence occurred at the base of the Amorphea (Amoebozoa and Opisthokonta), generating the GAPVD1 subfamily. Furthermore, a RasGAP domain—a defining feature of the GAPVD1 sequence in animals—is present in holozoan and amoebozoan representatives. It is also present in the sole GAPVD1 representative of the holomycotan *F. alba*, prior to the loss of the protein in the Fungi.

In animal cells, Varp is characterized by a Vps9 domain and 2 sets of ankyrin repeats with upstream CHPLCxCxxC motifs, and this architecture is conserved generally in the Holozoa.^21^ While the cysteine‐rich motif appears to be restricted to the Holozoa, a Varp‐like homolog with ankyrin repeats was likely present in the ancestor of opisthokonts, although not in the outgroup taxon *T. trahens.* In human cells, the CHPLCxCxxC motif is responsible for recruiting Varp to endosomes by interaction with the retromer subunit Vps29,^21^ suggesting this interaction is not conserved outside of the Holozoa. Furthermore, work in human cells has shown that the first ankyrin repeat of Varp binds Rab32, and is involved in melanogenic enzyme trafficking.^22^ Despite the fact that Rab32 is lost from the Fungi, a putative Rab32 orthologue is present in the basal *F. alba* (XP_009497485). Given that the Varp orthologue in *Fonticula* is the only holomycotan orthologue to also contain an Ankyrin‐repeat, this suggests that the binding of Rab32 and Varp is conserved from the opisthokont ancestor onwards but was lost in Fungi.

The human Rabex5 and yeast Vps9, on the other hand, appear to be members of 2 distinct clades within the Rabex‐like proteins (Figure S1R). There have also been 2 clear duplications of Rabex‐like proteins in the Amoebozoa. The Rabex‐like protein in Fungi, which includes the Vps9p protein of *S. cerevisiae*, acquired a CUE domain prior to the divergence of *Mortierella.* At the base of Holozoa, the Rabex5‐like protein duplicated, and 1 duplicate acquired the ubiquitin‐binding zinc finger domain present in the mammalian Rabex5 (Figure 10). This acquisition is relatively ancient in the Holozoa, occurring prior to the divergence of the basal filasterian *C. owczarzaki.* The other duplicate, however, was maintained until the base of animals, and appears to have been lost in the ancestor of *Trichoplax adhaerens.*

In addition to the zinc finger and CUE domains present in some animal and fungal representatives, a third independent acquisition of an ubiquitin‐binding domain is in the amoebozoan *Sexangularia* sp., which has a sequence grouping within an Amoebozoa‐specific clade that contains a domain of the ubiquitin associated (UBA) domain‐like superfamily. The tendency for these proteins to gain and conserve ubiquitin‐binding domains suggests the relevance of ubiquitin binding to Vps9 family protein function.^63^ In human cells, the zinc finger is required for recruitment to early endosomes independent of both Rab5 interaction and GEF activity, which raises the possibility of whether Rabex + GAPVD1 homologues with other ubiquitin‐binding domains are similarly localized.

We identified a clade of Vps9 DCPs that are patchily conserved in eukaryotes, finding orthologues in the Fungi, Stramenopile and Haptophyte clades, suggesting that a Vps9DCP‐like orthologue was present in the LECA. To date, the human orthologue remains functionally unclassified, and contains only a Vps9 domain. However, generally in the Vps9 family, there are numerous examples of lineage‐specific domain acquisitions; many of which are known to interact with membrane or cytoskeletal components in human cells, while others have more diverse functions.

Investigation of the functions of 2 Vps9 proteins in trypanosomes suggests a broadly conserved role within the Excavata. Specifically, Vps9 proteins from 2 distinct subfamilies, represented by TbRabex5 and TbAlsin, both likely localize to endosomes, and data are consistent with interactions with TbRab5 and TbRab21. Furthermore, knockdown of individual transcripts suggests redundancy, but a double knockdown impacts the ability of both TbRab5A and TbRab21 to target correctly, consistent with GEF activity, while we were also able to obtain evidence for a direct interaction between TbAlsin and TbRab5A.

Based on our observations of Vps9 protein evolution in eukaryotes, we propose 2 overarching qualities of this family: Vps9 family proteins serve as scaffold proteins of the endocytic system, and they can act an evolutionary platform for endocytic specialization. Given other evidence of interaction between Vps9 domain proteins and Rab5 homologues in members of the archaeplastids^9^ and SAR clades,^12^ as well as our novel data in excavates, there appears to be a remarkable level of conservation in terms of control mechanisms for endosomal systems.

## 4 METHODS

### 4.1 Comparative genomics and evolutionary inference

Human orthologues of all Vps9 domain‐containing proteins were used as queries: HsVarp (NP_115515.2), HsRIN2 (NP_061866.1), HsVps9 (NP_004904.2), HsAlsin (NP_065970.2), HsRabex (NP_055319.1) and HsGAPVD1 (NP_056450.2). BLASTP^64^ searches were performed to search the predicted proteomes of the following organisms: *Gallus gallus*, *Drosophila melanogaster*, *Ciona intestinalis*, *Strongylocentrotus purpuratus*, *Monosiga brevicollis* and *C. owczarzaki.* Proteins were considered orthologous if they retrieved the query sequence or a clear orthologue as the top hit in the reciprocal BLAST search, and proteins from both forward and reciprocal searches were retrieved with an *E*‐value less than 0.05. The domain structure of all Vps9 domain proteins was determined by searching the conserved domain database (CDD),^65^ and similar domain organization of the candidate proteins to the human orthologue supported the relationship.

Positively identified Vps9 homologues from holozoan organisms were used to generate Vps9 protein‐specific hidden Markov models, which were then used to search a diversity of eukaryotes listed in Table S1. For the following amoebozoan organisms, translated MMETSP (Marine Microbial Environmental Transcriptome Sequencing Project)^66^ transcriptomes were searched, as this group lacks genomic representation: *Vannella* sp., *Paramoeba* sp., *Neoparamoeba* sp., *Sexangularia* sp., *Mayorella* sp., *Filamoeba nolandi* and *Pessonella* sp. To ensure that we did not bias the HMMer search towards holozoan organisms, we also used the hidden Markov model generated for Vps9 available through the Pfam protein families database^67^ to search the predicted proteomes. As in Holozoa, the domain structure of all Vps9 domain‐containing proteins was determined by the CDD.

Throughout this work, we use the tripartite classification of eukaryotes outlined by Adl et al,^17^ which mainly splits organisms into the Amorphea (Amoebozoa, Opisthokonta supergroups), Diaphoretickes (Stramenopile, Alveolata, Rhizaria clade; Archaeplastida; and Cryptophyta, Centrohelida, Telonemia and Haptophyta) and the Excavata. Based on our current understanding of the root of eukaryotes,^68^, ^69^ if a Vps9 family protein is found in representatives of the Amorphea and the Excavata sampled in this paper, we can infer that it was present in the LECA, and lost in the Diaphoretickes.

### 4.2 Phylogenetics

Because of the high sequence divergence and relatively short Vps9 domain, initial phylogenetics attempts generated trees with no resolution. We therefore undertook a multi‐step approach to sequence classification. As the human Vps9 family proteins are functionally characterized, we first generated a tree using well‐classified metazoan sequences with strong backbone support. Then, individual Vps9 domain proteins from other supergroups (or subclades) were aligned to this metazoan backbone alignment, and trees were generated. Finally, we performed a Scrollsaw‐style analysis^1^ in which the shortest branching sequences from each clear clade in each tree were selected for phylogenetic analysis.

For the initial metazoa backbone tree, all identified metazoa Vps9 domain‐containing proteins were aligned using MUSCLE v3.8.31,^70^ with extensive manual realignment using the alignment visualization program Mesquite v3.03.^71^ For all other trees, Vps9 sequences from each group were iteratively aligned to the metazoan alignment using the profile option in MUSCLE. Non‐homologous positions were manually masked and trimmed. Masked alignments are available upon request. Model‐testing was performed using ProtTest v3.4^72^ to identify the most accurate model of sequence evolution. The model parameters for each tree are available in Table S1.

Phylobayes v4.1^73^, ^74^, ^75^ and MrBAYES v3.2.2^76^ programs were run for Bayesian analysis and RAxML v8.1.3^77^ was run for maximum‐likelihood analysis. Phylobayes was run until the largest discrepancy observed across all bipartitions was less than 0.1 and at least 100 sampling points were achieved, MrBAYES was used to search treespace for a minimum of 1 million Markov chain Monte Carlo (MCMC) generations, sampling every 1000 generations, until the average SD of the split frequencies of 2 independent runs (with 2 chains each) was less than 0.01. Consensus trees were generated using a burn‐in value of 25%, well above the likelihood plateau in each case. RAxML was run with 100 pseudoreplicates.

For the final scrollsaw tree, the shortest branching sequences for each clade in the MrBAYES trees were selected. In cases where multiple trees were generated for each supergroup, we compared branch lengths relative to the length of the human sequence HsRIN2 to the base of the RIN clade, as the RIN clade is restricted to the metazoa and therefore the branch length of this sequence within this clade should remain relatively constant between trees. We then iteratively aligned these sequences to the metazoan alignment as described above, and then removed all extraneous metazoan sequences for this final tree. Trees were then run as described above. For all trees, long‐branching sequences were removed to improve resolution.

A separate set of trypanosome‐specific classification trees were generated, as the excavate phylogenies were poorly resolved. In these trees, only the 2 *T. brucei* and *T. cruzi* Vps9 sequences were aligned to the metazoa alignment. Otherwise, trees were generated as described above.

### 4.3 Culturing of bloodstream and procyclic forms of T. brucei

*T. brucei* blood stream form cells (Lister 427) were cultured in HMI‐9 complete medium with 10% tetracycline free heat inactivated foetal bovine serum (FBS), 100 U/mL penicillin, 100 U/mL streptomycin and 2 mM l‐glutamine at 37°C with 5% CO_2_ in non‐adherent culture flasks, with vented caps.^78^ For RNAi experiments single marker bloodstream (SMB) cells expressing T7 RNA polymerase^79^ were cultured under the same conditions in the continuous presence of 5 μg/mL neomycin and expression of double‐stranded RNA was induced by the addition of tetracycline at 1 μg/mL. All BSF cell lines were maintained below a culture density of 2 × 10^6^ cells/mL. To determine cell density, 100 μL aliquots were withdrawn from cultures and diluted with 10 mL isoton II (Beckman Coulter), cell number was determined with a Z2 Coulter Counter (Beckman Coulter), averaging 3 measurements. A hemocytometer was also used for counting cells. Procyclic form cells were cultured in SDM79 media supplemented with 10% FBS, penicillin, streptomycin, l‐glutamine and haemin.^80^ Cells were cultured in non‐vented flasks at 27°C and maintained between 1 × 10^5^ and 2 × 10^7^ cells/mL.

### 4.4 Quantitative real‐time polymerase chain reaction

First strand cDNA was synthesized using SuperScript III reverse transcriptase (Invitrogen) following the manufacturer's instructions. Two microgram of total RNA was made up to 11 μL with nuclease‐free water, 1 μL of 50 μM oligo (dT)18 and 1 μL of 10 mM dNTPs. The mixture was heated to 65°C for 5 minutes and incubated on ice for at least 1 minute. To this denatured RNA mixture 4 μL of ×5 first strand buffer (250 mM Tris‐HCl, pH 8.3 at room temperature, 375 mM KCl, 15 mM MgCl_2_) 1 μL of 0.1 M DTT, 1 μL RNaseOUT (recombinant RNase inhibitor, Invitrogen) and 1 μL of SuperScript III reverse transcriptase added before incubating at 50°C for 60 minutes to synthesize cDNA. The reaction was inactivated by heating at 70°C for 15 minutes. cDNA was diluted with Milli‐Q water and used as a template for amplification. Quantitative real‐time polymerase chain reaction (qRT‐PCR) was performed using BioRad's iQTM SYBR Green Supermix, following the manufacturer's instructions. Briefly, 12.5 μL of ×2 iQTM SYBR Green Supermix (×2 reaction buffer with dNTPs, iTaq DNA polymerase, 6 mM MgCl_2_, SYBR Green I, fluorescein and stabilizers), 0.4 μM of forward and reverse primers and cDNA equivalent to 50 to 100 ng total RNA (depending on the expression level of the genes under investigation) was mixed and the final volume made up to 25 μL with nuclease‐free water. Replicate samples were assembled as a master mix with a single addition of different templates. The reaction mix was transferred into multiplate PCR plates and set on a MiniOpticon real‐time PCR system, both from Bio‐Rad. Amplification profile was: initial denaturation at 95°C for 3 minutes followed by 40 cycles at 95°C for 30 seconds, 58°C for 30 seconds and 72°C for 30 seconds. Finally, a melting curve from 60 to 95°C was obtained to ascertain the specificity of the PCR amplification. The normalized expression (ΔΔ*Ct*) of mRNA was subsequently determined (based on one or multiple reference genes) using Bio‐Rad CFX manager software.

### 4.5 RNAi constructs and transfections

RNAi constructs were made by cloning 400 to 600 bp fragments, selected using RNAit^81^ into Eam 11051 (Fermentas) linearized p2T7‐TAblue vector. This tetracycline inducible T7 RNAi expression vector was a derivative of p2T7^82^ with blue white screening capability. For ectopic expression pHD1034^83^ or pXS5^55^ were used. Both have rRNA promoters and were used to clone genes of interest either in frame to an N‐terminal YFP, HA or FLAG epitope tags. pHD1034 was used for overexpression in both life forms where pXS5 was specific for BSF.

### 4.6 Plasmid construction and transfection

pGBKT7 and pGADT7 vectors (Matchmaker system, Clontech Laboratories, Inc.) were used to express *T. brucei* Rabs and Vps9 proteins in vitro, using their T7 promoters. Mutant Rab (QL GTP locked, active Rab mutant and SN GDP locked, inactive Rab mutant) pGBKT7 constructs were a kind gift from Carme Garbernet‐Castello, while TbRab21 was cloned into pGBKT7 downstream of the c‐Myc epitope tag using appropriate restriction sites from the multiple cloning sites (MCS) and subsequently mutated using the In‐Fusion Advantage PCR cloning kit into inactive Rab21TN mutant. *E. coli* were transformed and positive clones were selected using 50 μg/mL kanamycin. *T. brucei* GEFs (TbRabex5 Tb927.10.10020 and TbAlsin Tb927.3.2430) were cloned in pGADT7‐AD vector. Appropriate restriction sites from MCS were used for in‐frame cloning of trypanosome GEFs with respect to the HA epitope tag. Positive clones were selected using 100 μg/mL ampicillin and all constructs were sequenced prior to further use.

*T. brucei* bloodstream form cells were transfected with 10 μg linearized plasmids. An Amaxa Nucleofector device was used for transfection together with the Amaxa Human T‐cell nucleofector kit, following manufacturer's instructions with few modifications. About 30 to 50 million mid‐log phase BSF cells were harvested per transfection and resuspended in 100 μL of nucleofector solution provided in the kit and mixed with 10 μg linearized plasmid (dissolved in 5‐10 μL of sterile water). Transfection was performed in Amaxa cuvettes using program X‐001 (optimized electrical parameters). Transfected cell were serially diluted and after ~6 hours of posttransfection incubation, 1 mL aliquots were transferred to 24 well plates where the cells were selected for 5 to 6 days using antibiotics (BSF; neomycin G418: 2.5 μg/mL, hygromycin: 2.5 μg/mL, puromycin: 0.2 μg/mL, phleomycin: 1 μg/mL). Clones were picked from appropriately diluted plates and maintained under continuous drug pressure.

To transfect PCF, exponentially growing cells were harvested and washed once with cytomix (2 mM EGTA, 120 mM KCl, 0.15 mM CaCl_2_, 10 mM KPO_4_, 25 mM HEPES, 5 mM MgCl_2_, 0.5% glucose, 100 μg/mL BSA, 1 mM hypoxanthine, pH 7.6) before resuspending 2 x 10^6^ cells in 500 μL of cold cytomix. Linearized plasmid DNA of 25 μg was added to the cells, mixed well and transferred to a cuvette for electroporation using a Bio‐Rad gene pulser set at 1.5 kV and a capacitance of 25 μF. Transfected cells were allowed to recover for 8 hours in 10 mL 20% FBS media before the addition of selective antibiotic (puromycin 1.5 μg/mL for pHD1034 vector). Cells were serially diluted and 150 μL aliquots plated into 96 well plates and grown for 2 weeks at 27°C. Clones were picked and expanded into larger volumes, maintaining above the minimum culture density. Stable transgenic lines (BSF and PCF) were frozen in appropriate growth media containing 10% glycerol and slowly cooled to −80°C using paper towel padding. Frozen cell vials were subsequently transferred to liquid nitrogen for long‐term storage.

### 4.7 Western blotting

For lysate preparation, cells were harvested by centrifugation at 800*g* for 10 minutes at 4°C, washed in PBS and resuspended in SDS sample buffer at a concentration of 0.5 × 10^6^ cell equivalents per microliter before heating at 95°C for 10 minutes. Samples were electrophoresed on 10% SDS‐PAGE minigels gels at 5 × 10^6^ cell equivalents per lane and then transferred to Immobilon PVDF membrane (Millipore) by wet blot using transfer buffer (192 mM glycine, 25 mM Tris and 20% v/v methanol). Ponceau S staining was performed to monitor loading, by shaking membranes in Ponceau S solution (0.1% Ponceau S w/v in 5% v/v acetic acid, Sigma) for 5 minutes and washing the excess stain with Milli‐Q water. Membranes were blocked for 1 hour at room temperature in blocking solution of 5% dried skimmed milk in TBST (24.8 mM Tris pH 7.4, 137 mM NaCl, 2.7 mM KCl, 0.2% Tween‐20). After blocking membranes were incubated from 1 hour at room temperature to over night at 4°C with primary antibody in blocking solution of 1% to 3% milk. Excess antibodies were washed by shaking membranes thrice for 5 minutes each in TBST before incubating for 1 hour with peroxidase goat anti‐rabbit conjugate or peroxidase rabbit anti‐mouse conjugate (secondary antibodies were from Sigma and used at a dilution of 1:10 000 to 1:20 000). Blots were washed again and bound antibodies were detected by reaction with luminol and visualized by exposure to Kodak chemiluminescence film or using G:BOX chemiluminescence imaging system from Syngene. For re‐probing where needed, membranes were stripped using Restore western blot stripping buffer (Thermo Scientific) following the manufacturer's instructions and processed for western blot.

### 4.8 Immunoprecipitation

To study the physical interaction between endocytic TbRabs and TbVps9 proteins, ^35^S‐Met‐labeled proteins were made by *in vitro* transcription and translation using Promega's TNT T7 coupled reticulocyte lysate system according to the manufacturer's instructions. Briefly, 1.5 μg purified plasmid (pGBKT7/pGADT7 constructs) was mixed with 40 μL of TNT master mix and 20 μCi of [35S] methionine. Nuclease‐free water was added to make the volume up to 50 μL. Recommended grade of 35S methionine (PerkinElmer EasyTag Methionine L‐[35S] specific activity 1000 Ci/mmol Cat No. NEG709A) was used to avoid background labelling of rabbit reticulocyte lysate. The reaction was incubated at 30°C for 90 minutes or 22°C in case of the larger TbAlsin protein. *In vitro* translation reactions were analysed by heat denaturing a small aliquot of translation reaction in SDS sample buffer and separating on SDS‐PAGE gels. Western blotting or autoradiography was used to detect in vitro translated proteins. Western blots were done using anti‐HA and/or anti‐c‐Myc antibodies. For autoradiography gels were agitated with fixing solution (50% methanol, 10% glacial acetic acid and 40% water v/v) for 45 minutes and with 10% glycerol for 5 minutes before drying at 80°C for 2 hours using a gel dryer. The dried gels were exposed on Kodak BioMax MR scientific imaging film for several days.

For coimmunoprecipitation, 10 μL of in vitro translated TbRabs and 10 μL TbVps9 proteins were mixed in different combinations and incubated for 1 hour at room temperature. Anti‐HA polyclonal antibody of 10 μL (Santa Cruz Biotechnology, Inc) was added to the protein mix and incubated for 1 hour at room temperature. About 3 μL of PBS washed protein A beads were added and reaction tubes were rotated for 1 hour at room temperature to capture the immune complexes. Tubes were centrifuged at 7000 rpm for 10 seconds and the supernatant was discarded before washing the beads 7 times with wash buffer (50 mM Tris, pH 7.5, 150 mM NaCl, 10 mM EDTA, 0.1% Triton X‐100, 10 mM MgCl_2_, Protease inhibitor; Roche complete 1 tab/50 mL). To elute and denature bound proteins, beads were heated with 20 μL SDS sample buffer at 80°C for 5 minutes, samples loaded onto SDS‐PAGE minigel and after electrophoresis autoradiography was performed as above.

### 4.9 Immunofluorescence microscopy

For immunofluorescence microscopy BSF or PCF cells were harvested by centrifugation at 800*g* for 10 minutes at 4°C. Pelleted cells were gently washed with ice cold Voorheis' modified PBS (vPBS; 136 mM NaCl, 3 mM KCl, 16 mM Na_2_HPO_4_, 3 mM KH_2_PO_4_, 40 mM sucrose, 10 mM glucose, pH 7.6)^84^ and fixed for 10 minutes for BSF or 60 minutes for PCF on ice in 3% formaldehyde. Excess fixative was washed with vPBS and fixed cells were adhered to polylysine‐coated slides (PolysineTM VWR International) sectioned with ImmEdge Pen (Vector Laboratories) for 20 minutes at room temperature. For permeabilization cells were incubated with 0.1% Triton X‐100 in PBS for 10 minutes, subsequently washed 3 times for 5 minutes each with PBS. Cells were blocked for 1 hour at room temperature in 20% FBS in PBS and incubated with primary antibodies diluted in 20% FBS. Unbound primary antibodies were washed off with PBS 3 times, 5 minutes prior to incubation with secondary antibodies: Oregon green 488 conjugate anti‐mouse, Alexa Fluor 488 anti‐rabbit, Alexa Fluor 568 anti‐mouse and Alexa Fluor 568 anti‐rabbit, for 1 hour at room temperature. All secondary antibodies were from Invitrogen and used at a 1:1000 dilution. Following incubation, cells were washed as before. Cells were air dried and coverslips mounted using Vectashield (Vector Laboratories, Inc) mounting medium with 4′,6‐diamidino‐2‐phenylindole (DAPI). Coverslips were sealed with nail varnish before observing under a Nikon ECLIPSE E600 epifluorescence microscope. Images were captured using a Hamamatsu ORCA charge coupled device camera. Digital images were analysed and false coloured using MetaMorph 6.0 software (Universal Imaging Corporation) and figures assembled from raw images using Adobe Photoshop CS3 (Adobe Systems).

### 4.10 Endocytosis assay

Log phase cells (5 × 10^7^) were harvested by centrifugation at 800*g* for 10 minutes at 4°C. Cells were wash once with 10 mL of serum‐free HMI‐9 1% BSA and resuspended gently in 5 mL serum‐free HMI‐9 1% BSA and incubated at 37°C for 30 minutes to serum starve. Fluorescein‐conjugated Concanavalin A or Alexa Fluor 633‐conjugated human transferrin was added to a final concentration of 10 and 125 μg/mL, respectively. Cells were incubated at 37°C for different periods of time, followed by the addition of ice‐cold vPBS to stop uptake. Cells were pelleted, fixed and mounted as for IFA or processed for flow cytometry.

## EDITORIAL PROCESS FILE

The Editorial Process File is available in the online version of this article.

## Supporting information


**Editorial Process**

**Figure S1.** Classification of eukaryotic Vps9 domain‐containing proteins.
**Figure S2.** TbRabex5 and TbAlsin expression in BSF and PCF cells, and the effects of their depletion on BSF proliferation.
**Figure S3.** Knockdown of TbRabex5 protein does not affect the expression or location of endosomal Rabs.
**Figure S4.** Knockdown of TbAlsin protein does not affect the expression or location of endosomal Rabs.
**Table S1.** Classification of eukaryotic Vps9 domain‐containing proteins.
