# Supplementary material for: Regulation of early endosomes across eukaryotes: Evolution and functional homology of Vps9 proteins
Source: Traffic. 2018 Apr 25;19(7):546–63. doi: 10.1111/tra.12570 (PMC6032885; doi:10.1111/tra.12570)
Supplement: Supplementary file 2 — Figure S1. Classification of eukaryotic Vps9 domain‐containing proteins. Figure S2. TbRabex5 and TbAlsin expression in BSF and PCF cells, and the effects of their depletion on BSF proliferation. Figure S3. Knockdown of TbRabex5 protein does not affect the expression or location of endosomal Rabs. Figure S4. Knockdown of TbAlsin protein does not affect the expression or location of endosomal Rabs. Table S1. Classification of eukaryotic Vps9 domain‐containing proteins. [file TRA-19-546-s001.pdf]

**Regulation of early endosomes across eukaryotes: Evolution and functional homology of Vps9 proteins**

**Running Title:** Vps9 evolution in eukaryotes

Emily K. Herman[1*], Moazzam Ali[2], Mark C. Field[2*], Joel B. Dacks[1]

[1]Department of Cell Biology, Faculty of Medicine and Dentistry, University of Alberta, Edmonton, Alberta, Canada
[2]School of Life Sciences, University of Dundee, Dundee, DD1 5EH, UK

Keywords: Membrane-trafficking, endosomes, Trypanosoma, phylogeny, Rab, LECA

***Authors for correspondence:**

Mark C. Field (trypanosomes), Division of Biochemistry and Drug Discovery, School of Life Sciences, University of Dundee, Dundee, Scotland, +44 (0)751-550-7880, mfield@mac.com

Emily K. Herman (molecular evolution), Department of Cell Biology, Faculty of Medicine and Dentistry, University of Alberta, Edmonton, Alberta, Canada, eherman@ualberta.ca

**Supplementary Figures**

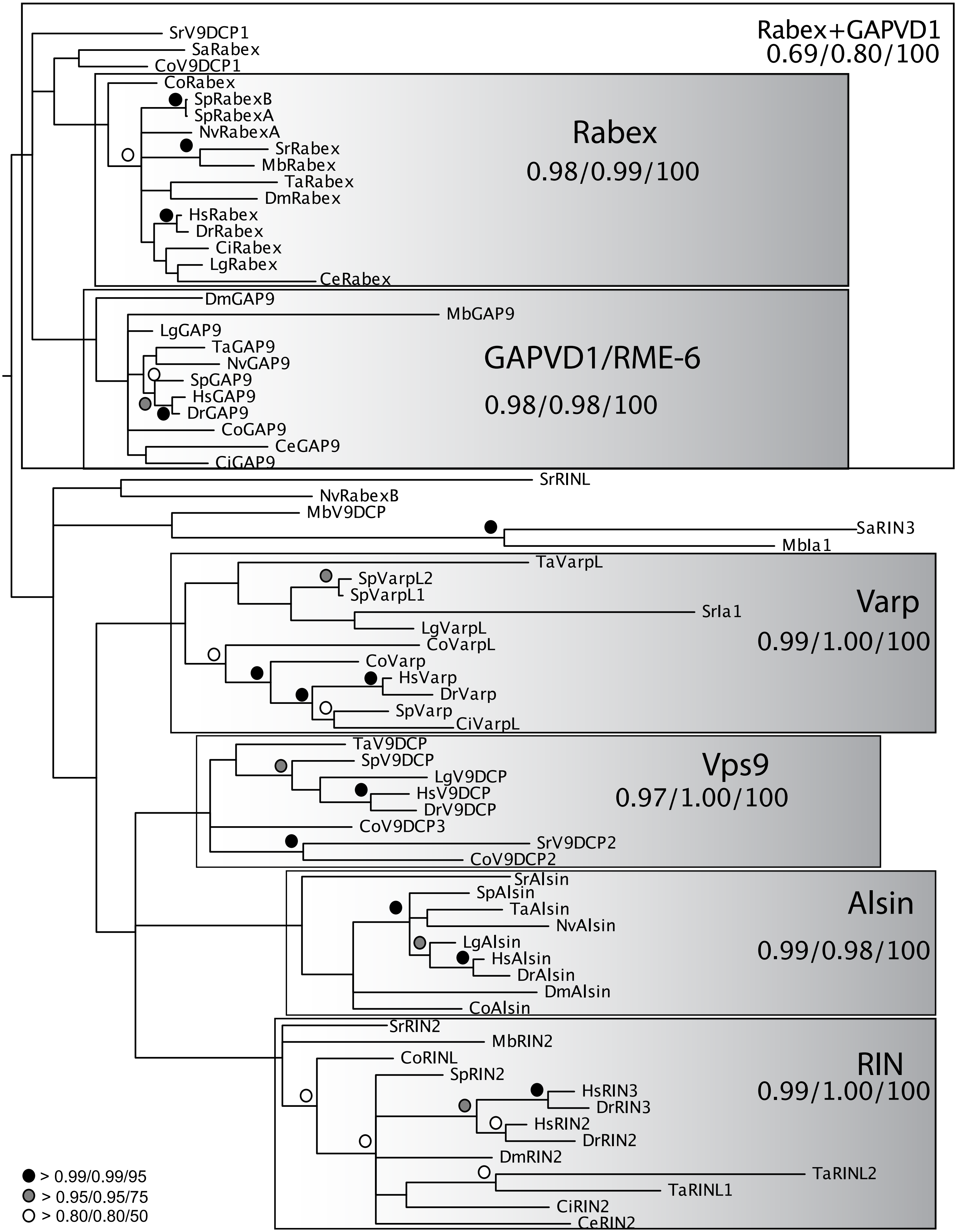


Supplementary Figure 1A

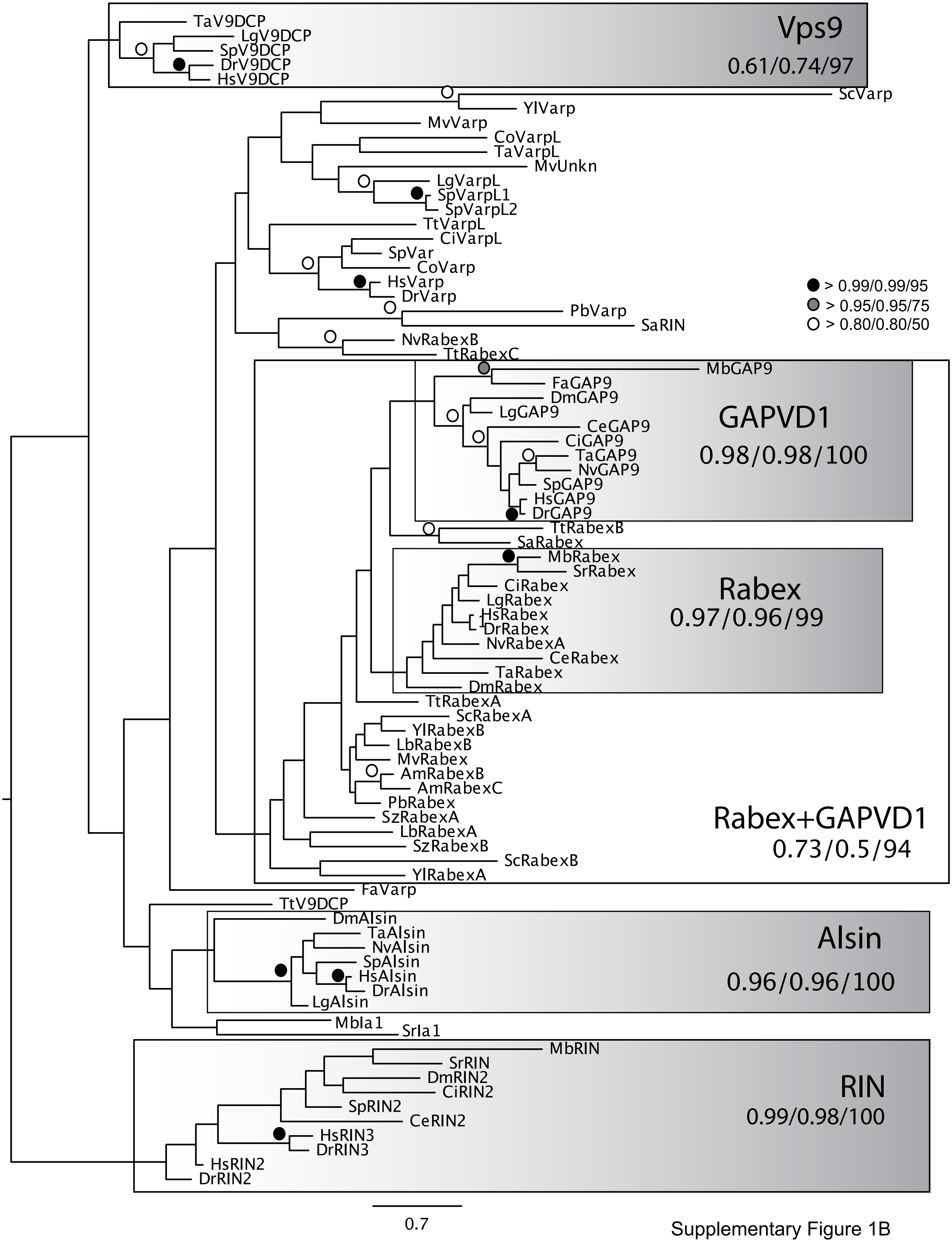


Supplementary Figure 1B

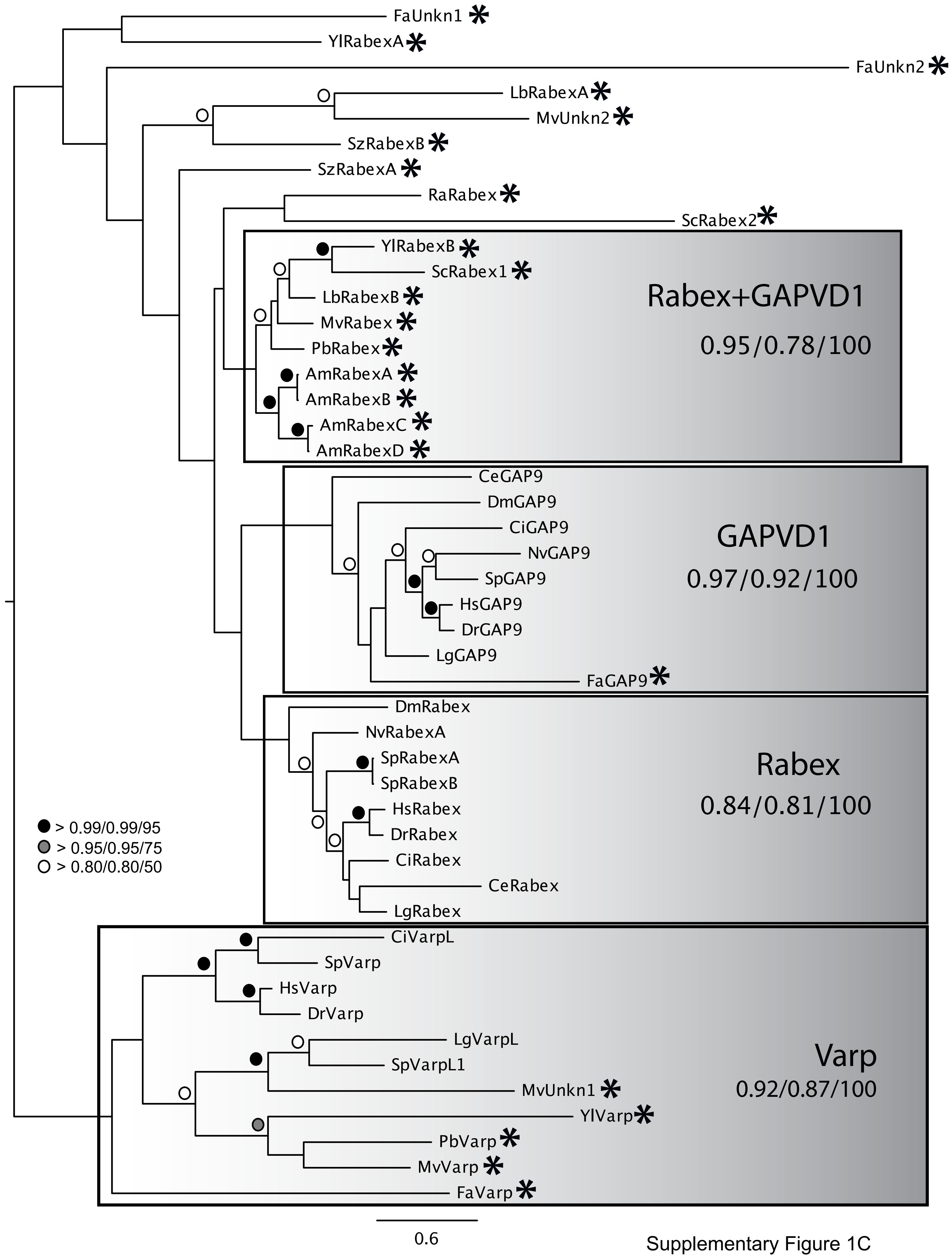


Supplementary Figure 1C

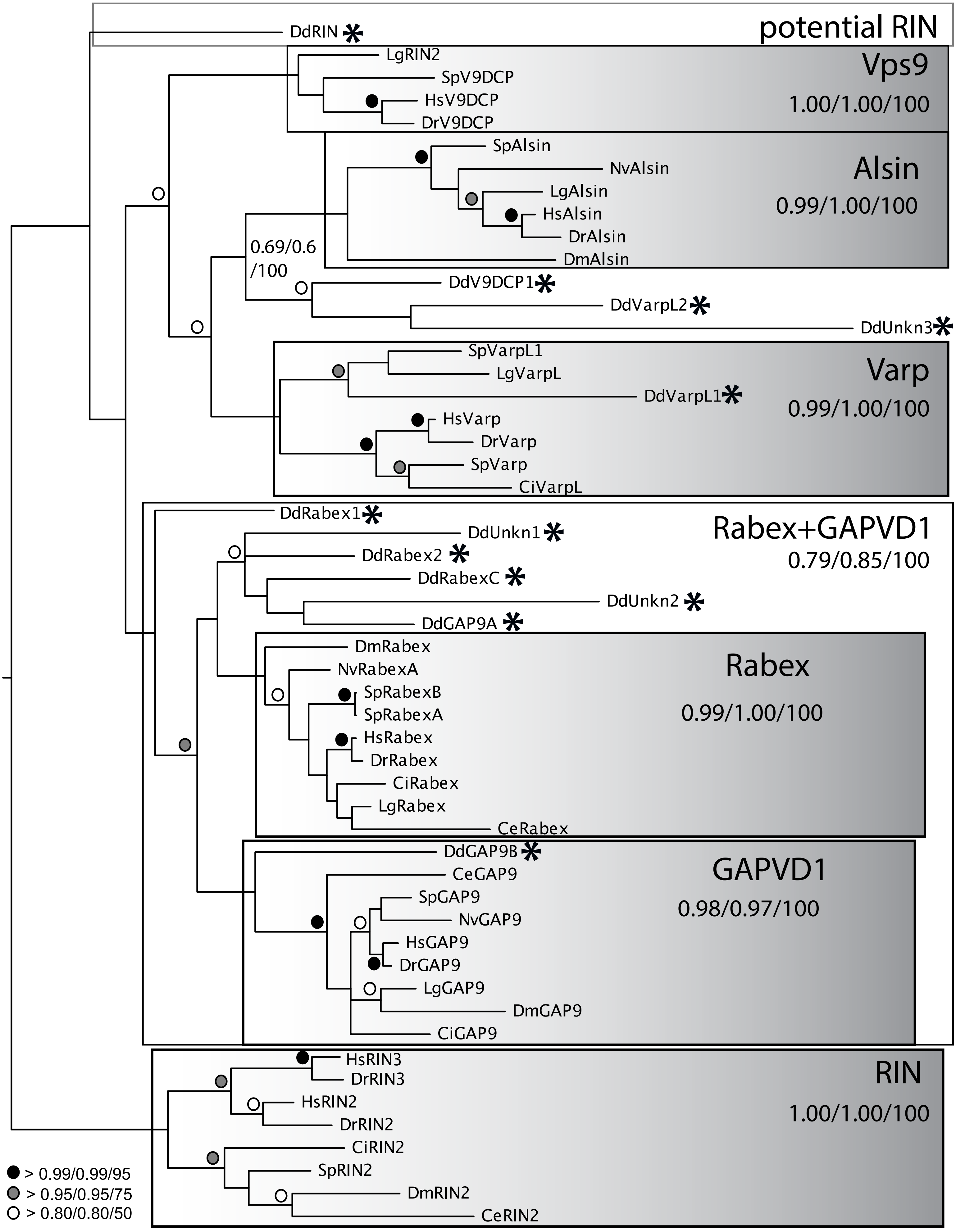


Supplementary Figure 1D

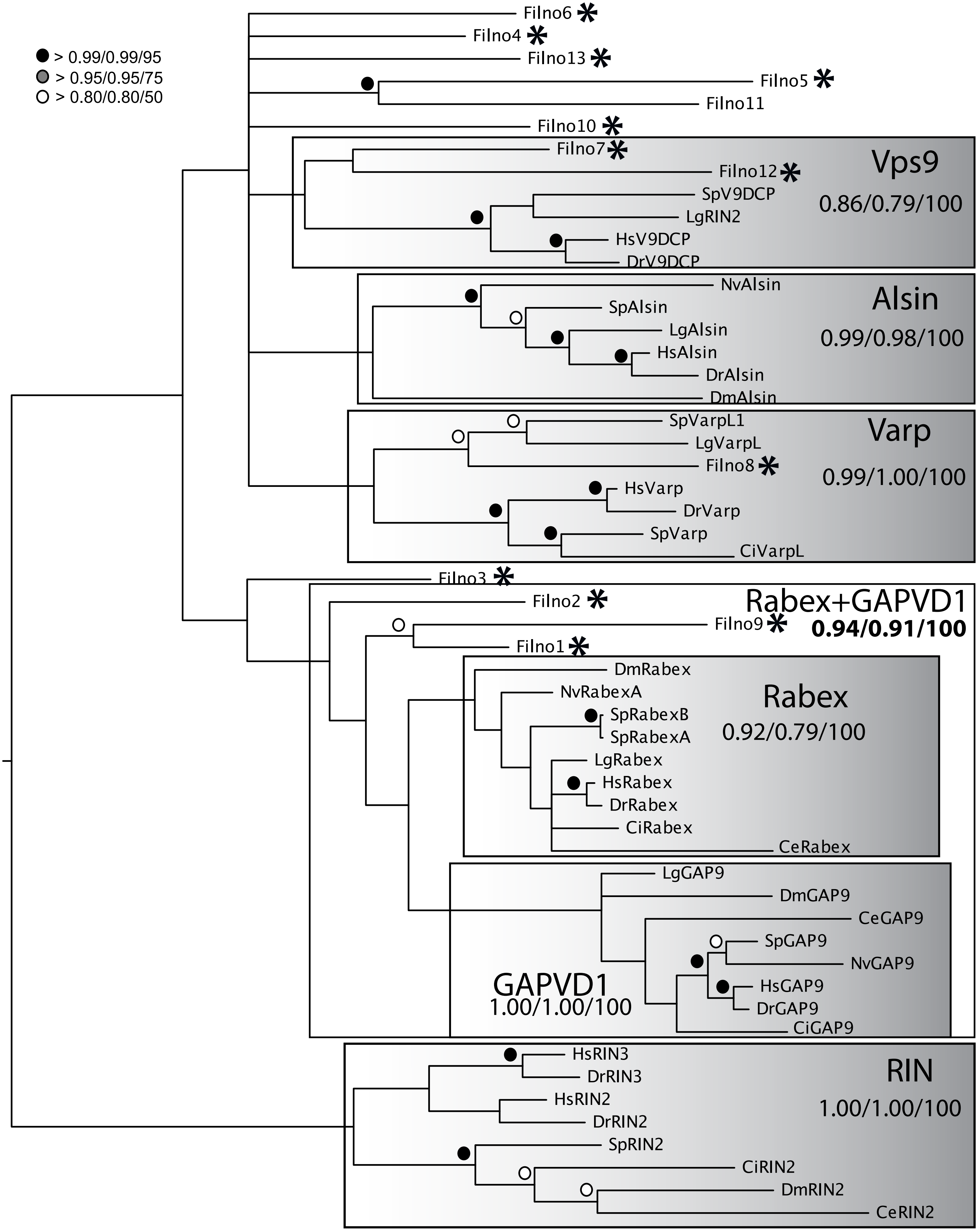


Supplementary Figure 1E

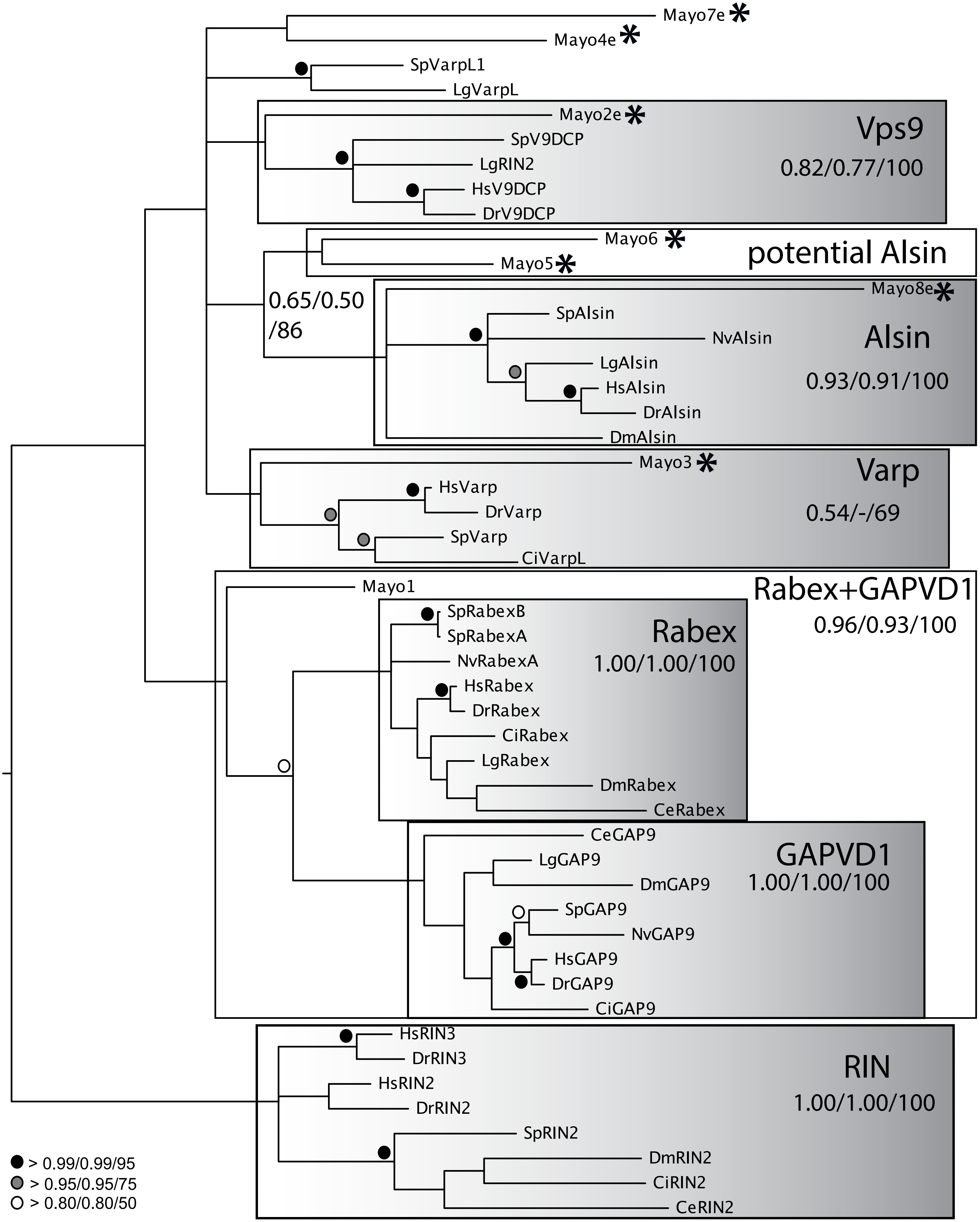


Supplementary Figure 1F

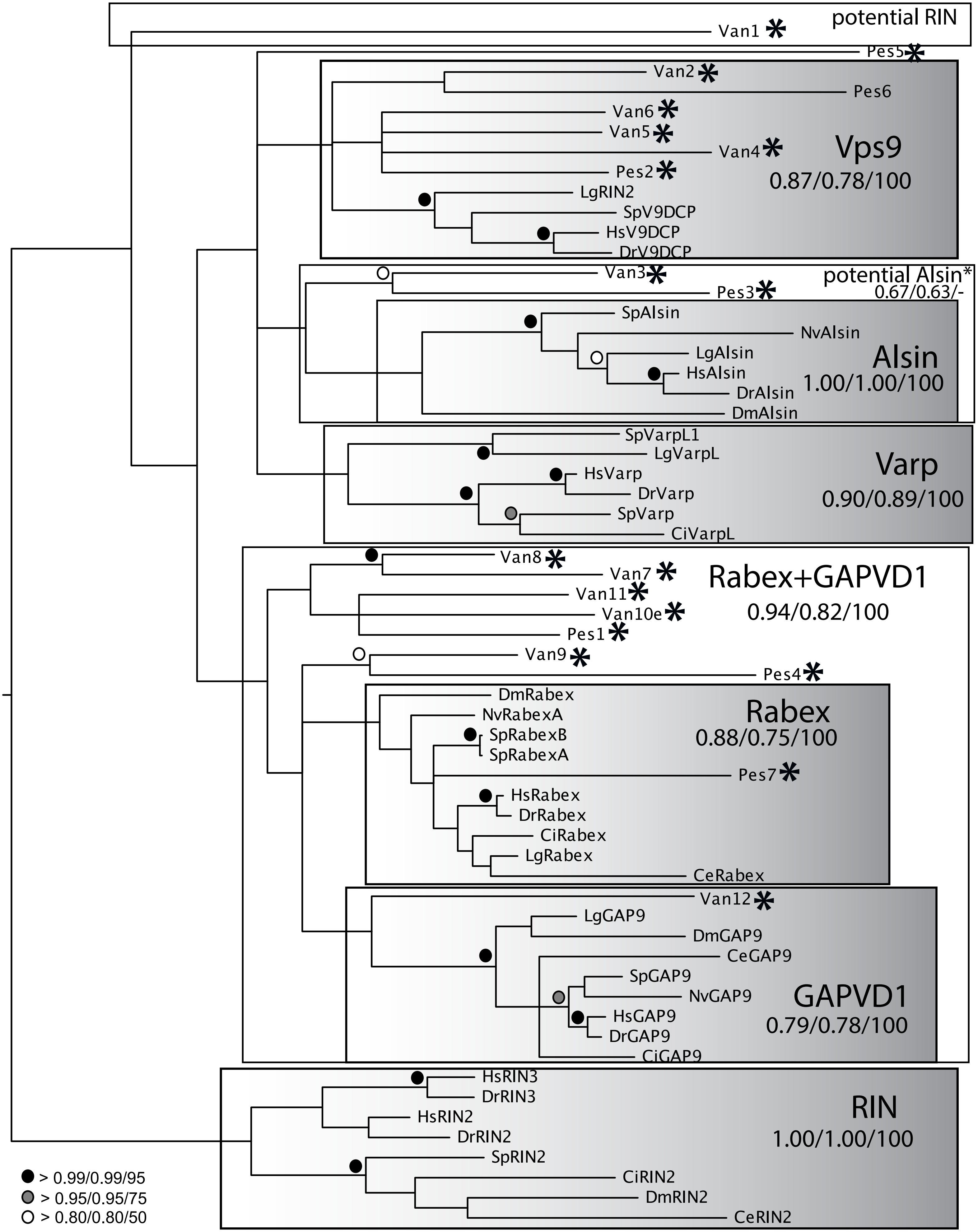


Supplementary Figure 1G

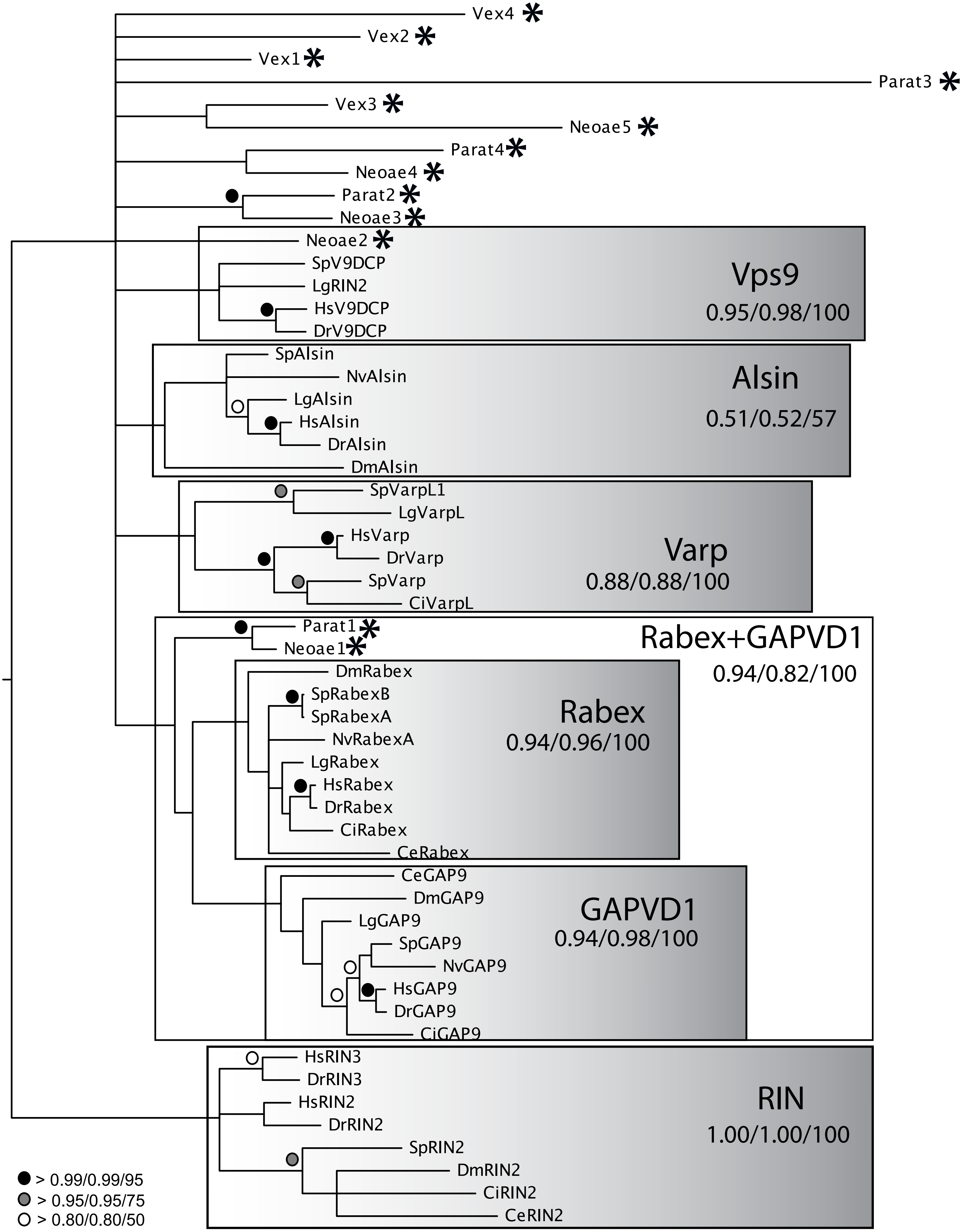


Supplementary Figure 1H

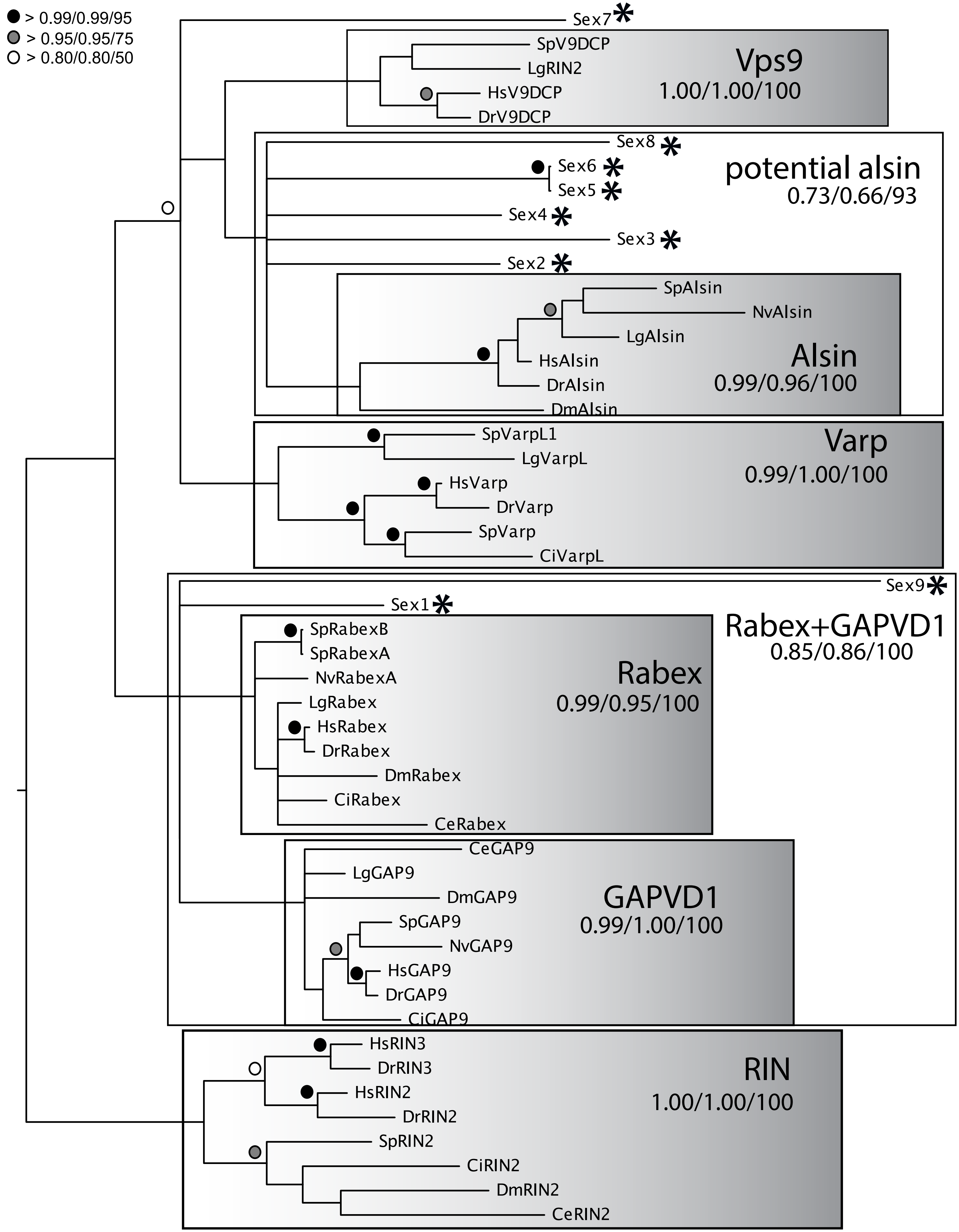


Supplementary Figure 1I

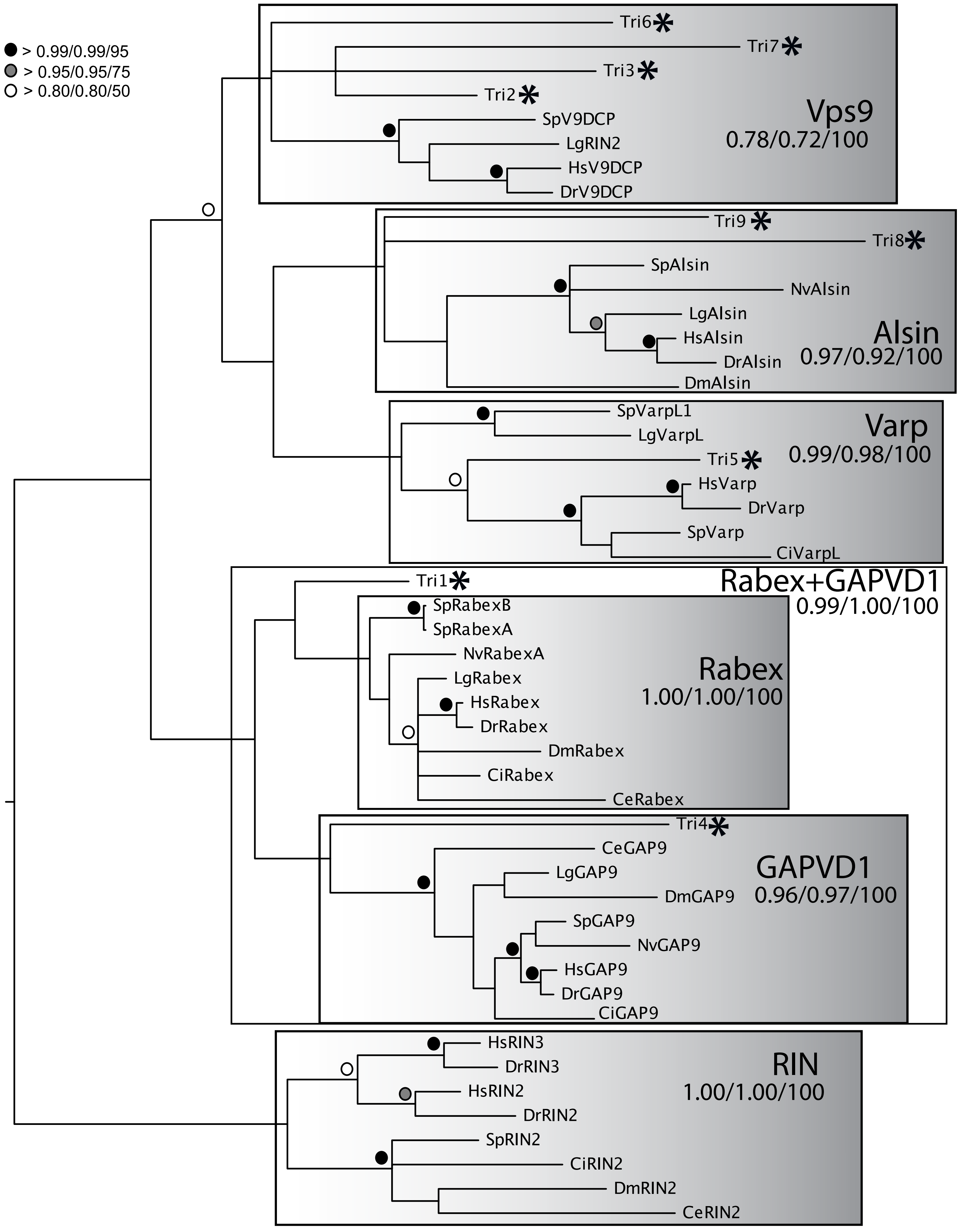


Supplementary Figure 1J

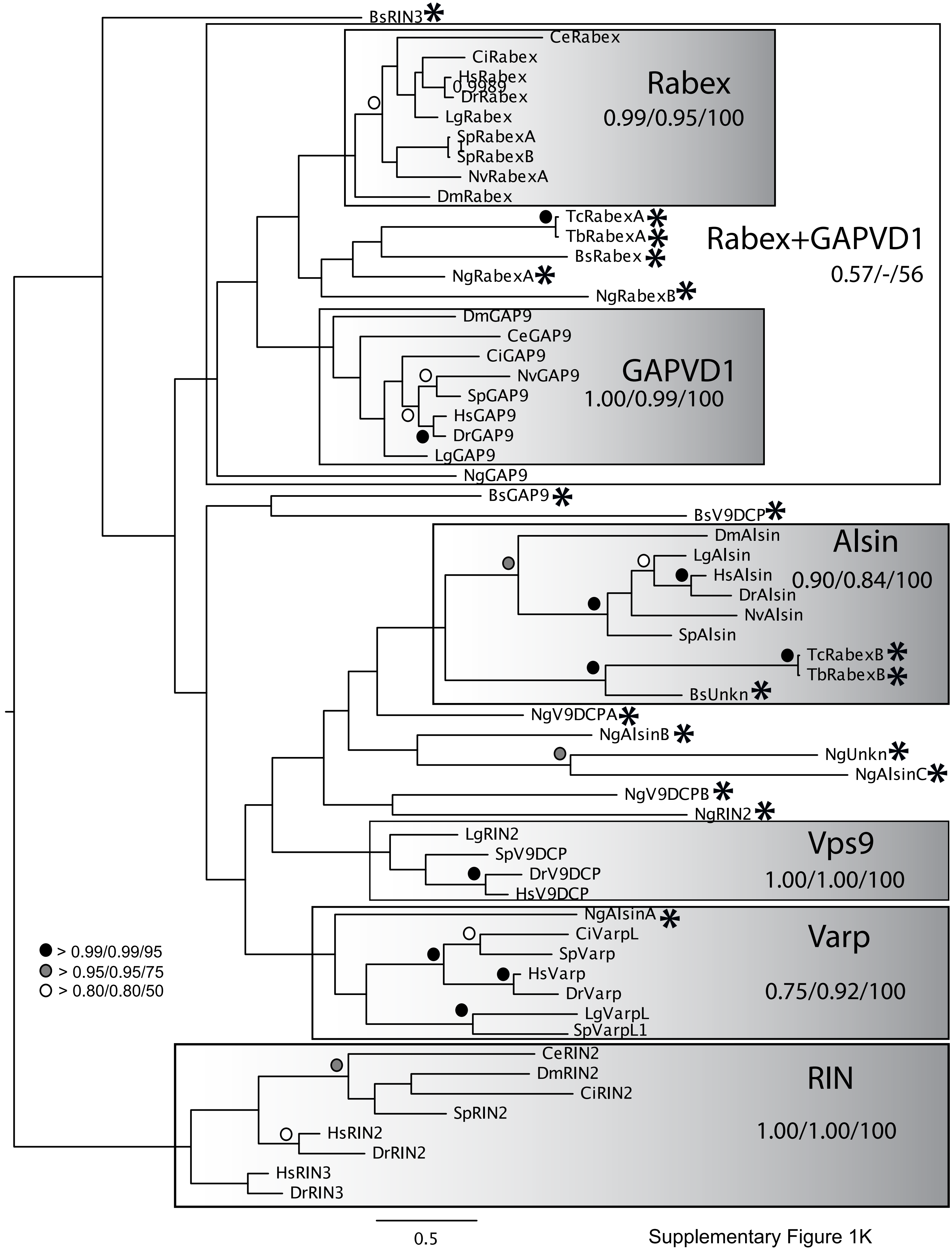


Supplementary Figure 1K

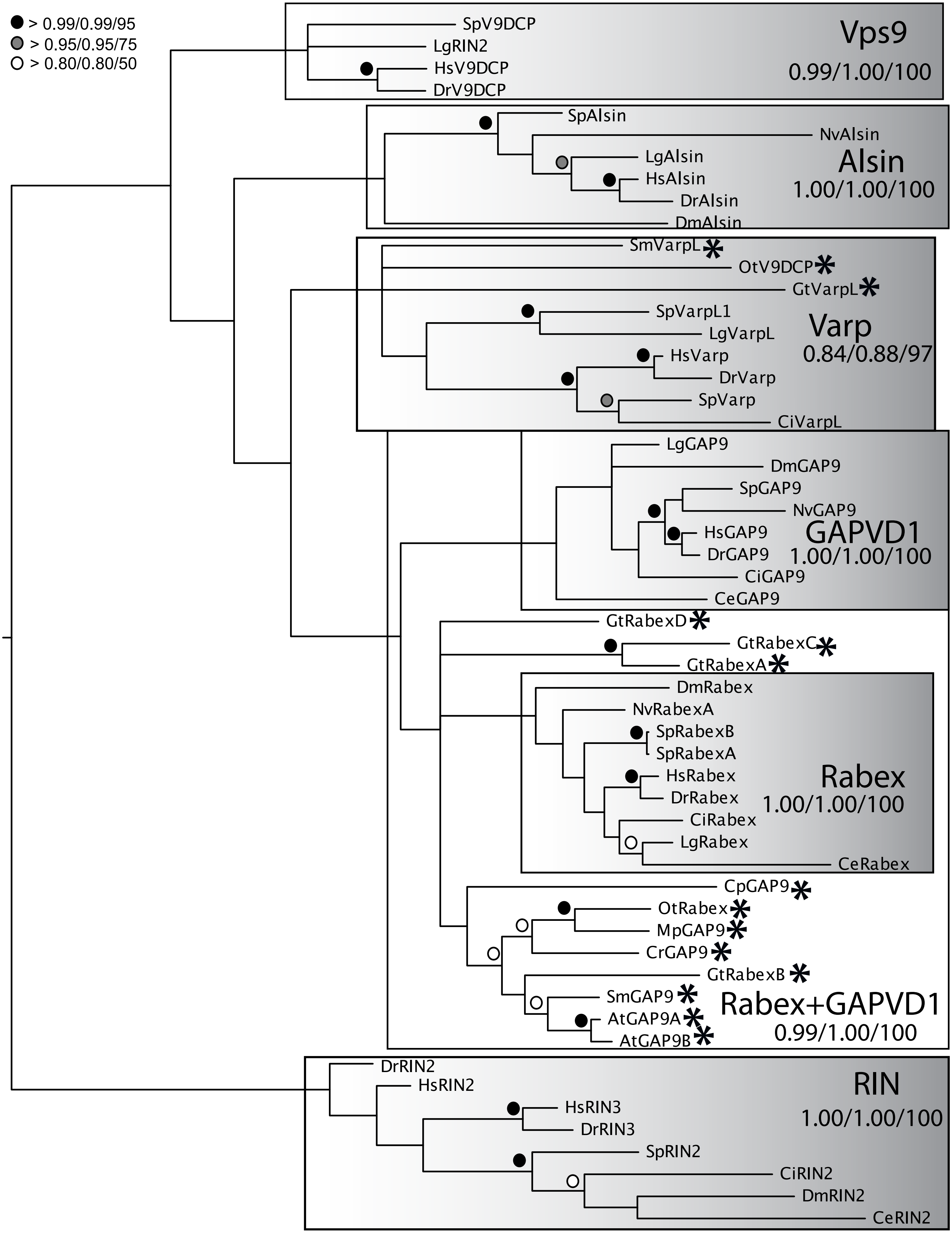


Supplementary Figure 1L

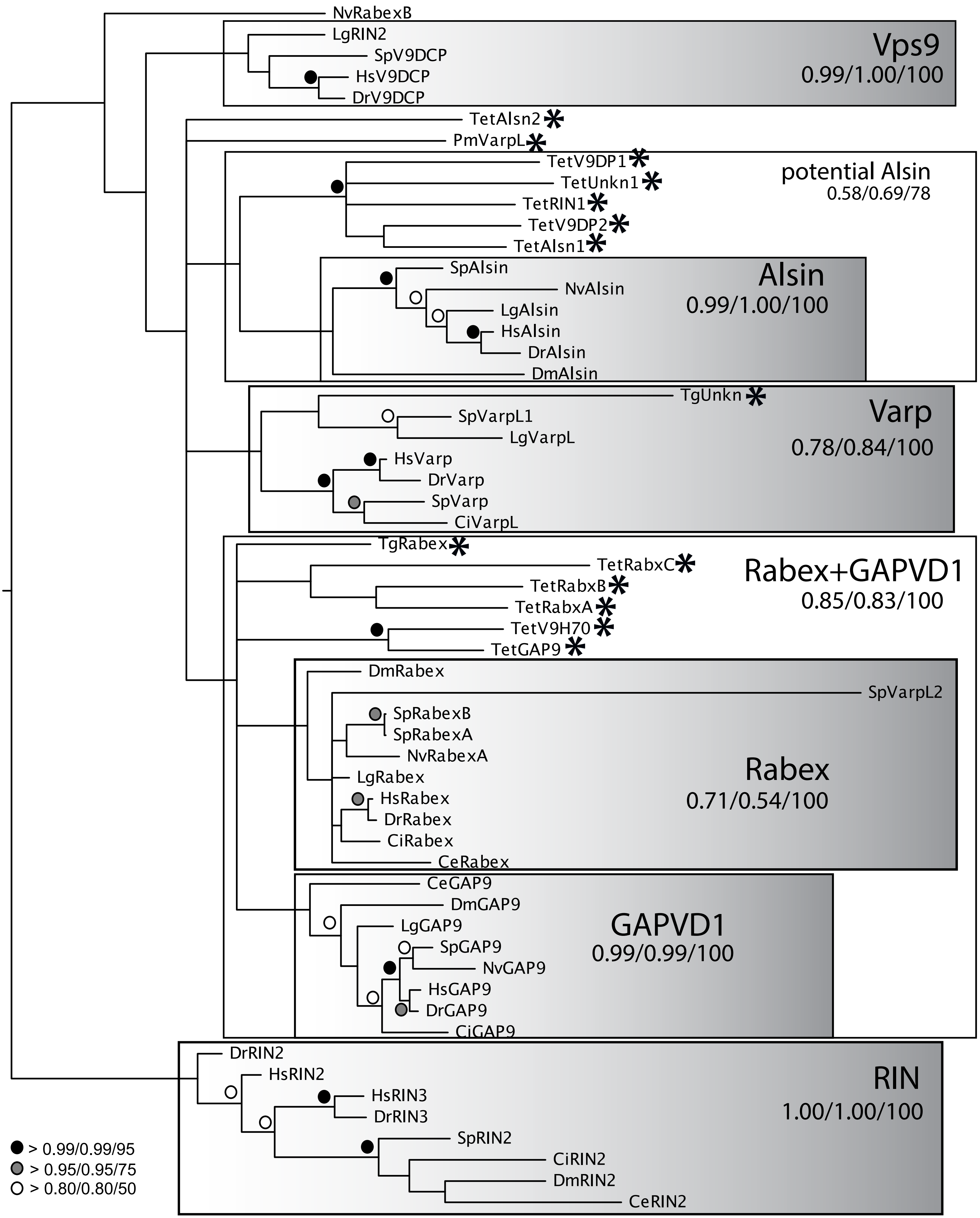


Supplementary Figure 1M

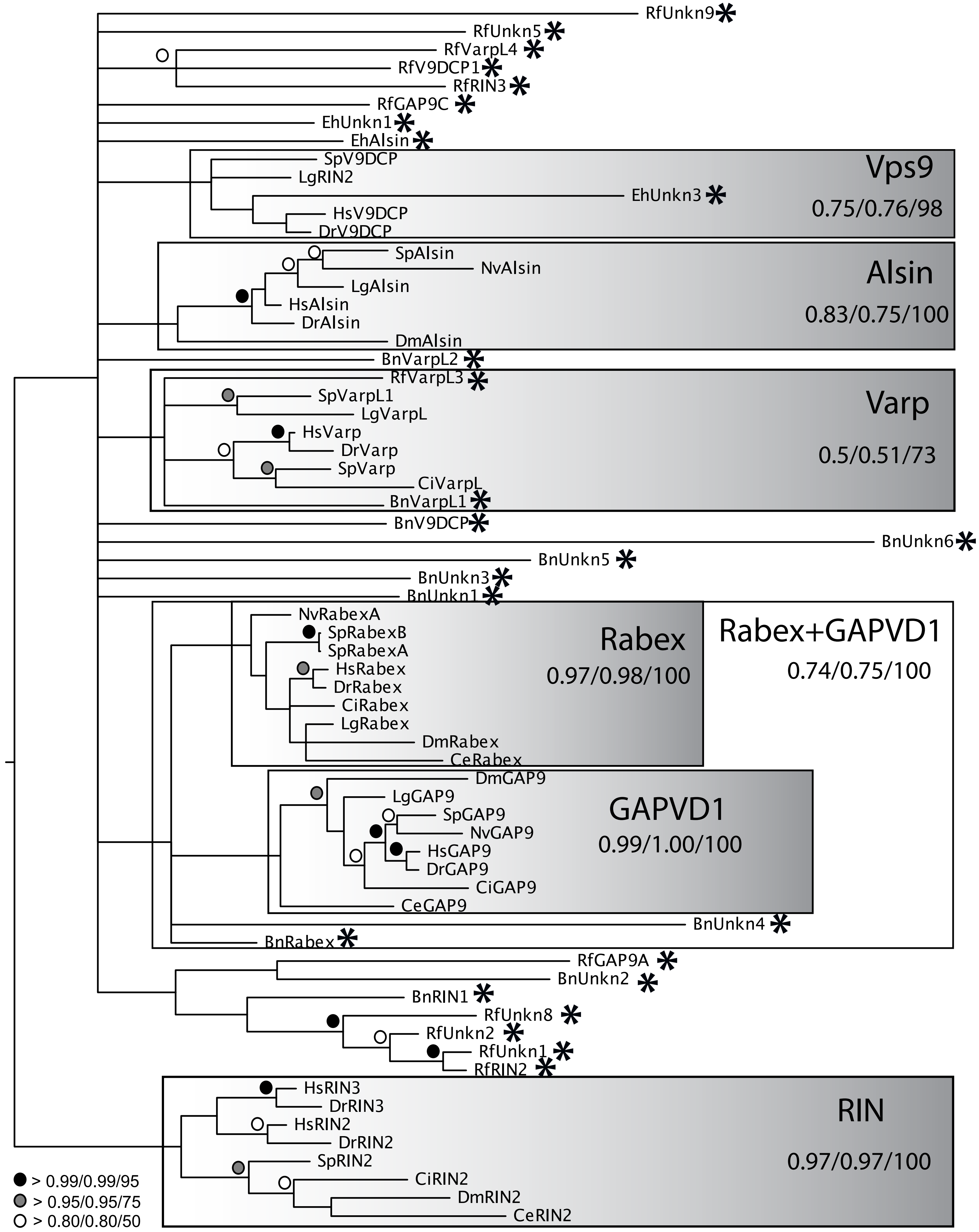


Supplementary Figure 1N

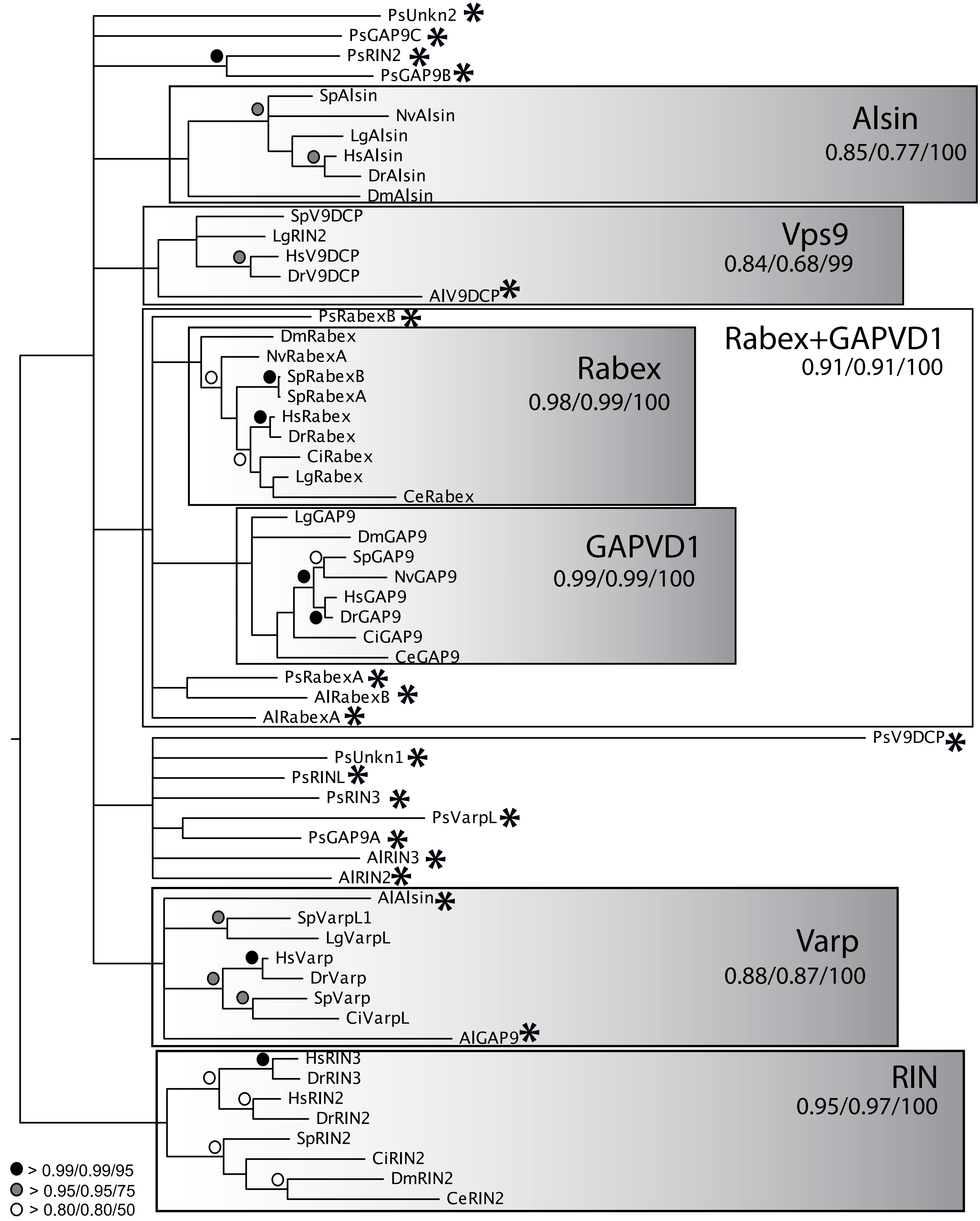


Supplementary Figure 1O

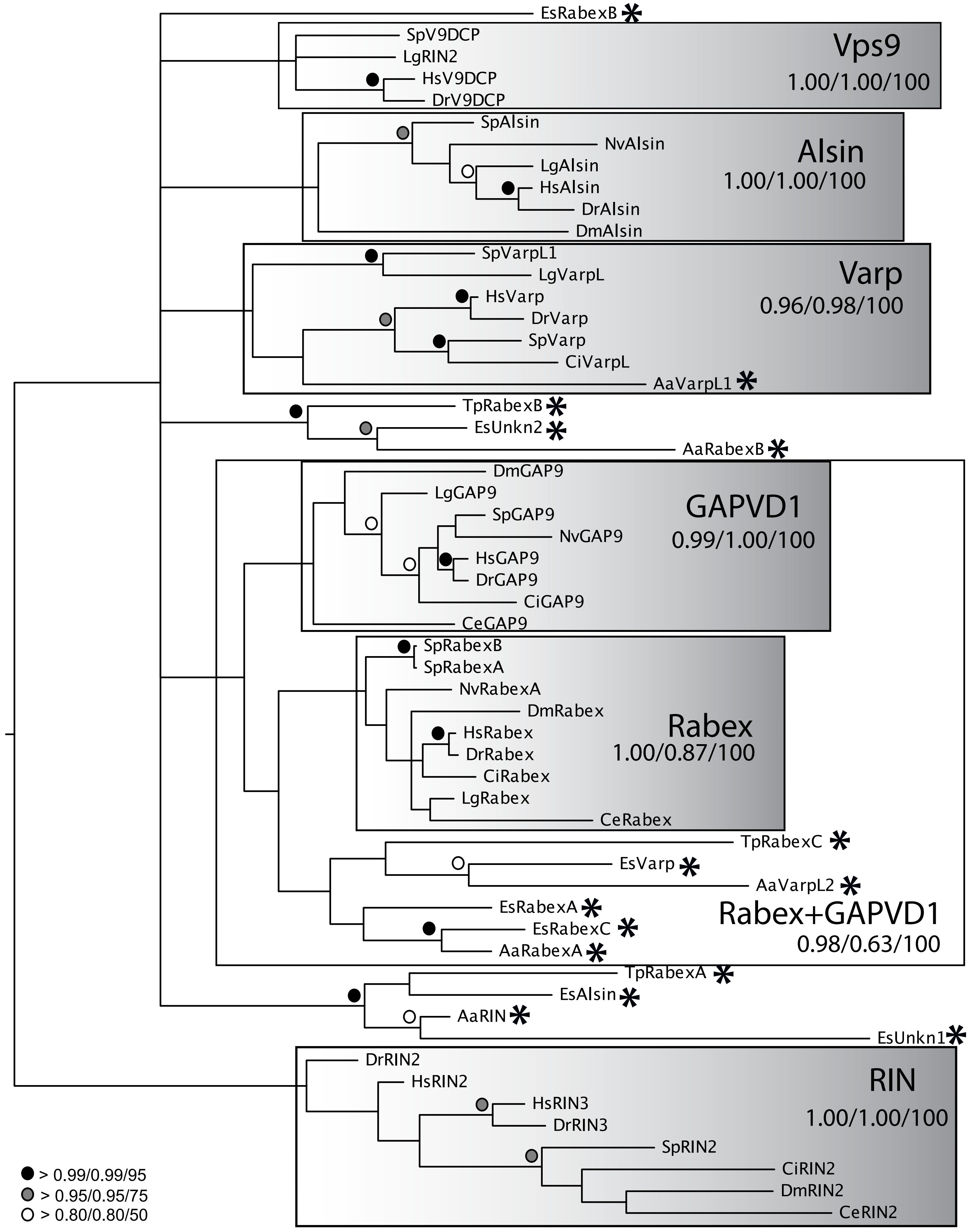


Supplementary Figure 1P

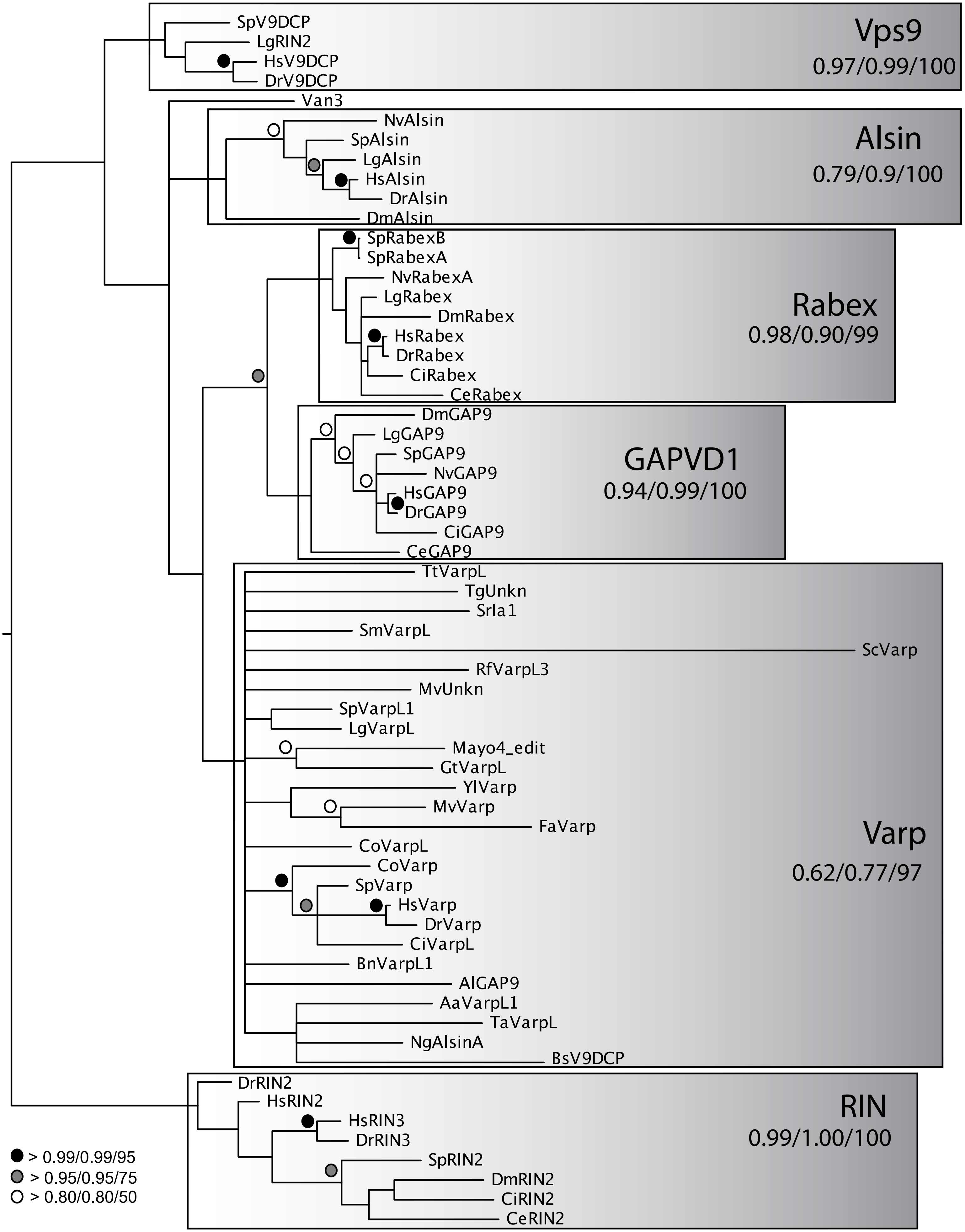


Supplementary Figure 1Q

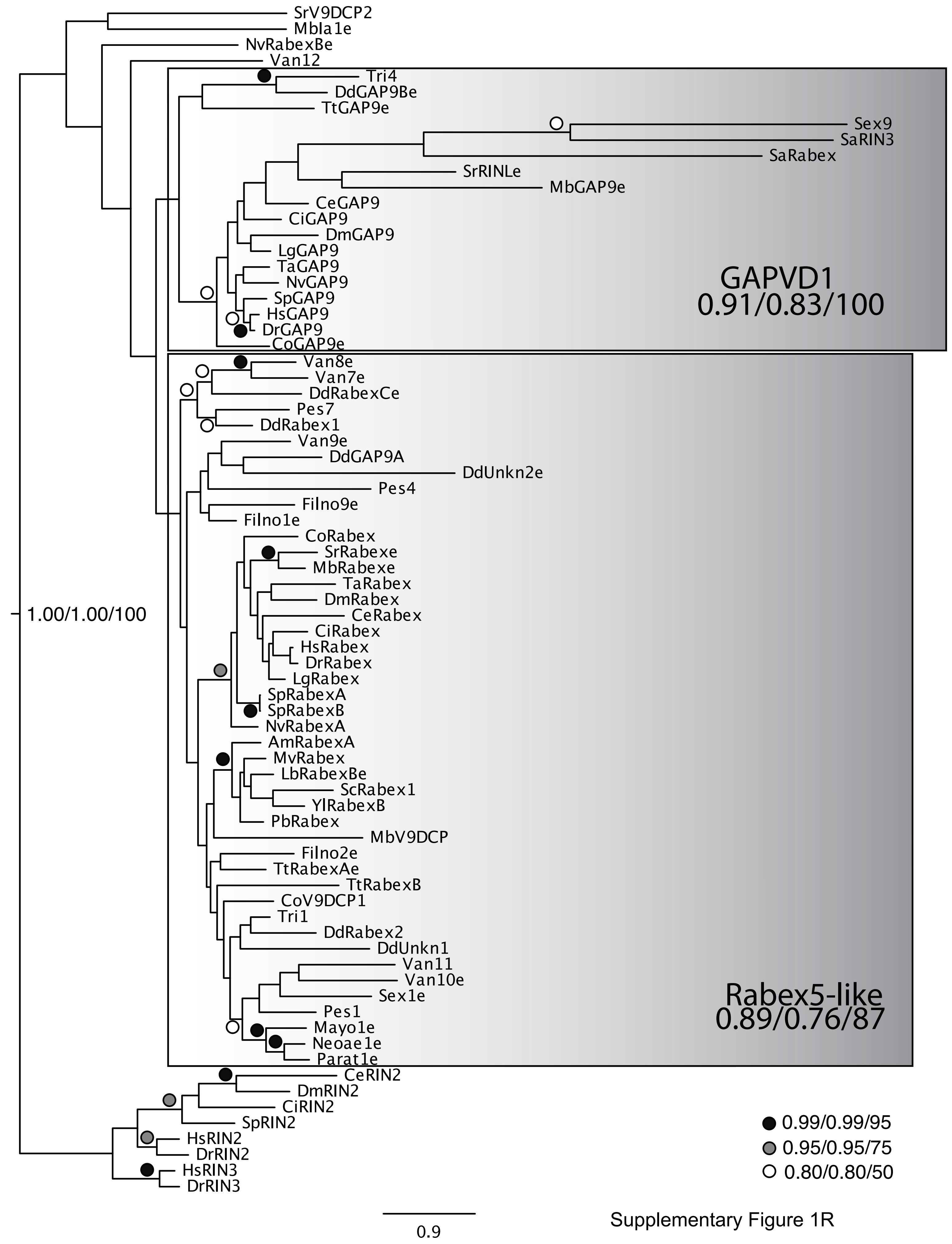


Supplementary Figure 1R

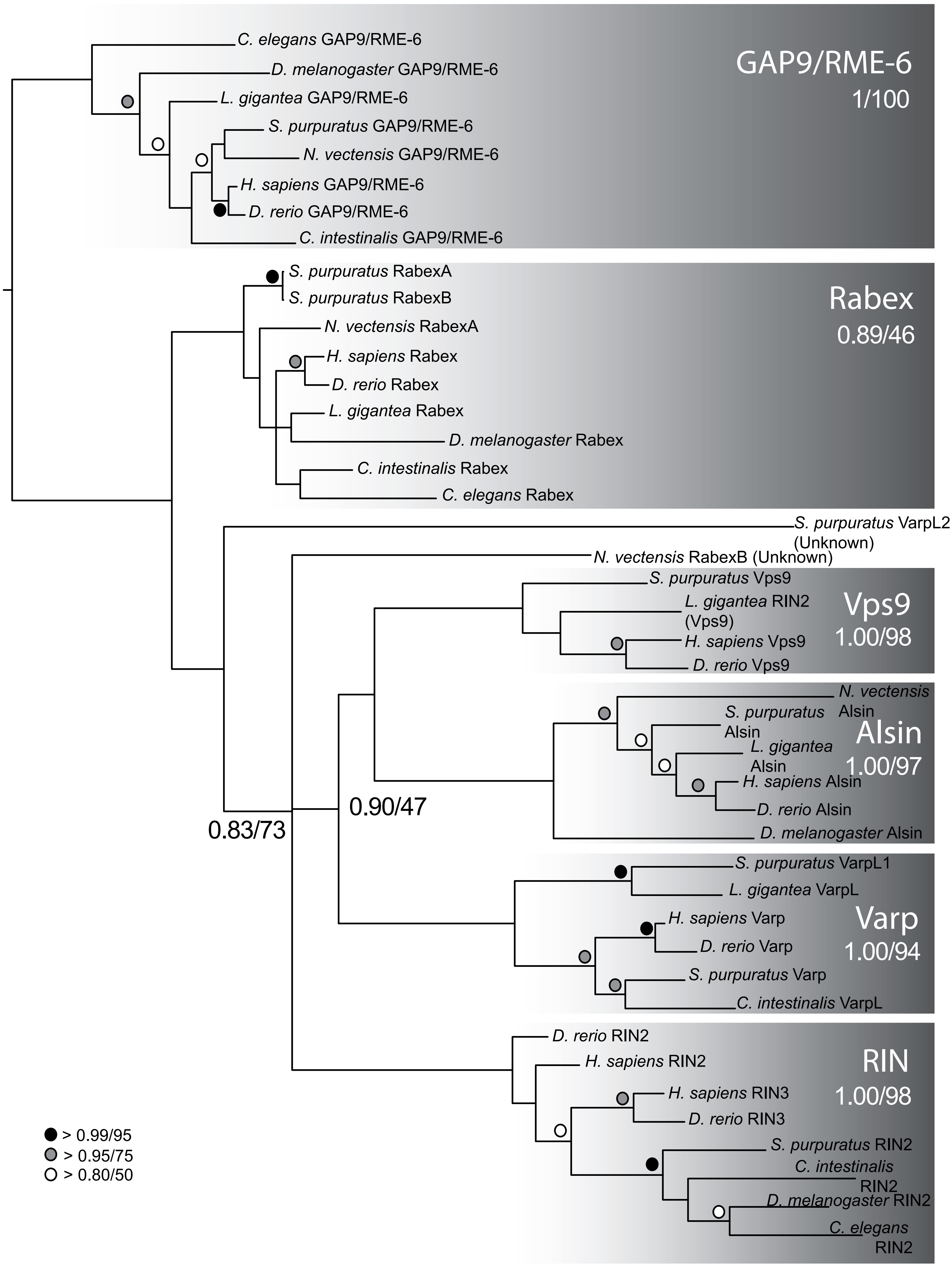


Supplementary Figure 1S

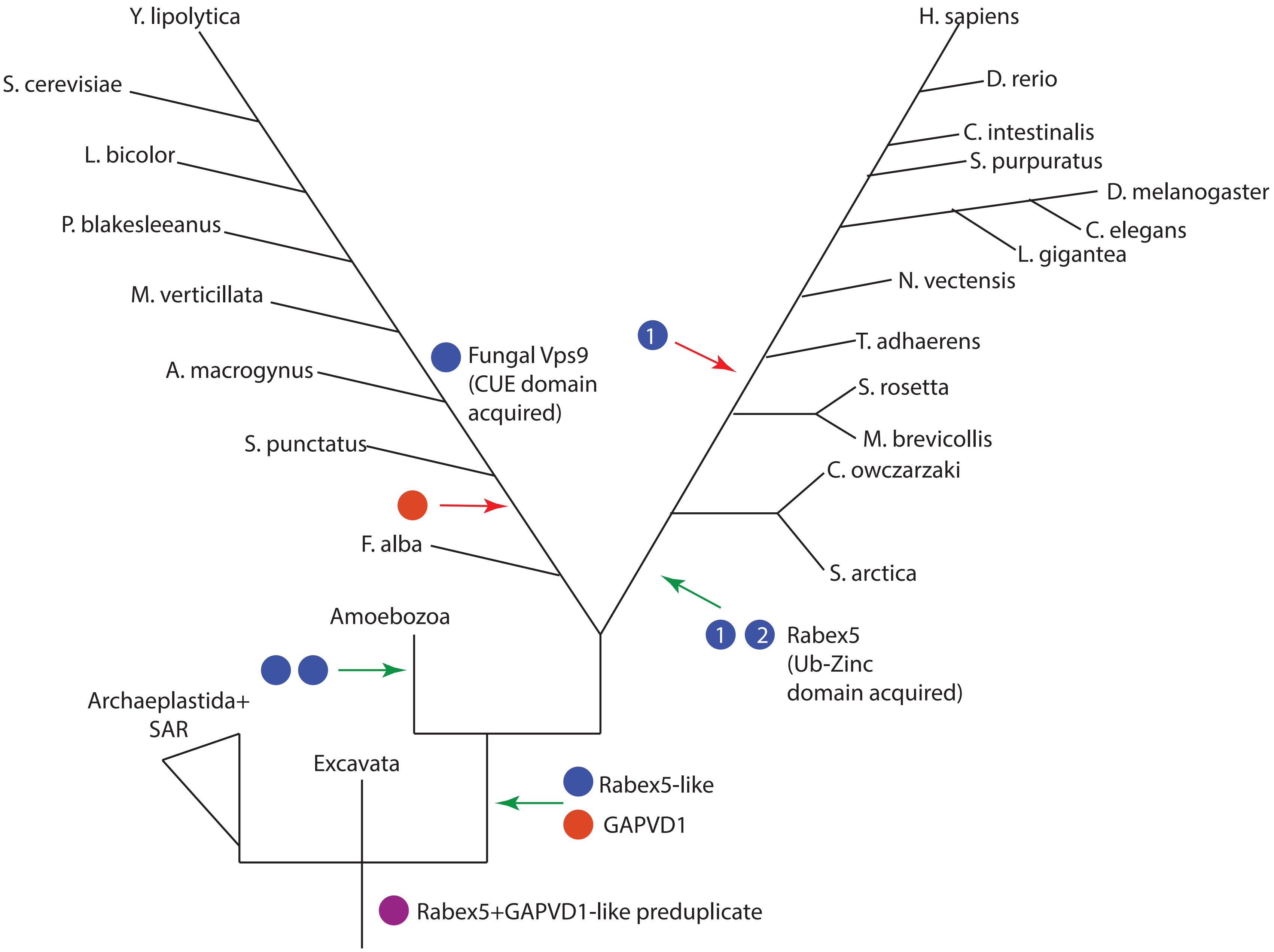


Supplementary Figure 1T

**Supplementary Figure 1. Classification of eukaryotic Vps9 domain-containing proteins.** Sequences from different taxonomic clades are incorporated into the metazoa backbone tree (Supplementary Figure 1S) for classification. In most phylogenies in A-R, node support values for critical nodes are Phylobayes posterior probability/MrBAYES posterior probability/RAxML bootstrap, shown on the Phylobayes topology (unless otherwise noted). For all other nodes, support values are symbolized as inset. Taxon labels are defined in Supplementary Table 1. The phylogenies are arbitrarily rooted on the RIN clade. Clades are marked by shaded boxes, or open boxes in the case of mega-clades (e.g. Rabex+GAPVD1). The following figures were generated to classify (A) holozoan sequences, (B) obazoan sequences, (C) fungal sequences, (D) *Dictyostelium* sequences, (E) *Filamoeba nolandi* sequences, (F) *Mayorella* sp. sequences, (G) *Pessonella* sp. and *Vannella* sp. sequences, (H) *Vexillifera* sp., *Paramoeba atlantica,* and *Neoparamoeba aestuarina* sequences, (I) *Sexangularia* sp. sequences, (J) *Trichosphaerium* sp. sequences, (K) excavate sequences, (L) archaeplastid sequences, (M) alveolate sequences, (N) rhizarian and haptophyte sequences, (O) *Phytophthora sojae* and *Aurantiochytrium limacinum* sequences, and (P) *Ectocarpus siliculosus*, *Thalassiosira pseudonana*, and *Aureococcus anophagefferens* sequences. (Q) shows all eukaryotic Varp sequences within the metazoan backbone tree. Sequences classified as Varp orthologues based on Supplementary Figures 1A-P were added to the metazoan backbone tree. Asterisks denote clade-specific sequences added to the metazoan backbone tree. (R) Phylogeny of all Rabex5 and GAPVD1 in the Amorphea, with metazoan RIN clade sequences as an outgroup. This tree investigates the evolution of the Rabex5-like and GAPVD1 sequences in the Amoebozoa and Opisthokonta. (S) Metazoa backbone tree containing all six Vps9 subfamilies. Supplementary Figures 1B, 1C, 1K, and 1R show the MrBAYES topology for readability, as MrBAYES does not allow polytomies. (T) Rabex5+GAPVD1 protein evolution in the Amorphea. Each circle represents a homologue within the Rabex5+GAPVD1 clade. The ancestral Rabex5+GAPVD1 preduplicate is shown in purple, which duplicated at the base of the Amorphea (red and blue circles). Green arrows denote duplication events, while red arrows denote apparent gene loss events. Numbers within circles are to distinguish gene duplication products, where in the case of the Holozoa, one product acquires a ubiquitin-binding zinc finger and is maintained throughout holozoan evolution, the other duplicate

is lost at the base of multicellular animals (*T. adhaerens*). Domain gain events are indicated. Archaeplastida and SAR clades are collapsed for clarity.

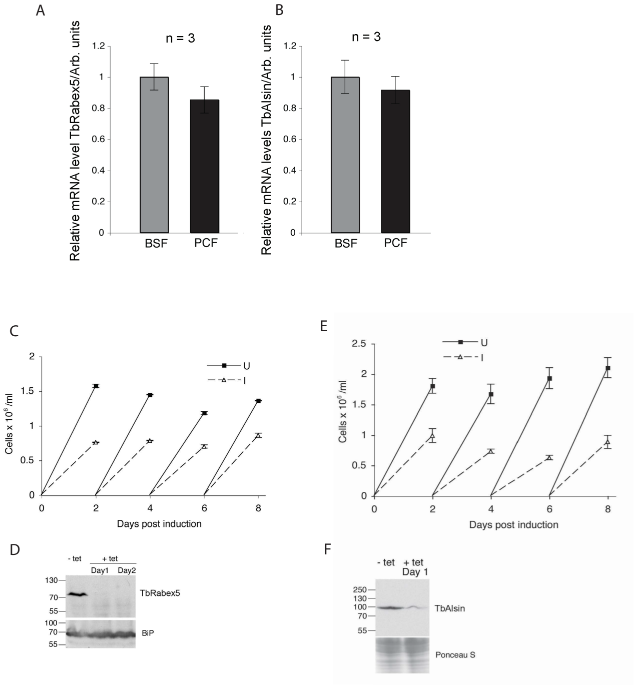


Supplementary Figure 2

**Supplementary Figure 2. TbRabex5 and TbAlsin expression in BSF and PCF cells, and the effects of their depletion on BSF proliferation.** (A,B) The abundance of Vps9 domain guanine nucleotide exchange factor mRNA in BSF and PCF cells was determined by qRT-PCR. Results were normalized to telomerase reverse transcriptase. qRT-PCR was performed on three separate RNA samples each and expression in PCF calibrated against BSF as 1.0. Error bars shows standard error of mean. Grey bars represent mRNA level in BSF, black in PCF cells. (A) TbRabex5 (Tb927.10.10020) and (B) TbAlsin Vps9 (Tb927.3.2430). (C) Growth curve of TbRabex5 depleted BSF parasites. SMB cells were transfected with the RNAi plasmid p2T7 containing TbRabex5 fragment and cells were grown in the presence (open triangles) or absence (closed squares) of 1 µg/ml tetracycline for 8 days. Cells were counted every 48 hours and kept in logarithmic phase by dilution. Error bars represent standard error for duplicate induction. (D) Validation of TbRabex5 knockdown by Western blot using anti-HA antibody in induced and uninduced cells. To confirm equal loading in all lanes, the blot was stripped and re-probed using anti BiP antibody. (E) Growth curve of TbAlsin depleted BSF parasites, experiment performed as

in (C). (F) Validation of TbAlsin knockdown by Western blot of induced and uninduced cells. Ponceau S staining of total protein is shown.

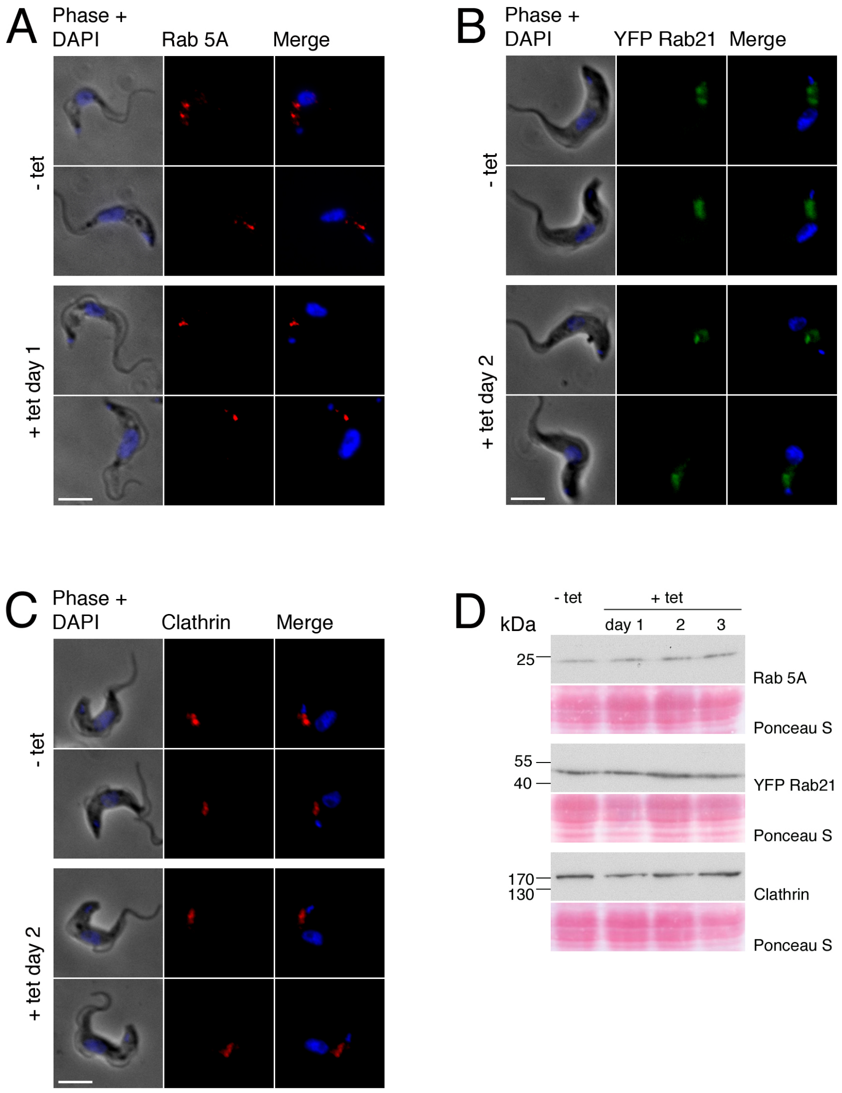


Supplementary Figure 3

**Supplementary Figure 3. Knockdown of TbRabex5 protein does not affect the expression or location of endosomal Rabs.** (A) Immunofluorescence analysis of TbRab5A in TbRabex5 depleted SMB BSF cells. Uninduced (-tet) and induced (+tet) cells were fixed and stained with polyclonal antibodies against TbRab5A. Alexa Fluor 568 labelled antibodies were used to visualize Rab5A and DAPI was used to visualize the nucleus and kinetoplast. Scale bar 5µm. (B) Analysis of TbRab21 in TbRabex5 depleted cells. BSF TbRabex5 knockdown cells were transfected with N-terminal YFP-TbRab21. –tet and +tet cells were fixed and analysed for YFP

native fluorescence while DNA was counterstained using DAPI. (C) IFA of clathrin in TbRabex5 depleted cells. Induced and uninduced RNAi cells were stained with polyclonal antibodies against trypanosome clathrin heavy chain and visualized using Alexa Fluor 568 antibodies. (D) Western Blots and Ponceau S staining showing Rab5A, Rab21, and clathrin heavy chain expression in uninduced and induced cells.

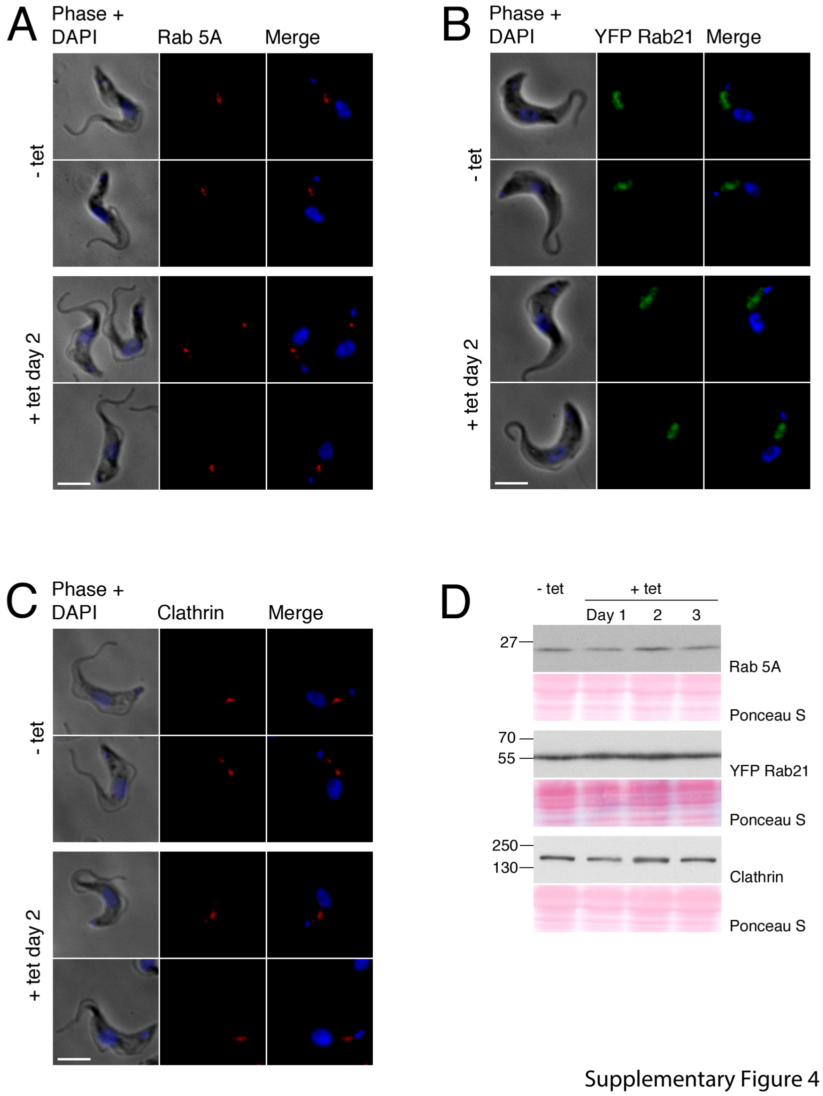


**Supplementary Figure 4. Knockdown of TbAlsin protein does not affect the expression or location of endosomal Rabs.** (A) Immunofluorescence analysis of TbRab5A in TbAlsin depleted SMB BSF cells. Uninduced (-tet) and induced (+tet) cells were fixed and stained with polyclonal antibodies against TbRab5A. Alexa Flour 568 labelled antibodies were used to visualize Rab5A and DAPI was used to visualize nucleus and kinetoplast. Scale bar 5μm. (B) Analysis of TbRab21

in TbAlsin depleted cells. BSF TbAlsin knockdown cells were transfected with N-terminal YFP-TbRab21. -tet and +tet cells were fixed and analysed for YFP native fluorescence while DNA was counterstained using DAPI. (C) IFA of clathrin in TbAlsin depleted cells. Induced and uninduced RNAi cells were stained with polyclonal antibodies against trypanosome clathrin heavy chain and visualized using Alexa Fluor 568 antibodies. (D) Western Blots and Ponceau S staining showing Rab5A, Rab21, and clathrin heavy chain expression in uninduced and induced cells.

**Supplementary Table 1. Classification of eukaryotic Vps9 domain-containing proteins**

**Holozoa**

| **Preliminary annotation** | **Organism** | **Accession** | **Tree name** | **Domain structure (CDD)** | **Actual identity** |
|---|---|---|---|---|---|
| Varp | H. sapiens | NP_115515 | HsVarp | Vps9, Ank, Ank | Varp |
| Rabex5 | H. sapiens | NP_055319 | HsRabex | Ub-binding zinc finger, Vps9 | Rabex |
| GAP/Vps9 | H. sapiens | NP_056450 | HsGAPVD1 | RasGAP, Vps9 | GAPVD1 |
| RIN3 | H. sapiens | NP_079108 | HsRIN3 | SH2, Vps9, RA | RIN3 |
| RIN2 | H. sapiens | NP_061866 | HsRIN2 | SH2, Vps9, RA | RIN2 |
| RIN1 | H. sapiens | NP_004283.2 | HsRIN1 | SH2, Vps9, RA | RIN1 |
| Vps9 DCP | H. sapiens | NP_004904 | HsV9DCP | Vps9 | Vps9 |
| Alsin | H. sapiens | NP_065970 | HsAlsin | RCC, RhoGEF, PH, MORN, Vps9 | Alsin |
| Varp | D. rerio | XP_005163257 | DrVarp | Vps9, Ank, Ank | Varp |
| Rabex5 | D. rerio | XP_005172703 | DrRabex | Vps9 | Rabex |
| GAP/Vps9 | D. rerio | NP_001002420 | DrGAPVD1 | RasGAP, Vps9 | GAPVD1 |
| Vps9 DCP | D. rerio | XP_005169989 | DrV9DCP | Vps9 | Vps9 |
| RIN2 | D. rerio | XP_693977 | DrRIN2 | SH2, Vps9, RA | RIN2 |
| RIN3 | D. rerio | XP_001339622 | DrRIN3 | SH2, Vps9, RA | RIN3 |
| Alsin | D. rerio | XP_685064 | DrAlsin | RCC, RhoGEF, PH, MORN, Vps9 | Alsin |
| VarpL | C. intestinalis | XP_002123303 | CiVarpL | Vps9, Ank, Ank | Varp |
| Rabex5 | C. intestinalis | XP_002130783 | CiRabex | Ub-binding zinc finger, Vps9 | Rabex |
| GAP/Vps9 | C. intestinalis | XP_009859738 | CiGAPVD1 | Vps9 | GAPVD1 |
| RIN2 | C. intestinalis | XP_002126494 | CiRIN2 | Vps9, RA | RIN |
| Varp | S. purpuratus | XP_003727786 | SpVarp | Vps9, Ank, Ank | Varp |
| VarpL | S. purpuratus | XP_795324 | SpVarpL1 | Vps9 | Varp |
| Rabex5 | S. purpuratus | XP_787397 | SpRabexA | Ub-binding zinc finger, Vps9 | Rabex |
| Rabex5 | S. purpuratus | XP_003726521 | SpRabexB | Ub-binding zinc finger, Vps9 | Rabex |
| GAP/Vps9 | S. purpuratus | XP_782192 | SpGAPVD1 | RasGAP, Vps9 | GAPVD1 |
| Vps9 DCP | S. purpuratus | XP_003724404 | SpV9DCP | Vps9 | Vps9 |
| RIN2 | S. purpuratus | XP_003727186 | SpRIN2 | SH2, Vps9, RA | RIN |
| VarpL | S. purpuratus | XP_003725563 | SpVarpL2 | Vps9 (fragment) | Varp? unclear |
| Alsin | S. purpuratus | XP_785995 | SpAlsin | PH, MORN, Vps9 | Alsin |
| Rabex5 | D. melanogaster | FBpp0072557 | DmRabex | Ub-binding zinc finger, Vps9 | Rabex |
| GAP/Vps9 | D. melanogaster | FBpp0073304 | DmGAPVD1 | RasGAP, Vps9 | GAPVD1 |

| | | | | | |
|---|---|---|---|---|---|
| Alsin | D. melanogaster | FBpp0078116 | DmAlsin | RCC, PH, MORN, Vps9 | Alsin |
| RIN2 | D. melanogaster | FBpp0111602 | DmRIN2 | Vps9, RA | RIN |
| Rabex5 | C. elegans | Y39A1A.5 | CeRabex | Ub-binding zinc finger, Vps9 | Rabex |
| GAP/Vps9 | C. elegans | F49E7.1a | CeGAPVD1 | RasGAP, Vps9 | GAPVD1 |
| RIN2 | C. elegans | C48G7.3e | CeRIN2 | Vps9, RA | RIN |
| VarpL | L. gigantea | 237017 | LgVarpL | Vps9 | Varp |
| GAP/Vps9 | L. gigantea | 130207 | LgGAPVD1 | Vps9 | GAPVD1 |
| Rabex5 | L. gigantea | 140110 | LgRabex | Ub-binding zinc finger, Vps9 | Rabex |
| RIN2=> V9DCP | L. gigantea | 233615 | LgRIN2/V9DCP | Vps9 | V9DCP |
| Alsin | L. gigantea | 145057 | LgAlsin | PH, MORN, Vps9 | Alsin |
| GAP/Vps9 | N. vectensis | 183502 | NvGAPVD1 | Vps9 | GAPVD1 |
| Rabex5 | N. vectensis | 183590 | NvRabexA | Ub-binding zinc finger, Vps9 | Rabex |
| Rabex5 | N. vectensis | 217703 | NvRabexB | Vps9 | unclear |
| Alsin | N. vectensis | 164111 | NvAlsin | MORN, Vps9 | Alsin |
| VarpL | T. adhaerens | 59078 | TaVarpL | Vps9 | Varp |
| Vps9 DCP | T. adhaerens | 55790 | TaV9DCP | Vps9 | Vps9 |
| GAP/Vps9 | T. adhaerens | 20735 | TaGAPVD1 | Vps9 | GAPVD1 |
| Rabex5 | T. adhaerens | 21521 | TaRabex | Vps9 | Rabex |
| Alsin | T. adhaerens | 60444 | TaAlsin | RCC, RhoGEF, MORN, Vps9 | Alsin |
| RINL | T. adhaerens | 64219 | TaRINL1 | SH2, Vps9 | RIN |
| RINL | T. adhaerens | 62061 | TaRINL2 | SH2, Vps9 | RIN |
| Rabex5 | M. brevicollis | MONBRDRAFT_34581 | MbRabex | Epimerase/aldehyde reductase, Vps9 | Rabex |
| Ia1 | M. brevicollis | MONBRDRAFT_33028 | MbIa1 | Vps9 | unclear |
| RIN2 | M. brevicollis | MONBRDRAFT_23795 | MbRIN2 | SH2, Vps9, RA | RIN |
| GAP/Vps9 | M. brevicollis | MONBRDRAFT_28498 | MbGAPVD1 | Vps9 | GAPVD1 |
| V9DCP | M. brevicollis | MONBRDRAFT_30406 | MbV9DCP | Vps9 | Rabex+GAPVD1 |
| Varp | S. rosetta | PTSG_12563 | SrVarp | Vps9, Ank, Ank | Varp |
| RIN2 | S. rosetta | PTSG_09467 | SrRIN2 | SH2, Vps9 | RIN |
| Rabex5 | S. rosetta | PTSG_01402 | SrRabex | Vps9 | Rabex |
| V9DCP | S. rosetta | PTSG_04215 | SrV9DCP1 | Vps9 | Rabex+GAPVD1** |
| RINL | S. rosetta | PTSG_06144 | SrRINL | SH2, SH2, Vps9 | Rabex+GAPVD1 |
| Alsin | S. rosetta | PTSG_07928 | SrAlsin | RCC, PH, MORN, Vps9 | Alsin |
| V9DCP | S. rosetta | PTSG_11691 | SrV9DCP2 | Vps9 | unclear |

| | | | | | |
|---|---|---|---|---|---|
| Ia1 | S. rosetta | PTSG_02578 | SrIa1 | Vps9 | Varp? unclear |
| Varp | C. owczarzaki | CAOG_02852 | CoVarp | Vps9, Ank, Ank | Varp |
| VarpL | C. owczarzaki | CAOG_07170 | CoVarpL | Vps9 | Varp |
| V9DCP | C. owczarzaki | CAOG_04920 | CoV9DCP1 | Vps9 | Rabex+GAPVD1 |
| Rabex5 | C. owczarzaki | CAOG_06854 | CoRabex | Ub-binding zinc finger, Vps9 | Rabex+GAPVD1 |
| GAP/Vps9 | C. owczarzaki | CAOG_01708 | CoGAPVD1 | RasGAP, Vps9 | GAPVD1 |
| RINL | C. owczarzaki | CAOG_02871 | CoRINL | Vps9, RA | RIN |
| Alsin | C. owczarzaki | CAOG_01016 | CoAlsin | RCC, PH, MORN, Vps9 | Alsin |
| V9DCP | C. owczarzaki | CAOG_04207 | CoV9DCP2 | Vps9 | Vps9 |
| V9DCP | C. owczarzaki | CAOG_03201 | CoV9DCP3 | Vps9 | Vps9 |
| Rabex5 | S. arctica | SARC_00646 | SaRabex | Vps9 | Rabex+GAPVD1 |
| RIN3 | S. arctica | SARC_13584 | SaRIN3 | Vps9 | Rabex+GAPVD1 |

** (in holozoa tree, Supplementary Figure 1A)

**Fungi**

| **Preliminary annotation** | **Organism** | **Accession** | **Tree name** | **Domain structure (CDD)** | **Actual identity** |
|---|---|---|---|---|---|
| Varp | F. alba | H696_05968 | FaVarp | Vps9, Ank | Varp |
| GAP/Vps9 | F. alba | H696_00539 | FaGAPVD1 | RasGAP, Vps9 | GAPVD1? unclear |
| Unknown | F. alba | H696_06297 | FaUnkn1 | Vps9 | Unknown |
| Unknown | F. alba | H696_04226 | FaUnkn2 | Vps9 | Unknown |
| Rabex5 | R. allomycis | 3581 | RaRabex | Vps9 | Unknown |
| Rabex5 | Y. lipolytica | 70744 | YlRabexA | RhoGEF, Vps9 | Unknown |
| Rabex5 | Y. lipolytica | 69486 | YlRabexB | Vps9, CUE domain | Fungal Rabex-GAPVD1-like clade |
| Varp | Y. lipolytica | 68897 | YlVarp | Vps9, Ank, Ank | Varp clade |
| Rabex5 | S. cerevisiae | YML097C | ScRabex1/A | Vps9, CUE domain | Fungal Rabex-GAPVD1-like clade |
| Varp | S. cerevisiae | YML002W | ScVarp | Vps9, Ank, Ank | Varp** |
| Rabex5 | S. cerevisiae | YPL070W | ScRabex2/B | Vps9 | Unknown |
| Rabex5 | L. bicolor | 303581 | LbRabexA | Vps9 | Unknown |
| Rabex5 | L. bicolor | 305240 | LbRabexB | Vps9, CUE domain | Fungal Rabex-GAPVD1-like clade |
| Varp | P. blakesleeanus | 71663 | PbVarp | Vps9, Ank, Ank | Varp |
| Rabex5 | P. blakesleeanus | 181154 | PbRabex | Vps9, CUE domain | Fungal Rabex-GAPVD1-like clade |

| | | | | | |
|---|---|---|---|---|---|
| Varp | M. verticillata | MVEG_04782 | MvVarp | Vps9, Ank, Ank, PX | Varp |
| Rabex5 | M. verticillata | MVEG_04758 | MvRabex | Vps9, Ubiquitin domain superfamily | Fungal Rabex-GAPVD1-like clade |
| Unknown | M. verticillata | MVEG_00996 | MvUnkn1 | Vps9 | Varp |
| Unknown | M. verticillata | MVEG_09186 | MvUnkn2 | Vps9 | Unknown |
| Rabex5 | A. macrogynus | AMAG_03935 | AmRabexA | Vps9 | Fungal Rabex-GAPVD1-like clade |
| Rabex5 | A. macrogynus | AMAG_17146 | AmRabexB | Vps9 | Fungal Rabex-GAPVD1-like clade |
| Rabex5 | A. macrogynus | AMAG_17498 | AmRabexC | Vps9 | Fungal Rabex-GAPVD1-like clade |
| Rabex5 | A. macrogynus | AMAG_12631 | AmRabexD | Vps9 | Fungal Rabex-GAPVD1-like clade |
| Rabex5 | S. punctatus | SPPG_02656 | SzRabexA | Vps9 | Unknown |
| Rabex5 | S. punctatus | SPPG_06643 | SzRabexB | Vps9 | Unknown |
| VarpL | T. trahens | AMSG_07173 | TtVarpL | Vps9 | Varp |
| Rabex5 | T. trahens | AMSG_02562 | TtRabexA | Vps9 | Rabex+GAPVD1 |
| Rabex5 | T. trahens | AMSG_10386 | TtRabexB | Vps9 | Rabex+GAPVD1 |
| Rabex5 | T. trahens | AMSG_00807 | TtRabexC | Vps9, peptidoglycan-binding domain | Unknown |
| Vps9 DCP | T. trahens | AMSG_08742 | TtV9DCP | Vps9 | Unknown |
| Unknown | T. trahens | AMSG_04366 | TtUnkn1 | syntaxin-like domain, MORN, Vps9 | Unknown |
| Unknown | T. trahens | AMSG_04707 | TtUnkn2 | Vps9 | Unknown |
| Unknown | T. trahens | AMSG_11767 | TtUnkn3 | Rab GTPase domain, Vps9 | Unknown |
| Unknown | T. trahens | AMSG_05365 | TtUnkn4 | Zn-finger domain, Vps9, bZIP, FYVE | Unknown |
| GAP/Vps9 | T. trahens | AMSG_04382 | TtGAPVD1 | RasGAP, Vps9 | Unknown |

**Not in Fungi+metazoa tree: removed as a long branch (Supplementary Figure 1C)

**Amoebozoa**

| **Preliminary annotation** | **Organism** | **Accession** | **Tree name** | **Domain structure (CDD)** | **Actual identity** |
|---|---|---|---|---|---|
| VarpL | D. discoideum | DDB0348261 | DdVarpL1 | Vps9 | Varp |
| Rabex5 | D. discoideum | DDB0233291 | DdRabexA | PX, P-loop NTPase, Vps9 | Rabex+GAPVD1 |
| GAP/Vps9 | D. discoideum | DDB0234180 | DdGAPVD1A | Vps9 | Rabex+GAPVD1 |
| Rabex5 | D. discoideum | DDB0234179 | DdRabexB | Vps9 | Rabex+GAPVD1 |
| VarpL | D. discoideum | DDB0305990 | DdVarpL2 | Ank, Ank, Vps9 | Unknown |
| Unknown | D. discoideum | DDB0234182 | DdUnkn1 | Vps9, alpha/beta-hydrolase family | Rabex+GAPVD1 |
| Unknown | D. discoideum | DDB0306965 | DdV9DCP1 | Vps9 | Unknown |

| | | | | | |
|---|---|---|---|---|---|
| Unknown | D. discoideum | DDB0234181 | DdRabexC | Vps9 | Rabex+GAPVD1 |
| Unknown | D. discoideum | DDB0233768 | DdGAPVD1B | RasGAP, Vps9 | GAPVD1 |
| Unknown | D. discoideum | DDB0234183 | DdRIN | Vps9 | potential RIN |
| Unknown | D. discoideum | DDB0234184 | DdUnkn2 | Vps9, alpha/beta-hydrolase family | Rabex+GAPVD1 |
| Unknown | D. discoideum | DDB0307572 | DdUnkn3 | Vps9 | Unknown |
| GAP/9 | Filamoeba nolandi | CAMPEP_0168576556 | Filno1 | Vps9 | Rabex+GAPVD1 |
| Vps9 | Filamoeba nolandi | CAMPEP_0168550092 | Filno2 | Vps9 | Rabex+GAPVD1 |
| Rabex5 | Filamoeba nolandi | CAMPEP_0168540520 | Filno3 | Vps9 | Potential Rabex+GAPVD1 |
| Unkn | Filamoeba nolandi | CAMPEP_0168569582 | Filno4 | Vps9, PH-like, FERM-B, PH-like | Unknown |
| RIN | Filamoeba nolandi | CAMPEP_0168546012 | Filno5 | Vps9 | Unknown |
| V9DCP | Filamoeba nolandi | CAMPEP_0168542822 | Filno6 | Vps9 | Unknown |
| V9DCP | Filamoeba nolandi | CAMPEP_0168540146 | Filno7 | Vps9 | Vps9 |
| Varp | Filamoeba nolandi | CAMPEP_0168567090 | Filno8 | Vps9 | Varp |
| Varp | Filamoeba nolandi | CAMPEP_0168556614 | Filno9 | Vps9, Ank, LysM | Rabex+GAPVD1 |
| Alsin | Filamoeba nolandi | CAMPEP_0168540604 | Filno10 | MORN, Vps9 | Unknown |
| V9DCP | Filamoeba nolandi | CAMPEP_0168540338 | Filno11 | Vps9 | Unknown |
| Unknown | Filamoeba nolandi | CAMPEP_0168554466 | Filno12 | SH2, CUE/Ub, Vps9, chitin synth., TPR | Vps9 |
| Alsin | Filamoeba nolandi | CAMPEP_0168545716 | Filno13 | PH, MORN, Vps9 | Unknown |
| Vps9 | Sexangularia sp. | CAMPEP_0170733176 | Sex1 | Vps9, ubiquitin-like | Rabex+GAPVD1 |
| Unknown | Sexangularia sp. | CAMPEP_0170739412 | Sex2 | MORN, SH3, Vps9 | Potential Alsin |
| Unknown | Sexangularia sp. | CAMPEP_0170749886 | Sex3 | Vps9 | Potential Alsin |
| Vps9 | Sexangularia sp. | CAMPEP_0170757612 | Sex4 | Vps9, RhoGEF | Potential Alsin |
| Unknown | Sexangularia sp. | CAMPEP_0170755466 | Sex5 | Vps9 | Potential Alsin |
| Unknown | Sexangularia sp. | CAMPEP_0170731982 | Sex6 | Vps9, SH2 | Potential Alsin |
| Unknown | Sexangularia sp. | CAMPEP_0170730656 | Sex7 | Vps9, SH2 | Unknonw |
| Unknown | Sexangularia sp. | CAMPEP_0170733518 | Sex8 | Vps9 | Potential Alsin |
| Unknown | Sexangularia sp. | CAMPEP_0170746722 | Sex9 | Vps9 | Rabex+GAPVD1 |
| Unknown | Mayorella sp. | CAMPEP_0174231770 | Mayo1 | Vps9 | Rabex+GAPVD1 |
| Unknown | Mayorella sp. | CAMPEP_0174246948 | Mayo2 | Rilp domain, Vps9 | Vps9 |
| Unknown | Mayorella sp. | CAMPEP_0174231768 | Mayo3 | Vps9 | Unknown |
| Alsin? | Mayorella sp. | CAMPEP_0174231688 | Mayo4 | Vps9, RCC | Unknown |
| Unknown | Mayorella sp. | CAMPEP_0174248598 | Mayo5 | Vps9 | Potential Alsin |
| Unknown | Mayorella sp. | CAMPEP_0174233898 | Mayo6 | MORN, MORN, Vps9 | Potential Alsin |

| | | | | | |
|---|---|---|---|---|---|
| Unknown | Mayorella sp. | CAMPEP_0174229322 | Mayo7 | Vps9 | Unknown |
| Unknown | Mayorella sp. | CAMPEP_0174232008 | Mayo8 | P-loop NTPase, Vps9 | Alsin |
| Unknown | Trichosphaerium sp. | CAMPEP_0168524954 | Tri1 | Vps9 | Rabex+GAPVD1 |
| Unknown | Trichosphaerium sp. | CAMPEP_0168511098 | Tri2 | RhoGEF, Vps9 | Vps9 |
| Unknown | Trichosphaerium sp. | CAMPEP_0168517238 | Tri3 | Vps9 | Vps9 |
| Unknown | Trichosphaerium sp. | CAMPEP_0168513960 | Tri4 | Vps9 | GAPVD1 |
| VarpL | Trichosphaerium sp. | CAMPEP_0168514830 | Tri5 | Vps9 | Varp |
| Unknown | Trichosphaerium sp. | CAMPEP_0168523160 | Tri6 | Vps9 | Vps9 |
| Unknown | Trichosphaerium sp. | CAMPEP_0168516324 | Tri7 | DEP, Vps9 | Vps9 |
| Unknown | Trichosphaerium sp. | CAMPEP_0168522850 | Tri8 | Vps9 | Alsin |
| Alsin? | Trichosphaerium sp. | CAMPEP_0168514184 | Tri9 | Vps9 | Alsin |
| Unknown | Pessonella sp. | CAMPEP_0168595804 | Pes1 | Vps9 | Rabex+GAPVD1 |
| Unknown | Pessonella sp. | CAMPEP_0168597104 | Pes2 | Vps9 | Vps9 |
| Unknown | Pessonella sp. | CAMPEP_0168587842 | Pes3 | Vps9 | Unknown |
| Unknown | Pessonella sp. | CAMPEP_0168584954 | Pes4 | PH, Vps9 | Rabex+GAPVD1 |
| Unknown | Pessonella sp. | CAMPEP_0168591660 | Pes5 | Vps9 | Unknown |
| Unknown | Pessonella sp. | CAMPEP_0168582688 | Pes6 | MORN, Vps9 | Vps9 |
| Unknown | Pessonella sp. | CAMPEP_0168600460 | Pes7 | Vps9 | Rabex |
| Unknown | Vannella sp. | CAMPEP_0114604624 | Van1 | P-loop ATPase, Vps9 | Unknown |
| Alsin? | Vannella sp. | CAMPEP_0114614540 | Van2 | MORN, MORN, Vps9 | Vps9 |
| Unknown | Vannella sp. | CAMPEP_0114618492 | Van3 | Vps9 | Unknown |
| Unknown | Vannella sp. | CAMPEP_0114610138 | Van4 | Kelch, Vps9 | Vps9 |
| Unknown | Vannella sp. | CAMPEP_0114612628 | Van5 | Vps9 | Vps9 |
| Unknown | Vannella sp. | CAMPEP_0114608820 | Van6 | Vps9 | Vps9 |
| Unknown | Vannella sp. | CAMPEP_0114628764 | Van7 | TPR, Vps9 | Rabex+GAPVD1 |
| Unknown | Vannella sp. | CAMPEP_0114609996 | Van8 | Vps9 | Rabex+GAPVD1 |
| Rabex5? | Vannella sp. | CAMPEP_0114608898 | Van9 | Vps9, PH | Rabex+GAPVD1 |
| Unknown | Vannella sp. | CAMPEP_0114607094 | Van10 | CRP, PH, Vps9, Sh3, CRP | Rabex+GAPVD1 |
| Unknown | Vannella sp. | CAMPEP_0114619210 | Van11 | Vps9 | Rabex+GAPVD1 |
| Rabex5? | Vannella sp. | CAMPEP_0114619588 | Van12 | Vps9 | unclear |
| Unknown | Paramoeba atlantica | CAMPEP_0201478894 | Parat1 | Vps9 | Rabex+GAPVD1 |
| Unknown | Paramoeba atlantica | CAMPEP_0201500068 | Parat2 | Vps9 | Unknown |
| Unknown | Paramoeba atlantica | CAMPEP_0201483932 | Parat3 | Vps9 | Unknown |

| | | | | | |
|---|---|---|---|---|---|
| Unknown | Paramoeba atlantica | CAMPEP_0201502036 | Parat4 | Zn-finger, Vps9 | Unknown |
| Unknown | Vexillifera sp. | CAMPEP_0201563168 | Vex1 | Vps9 | Unknown |
| Unknown | Vexillifera sp. | CAMPEP_0201559588 | Vex2 | Vps9 | Unknown |
| Alsin? | Vexillifera sp. | CAMPEP_0201549744 | Vex3 | Vps9 | Unknown |
| Unknown | Vexillifera sp. | CAMPEP_0201561904 | Vex4 | Vps9 | Unknown |
| Unknown | Neoparamoeba aestuarina | CAMPEP_0201515200 | Neoae1 | Vps9 | Rabex+GAPVD1 |
| Unknown | Neoparamoeba aestuarina | CAMPEP_0201529844 | Neoae2 | Vps9 | Unknown |
| Unknown | Neoparamoeba aestuarina | CAMPEP_0201519786 | Neoae3 | SAP, Vps9 | Unknown |
| Unknown | Neoparamoeba aestuarina | CAMPEP_0201514824 | Neoae4 | Vps9 | Unknown |
| Unknown | Neoparamoeba aestuarina | CAMPEP_0201540294 | Neoae5 | Vps9 | Unknown |

**Excavata**

| **Preliminary annotation** | **Organism** | **Accession** | **Tree name** | **Domain structure (CDD)** | **Actual identity** |
|---|---|---|---|---|---|
| Rabex5 | T. cruzi | Tbg972.10.12220 | TcRabexA | Vps9 | Rabex+GAPVD1 |
| Rabex5 | T. cruzi | Tbg972.3.2410 | TcRabexB | MORN, Vps9 | Alsin |
| Rabex5 | T. brucei | Tb927.10.10020 | TbRabexA | Vps9 | Rabex+GAPVD1 |
| Rabex5 | T. brucei | Tb927.3.2430 | TbRabexB | MORN, Vps9 | Alsin |
| GAP/Vps9 | B. saltans | BS52770 | BsGAPVD1 | Vps9 | Unknown |
| Rabex5 | B. saltans | BS29355 | BsRabex | NLBH, Vps9 | Unknown |
| RIN3 | B. saltans | BS53520 | BsRIN3 | Vps9 | potential RIN |
| Vps9 DCP | B. saltans | BS00290 | BsV9DCP | Vps9 | Unknown |
| Unkn | B. saltans | BS79490 | BsUnkn | MORN, Vps9 | Alsin |
| Alsin | N. gruberi | 57768 | NgAlsinA | Vps9 | Varp |
| Rabex5 | N. gruberi | 51184 | NgRabexA | Vps9 | Unknown |
| Vps9 DCP | N. gruberi | 80473 | NgV9DCPA | PH, Rossman fold, Vps9, DUF547 | Unknown |
| Rabex5 | N. gruberi | 80732 | NgRabexB | TMPIT superfamily, Vps9 | Unknown |
| Alsin | N. gruberi | 79768 | NgAlsinB | MORN, MORN, Vps9 | Unknown |
| GAP/Vps9 | N. gruberi | 70809 | NgGAPVD1 | RasGAP, Vps9 | potential GAPVD1 |
| Vps9 DCP | N. gruberi | 79743 | NgV9DCPB | Vps9 | Unknown |
| RIN2 | N. gruberi | 69961 | NgRIN2 | Fam196 superfamily, Cgr1 family, Vps9 | Unknown |
| Unkn | N. gruberi | 66586 | NgUnkn | Vps9 | Unknown |
| Alsin | N. gruberi | 77717 | NgAlsinC | MORN, MORN; no clear Vps9 domain | Unknown |

**Archaeplastida**

| Preliminary annotation | Organism | Accession | Tree name | Domain structure (CDD) | Actual identity |
|---|---|---|---|---|---|
| Rabex5 | O. tauri | 36941 | OtRabex | Vps9 | Rabex+GAPVD1 clade |
| Vps9 DCP | O. tauri | 36050 | OtV9DCP | Vps9 | Varp |
| GAP/Vps9 | M. pusilla | 34555 | MpGAPVD1 | Vps9 | Rabex+GAPVD1 clade |
| GAP/Vps9 | C. reinhardtii | 173295 | CrGAPVD1 | Vps9 | Rabex+GAPVD1 clade |
| VarpL | S. moellendorffii | 444026 | SmVarpL | Vps9 | Varp |
| GAP/Vps9 | S. moellendorffii | 81029 | SmGAPVD1 | Vps9 | Rabex+GAPVD1 clade |
| GAP/Vps9 | A. thaliana | AT3G19770.1 | AtGAPVD1A | Vps9 | Rabex+GAPVD1 clade |
| GAP/Vps9 | A. thaliana | AT5G09320.1 | AtGAPVD1B | Vps9, PPR repeat | Rabex+GAPVD1 clade |
| GAP/Vps9 | C. paradoxa | ** | CpGAPVD1 | Vps9 | Rabex+GAPVD1 clade |
| VarpL | G. theta | 102431 | GtVarpL | Vps9 | Varp |
| Rabex5 | G. theta | 152908 | GtRabexA | Vps9 | Rabex+GAPVD1 clade |
| Rabex5 | G. theta | 40600 | GtRabexB | Vps9 | Rabex+GAPVD1 clade |
| Rabex5 | G. theta | 151319 | GtRabexC | Vps9 | Rabex+GAPVD1 clade |
| Rabex5 | G. theta | 101593 | GtRabexD | Vps9, M domain of GW182 | Rabex+GAPVD1 clade |

** Contig25900-snap-gene-0.0-mRNA-1:cds:5900/4323-4503:0:+

**Alveolata**

| Preliminary annotation | Organism | Accession | Tree name | Domain structure (CDD) | Actual identity |
|---|---|---|---|---|---|
| VarpL | P. marinus | XP_002780998 | PmVarpL | Vps9 | Unknown |
| Rabex5 | T. gondii | TGGT1_117760 | TgRabex | Vps9 | Rabex-GAPVD1 clade |
| Unknown | T. gondii | TGGT1_005790 | TgUnkn | Vps9 | Varp |
| Vps9/HSP70 | T. thermophila | 159.m00052 | TetV9H70 | Vps9, Hsp70 | Rabex-GAPVD1 clade |
| Alsin | T. thermophila | 3717.m00021 | TetAlsn1 | Vps9 | Unknown |
| Rabex5 | T. thermophila | 1.m00854 | TetRabxA | RasGAP, Vps9 | Rabex-GAPVD1 clade |
| Vps9 DCP | T. thermophila | 232.m00051 | TetV9DP1 | Vps9 | Unknown |
| Vps9 DCP | T. thermophila | 3738.m00037 | TetV9DP2 | Vps9 | Unknown |
| Alsin | T. thermophila | 17.m00275 | TetAlsn2 | Vps9 | Unknown |
| Rabex5 | T. thermophila | 3824.m03090 | TetRabxB | RasGAP, Vps9 | Rabex-GAPVD1 clade |
| GAP/Vps9 | T. thermophila | 119.m00124 | TetGAPVD1 | Vps9 | Rabex-GAPVD1 clade |
| Unknown | T. thermophila | 3818.m00655 | TetUnkn1 | Vps9 | Unknown |

| | | | | | |
|---|---|---|---|---|---|
| RIN1 | T. thermophila | 315.m00019 | TetRIN1 | Vps9 | Unknown |
| Rabex5 | T. thermophila | 16.m00334 | TetRabxC | Vps9 | Unknown |
| Unknown | T. thermophila | 3728.m00081 | TetUnkn2 | Vps9 | Unknown |

**Stramenopiles**

| **Preliminary annotation** | **Organism** | **Accession** | **Tree name** | **Domain structure (CDD)** | **Actual identity** |
|---|---|---|---|---|---|
| VarpL | A. anophagefferens | 72474 | AaVarpL1 | Vps9 | Varp |
| Rabex5 | A. anophagefferens | 17718 | AaRabexA | Vps9 | Rabex-GAPVD1 |
| Rabex5 | A. anophagefferens | 71564 | AaRabexB | Vps9 | TEA-specific |
| RIN3 | A. anophagefferens | 70742 | AaRIN | PDZ, PX, Vps9 | Stramenopile- or TEA-specific sequence |
| VarpL | A. anophagefferens | 70964 | AaVarpL2 | Vps9, Ank, Ank | Rabex-GAPVD1 |
| Rabex5 | E. siliculosus | Esi0321_0027 | EsRabexA | Vps9 | Rabex-GAPVD1 |
| Varp | E. siliculosus | Esi0135_0039 | EsVarp | DUF47, Vps9, Ank, Ank | Rabex-GAPVD1 |
| Alsin | E. siliculosus | Esi0008_0117 | EsAlsin | PDZ, PX, Vps9 | Stramenopile- or TEA-specific sequence |
| Rabex5 | E. siliculosus | Esi0227_0037 | EsRabexB | Vps9, PX, Vps9 | Unknown |
| Rabex5 | E. siliculosus | Esi0306_0023 | EsRabexC | Vps9 | Rabex-GAPVD1 |
| Unknown | E. siliculosus | Esi0314_0013 | EsUnkn1 | PDZ, Vps9 | Stramenopile- or TEA-specific sequence |
| Unknown | E. siliculosus | Esi0018_0145 | EsUnkn2 | C1, DUF456, Vps9 | TEA-specific |
| Rabex5 | T. pseudonana | 22869 | TpRabexC | RRP14, SURF6, B-propeller, Rad1, Vps9 | Rabex-GAPVD1 |
| Rabex5 | T. pseudonana | 11750 | TpRabexA | PDZ, PX, Vps9 | Stramenopile- or TEA-specific sequence |
| Rabex5 | T. pseudonana | 22297 | TpRabexB | Vps9 | TEA-specific |
| Rabex5 | P. sojae | 138309 | PsRabexA | RmuC family protein, Vps9 | Rabex-GAPVD1 |
| RIN2 | P. sojae | 142782 | PsRIN2 | Vps9 | Unknown |
| GAP/Vps9 | P. sojae | 141276 | PsGAPVD1A | Vps9 | Unknown |
| Rabex5 | P. sojae | 138892 | PsRabexB | Vps9 | Rabex-GAPVD1 |
| VarpL | P. sojae | 128587 | PsVarpL | PH, Vps9, Ank, RasGEF | Unknown |
| Vps9 DCP | P. sojae | 158240 | PsV9DCP | PX, Vps9 | Unknown |
| GAP/Vps9 | P. sojae | 131887 | PsGAPVD1B | Vps9 | Unknown |
| GAP/Vps9 | P. sojae | 142773 | PsGAPVD1C | GATase1-like domain, Vps9 | Unknown |
| RINL | P. sojae | 136726 | PsRINL | Ub/CUE domain, Vps9 | Unknown |
| Unknown | P. sojae | 138708 | PsUnkn1 | PH, PH, Vps9, PDZ | Unknown |
| RIN3 | P. sojae | 136111 | PsRIN3 | PH, Vps9 | Unknown |
| Unknown | P. sojae | 138321 | PsUnkn2 | Vps9 | potential Vps9 |

| | | | | | |
|---|---|---|---|---|---|
| Alsin | A. limacinum | 132195 | AlAlsin | SH3, Vps9, RCC1 | Varp |
| Rabex5 | A. limacinum | 100340 | AlRabexA | Vps9 | Rabex-GAPVD1 |
| Rabex5 | A. limacinum | 139971 | AlRabexB | Vps9 | Rabex-GAPVD1 |
| RIN2 | A. limacinum | 136168 | AlRIN2 | RasGEF, Vps9 | Unknown |
| Vps9 DCP | A. limacinum | 145430 | AlV9DCP | Metal-dependent hydrolase, Vps9 | Vps9 |
| GAP/Vps9 | A. limacinum | 42534 | AlGAPVD1 | No clear Vps9 domain | Varp |
| RIN | A. limacinum | 33039 | AlRIN3 | PX, P-loop ATPase, Vps9 | Unknown |

**Rhizaria+Haptophyta**

| Preliminary annotation | Organism | Accession | Tree name | Domain structure (CDD) | Actual identity |
|---|---|---|---|---|---|
| Alsin | E. huxleyi | 218203 | EhAlsin | Vps9 | Unclear |
| Unknown | E. huxleyi | 247265 | EhUnkn1 | Vps9 | Unclear |
| Unknown | E. huxleyi | 239650 | EhUnkn2 | Vps9 | Unclear (highly similar to EhUnkn1) |
| Unknown | E. huxleyi | 354427 | EhUnkn3 | Vps9 | Vps9 |
| VarpL | B. natans | 86030 | BnVarpL1 | Vps9 | Varp |
| Rabex5 | B. natans | 48825 | BnRabex | Vps9 | Rabex+GAPVD1 |
| RIN1 | B. natans | 80631 | BnRIN1 | Vps9 | Rhizaria-specific clade 1 |
| Vps9 DCP | B. natans | 74481 | BnV9DCP | WWE, Vps9 | Unclear |
| VarpL | B. natans | 81278 | BnVarpL2 | Vps9 | potentially Varp |
| Unknown | B. natans | 67055 | BnUnkn1 | Vps9 | potentially Varp |
| Unknown | B. natans | 69446 | BnUnkn2 | Vps9 | Unclear |
| Unknown | B. natans | 68567 | BnUnkn3 | DEP/PH, Vps9 | Unclear |
| Unknown | B. natans | 74004 | BnUnkn4 | Vps9 | Rabex+GAPVD1 |
| Unknown | B. natans | 68971 | BnUnkn5 | Vps9 | Unclear |
| Unknown | B. natans | 76472 | BnUnkn6 | Vps9 | unclear (potential Varp ) |
| Unknown | B. natans | 75886 | BnUnkn7 | Vps9 | Unclear (Same as BnUnkn1) |
| VarpL | R. filosa | ETO30791 | RfVarpL1 | Vps9 | Unknown |
| VarpL | R. filosa | ETO16813 | RfVarpL2 | No clear vps9 domain | Rhizaria-specific clade 1 |
| VarpL | R. filosa | ETO11205 | RfVarpL3 | Vps9 | Varp |
| Vps9 DCP | R. filosa | ETO22898 | RfV9DCP1 | Vps9 | Reticulomyxa-specific clade 1 |
| RIN2 | R. filosa | ETO29831 | RfRIN2 | Vps9 | Rhizaria-specific clade 1 |
| Vps9 DCP | R. filosa | ETO21800 | RfV9DCP2 | Vps9 | Reticulomyxa-specific clade 1 |
| VarpL | R. filosa | ETO16140 | RfVarpL4 | Vps9 | Reticulomyxa-specific clade 1 |

| | | | | | |
|---|---|---|---|---|---|
| RIN3 | R. filosa | ETO21187 | RfRIN3 | Vps9 | Reticulomyxa-specific clade 1 |
| Unknown | R. filosa | ETO19750 | RfUnkn1 | CAP-Gly, Vps9 | Rhizaria-specific clade 1 |
| GAP/Vps9 | R. filosa | ETO21137 | RfGAPVD1A | Vps9 | Unclear |
| Unknown | R. filosa | ETO26643 | RfUnkn2 | CAP-Gly, Vps9 | Rhizaria-specific clade 1 |
| GAP/Vps9 | R. filosa | ETO18729 | RfGAPVD1B | Vps9 | Rhizaria-specific clade 1 |
| Unknown | R. filosa | ETO19346 | RfUnkn3 | Vps9 | Reticulomyxa-specific clade 1 |
| GAP/Vps9 | R. filosa | ETO08105 | RfGAPVD1C | Vps9 | Unknown |
| Unknown | R. filosa | ETO36899 | RfUnkn4 | Vps9 | Unknown** |
| Unknown | R. filosa | ETO26884 | RfUnkn5 | Vps9 | Unknown |
| Unknown | R. filosa | ETO19808 | RfUnkn6 | Vps9 | Unknown** |
| Unknown | R. filosa | ETO13085 | RfUnkn7 | Vps9 | Rhizaria-specific clade 1 |
| Unknown | R. filosa | ETO20382 | RfUnkn8 | Vps9 | Rhizaria-specific clade 1 |
| Unknown | R. filosa | ETO28139 | RfUnkn9 | Vps9 | Unknown |

**assignment based on phylogenetic affinity to RfGAPVD1C; not in Supplementary Figure 1N

**Phylogenetics parameters**

| **Figure** | **Model** | **alpha (+G)** | **Invariant sites (+I)** |
|---|---|---|---|
| Figure 2 | LG+G+F | 2.721 | |
| Figure 3 | LG+G+F | 1.703 | |
| Supp. Figure 1A | LG+G+F | 2.513 | |
| Supp. Figure 1B | LG+I+G | 2.464 | 0 |
| Supp. Figure 1C | LG+G | 1.918 | |
| Supp. Figure 1D | LG+G+F | 1.922 | |
| Supp. Figure 1E | LG+G+F | 1.726 | |
| Supp. Figure 1F | LG+I+G+F | 1.935 | 0.008 |
| Supp. Figure 1G | LG+G+F | 1.992 | |
| Supp. Figure 1H | LG+I+G+F | 1.93 | 0.009 |
| Supp. Figure 1I | LG+G+F | 1.965 | |
| Supp. Figure 1J | LG+G+F | 2.108 | |
| Supp. Figure 1K | LG+G+F | 2.375 | |
| Supp. Figure 1L | LG+G+F | 1.805 | |
| Supp. Figure 1M | LG+G+F | 2.183 | |

| | | | |
|---|---|---|---|
| Supp. Figure 1N | LG+G+F | 2.343 | |
| Supp. Figure 1O | LG+G+F | 1.845 | |
| Supp. Figure 1P | LG+I+G+F | 1.874 | 0.009 |
| Supp. Figure 1Q | LG+G+F | 2.166 | |
| Supp. Figure 1R | LG+G | 1.489 | |
| Supp. Figure 1S | LG+G+F | 1 | |
