# Supplementary material for: Regulation of early endosomes across eukaryotes: Evolution and functional homology of Vps9 proteins
Source: Traffic. 2018 Apr 25;19(7):546–63. doi: 10.1111/tra.12570 (PMC6032885; doi:10.1111/tra.12570)
Supplement: Supplementary file 1 — Editorial Process [file TRA-19-546-s002.pdf]

Title:	**Regulation of early endosomes across eukaryotes: Evolution and functional homology of Vps9 proteins**
Authors:	Emily K. Herman, Moazzam Ali, Mark C. Field, Joel B. Dacks
Article Type:	Original Research

Monitoring Editor	Christian Ungermann
Date Submitted	10 January 2018
Date for Decision 1	21 January 2018
Date Resubmitted	2 March 2018
Date for Decision 2	6 March 2018
Accepted	27 March 2018

Decision and Reviews

Dear Dr. Herman and Dr. Field,

Thank you for submitting your manuscript "Regulation of endocytosis across eukaryotes: Evolution and functional homology of Vps9 proteins" for consideration for publication in Traffic. I asked two colleagues who are experts in the field to review the paper and their verbatim comments are appended below. I share the view of the referees that the work presented in this paper will be of interest to the readers of Traffic. However, before the paper can be accepted, the referees have raised a number of concerns that you will need to address. While many can be addressed by revision of the text, some concerns may require that you include some controls in your experiments like additional organelle markers. The referees have very clearly outlined their concerns so I will not reiterate these here.

Although I cannot accept your manuscript for publication at this point, I believe that you will be able to address the referees' concerns and I look forward to receiving your revised manuscript. To expedite handling when you resubmit please be sure to include a response outlining how you have addressed each of the referees' concerns.

Sincerely,

Christian Ungermann, Ph.D.
Associate Editor

Referee's Comments to the Authors

Referee: 1

Comments to the Author
The Dacks and Field groups provide a thoughtful analysis of the Vps9 domain family of Rab5 exchange factors and their functions. Despite the rather general title, the work is focussed on regulation of Rab5 rather than of endocytosis and the functional data is restricted to T. brucei. This brings me to my first general suggestion, that the authors consider a more focussed title for their work. My second general suggestion concerns the length of the manuscript. While the text flows well, there are too many main figures. Figure 2 would be better as supplemental material, and some of the later figures on the functional redundancy between TbAlsin/Rabex could easily be simplified and combined. What the manuscript leaves unresolved is the precise relationship between the different TbRab5 proteins (5A/5B/21) and TbAlsin/Rabex. This is due to incomplete data in key figures. Figure 8 shows the interaction of TbAlsin with Rab5A, but does not investigate TbRabex. Do the Rabs 5B/21 that were negative for TbAlsin give a positive signal with Rabex? Ideally, they need to perform exchange factor assays for the different Rab5 family members and Vps9 domain proteins, but I assume this is not possible due to insufficient amounts of the proteins.

Other points:
It would be helpful if the authors provided a schematic in Figure 1 showing the domain organisation of the different Vps9-domain protein subfamilies. This will help a general readership.

Gray-shading of some figures makes them harder to read. I'd recommend converting these to simple black on white. If particular branches need highlighting then perhaps this could be done using coloured text as in Figure 1.

Some organisms listed in Figure 1 have very large numbers of unclassified Vps9 domain proteins. The authors might want to comment on this in the main text.

Page 8, line 26. "Alsin has a C-terminal Vps9 domain which mediates its interaction with Rab5". This text is a little odd, since the current view is that Vps9 domains promote nucleotide exchange rather than serving as interaction sites.

Page 8. Define SAR (Stramenopiles / Alveolata / Rhizaria). SAR first appears in a long subsection heading on Page 8. Ideally, the text should be rewritten to avoid this.

Page 10, lines 5-7. This sentence doesn't entirely make sense; there is either a typo or a word missing. "Rabex5 (or Vps9p in yeast) acts downstream, and in complex with the Rab5 effector Rabaptin-5, is essential for homotypic endosome fusion (Horiuchi et al., 1997)".

Microscope images lack specific organelle markers. This is important since the text (see page 13, line 10-12) notes that the region stained contains Golgi as well as endosomes.

Figure 4C. The image for TbAlsin is not of the same clarity as those shown for TbRabex, and makes it difficult to comment on localisation. Can the authors provide a clearer example?

Figure 8 shows a characterisation of the interaction of TbAlsin with Rab5 mutants. The authors find that Rab5 binds to a nucleotide-free SN mutant, as expected for an exchange factor. This is referred to as a GDP-bound mutant in the text, and this should be corrected.

Referee: 2

Comments to the Author
This interesting manuscript describes whole genome phylogenetic analyses of Vps9 domain proteins from many diverse organisms, allowing reconstruction of broad evolutionary relationships across eukaryotes dating back to the last eukaryotic common ancestor. Vps9 domain proteins have been investigated mainly in animals and fungi, where they function as GEFs for Rab5, Rab21 and possibly other Rabs. The manuscript also provides the first characterization of the localization and function of the Vps9 domains proteins from an organism beyond opisthokonts. The evolutionary analyses and functional experiments appear to be well executed. Overall, the manuscript is well written and represents a valuable contribution to the filed, including a broad overview on the evolution of the Vps9 family as well as a rather unique perspective on the functional role of the two Vps9 proteins in a trypanosome.

Specific comments:

1) Following the zinc finger, Rabex-5 has a second ubiquitin binding domain (dubbed the MIU since it appears to be an inverted UIM; Penengo et al. 2006 Cell 124 1183-1195; Lee et al. 2006 NSMB 13 264-271). Also, the coiled coil after the Vps9 domain contributes to endosome recruitment through interaction with Rabaptin-5 (Mattera et al. 2008). Is it possible to determine whether the MIU and/or coiled were acquired at the same stage in the evolutionary lineage as the zinc finger or perhaps not?

2) From the description of the western blots on p. 14-15, it is not entirely clear if the knockdown efficiency is the same for the single vs. double knockdowns. Is there a difference in protein levels for one or both proteins in the single vs. double knockdowns? If so, could the stronger phenotype in the double knockdown be a consequence of lower protein levels rather than functional overlap? There are many examples of synthetic effects for genes that function in distinct pathways.

Minor:

3) P. 3, lines 25-26. "GTP loading" is somewhat ambiguous (e.g. does it mean exchange, GTP association, etc). It

would be more precise to say that GEFs accelerate exchange of GDP for GTP.

4) P. 4, lines 2-4. The structural details of Rab21 and a plant Rab5 bound to Vps9 domains have been described:
Delprato et al. 2007 Nat Struct Mol Biol 14 406-412.
Uejima et al. 2010 J Biol Chem 285 36689-36697.

5) P. 4, line 12. Define MTS.

6) P. 6, line 7. What is a MrBayes supergroup? Probably won't be obvious to most readers.

7) P. 6, line 15. What is meant by deep? An early duplication or something else?

Author Rebuttal

Dear Editor,

I hereby submit the revised version of our manuscript TRA-18-0709, entitled "Regulation of early endosomes across eukaryotes: Evolution and functional homology of Vps9 proteins", on behalf of all co-authors and myself. We have addressed each of the reviewers' suggestions, as detailed below. I hope that the improved manuscript is now suitable for publication.

Best wishes,
Emily Herman

Referee's Comments to the Authors

Referee: 1

Comments to the Author
The Dacks and Field groups provide a thoughtful analysis of the Vps9 domain family of Rab5 exchange factors and their functions. Despite the rather general title, the work is focussed on regulation of Rab5 rather than of endocytosis and the functional data is restricted to T. brucei. This brings me to my first general suggestion, that the authors consider a more focussed title for their work.

We have modified the title to indicate that the focus of the article is on early endosome regulation, rather than endocytosis in general. The new title is "Regulation of early endosomes across eukaryotes: Evolution and functional homology of Vps9 proteins."

My second general suggestion concerns the length of the manuscript. While the text flows well, there are too many main figures. Figure 2 would be better as supplemental material, and some of the later figures on the functional redundancy between TbAlsin/Rabex could easily be simplified and combined.

As Figure 2 displays the major conclusion from the comparative genomic portion of the manuscript – that there were at least three Vps9 domain-containing proteins present in the LECA – we do not feel that it belongs in the Supplementary information. As for the latter statement, we are not sure how the reviewer suggests simplifying or combining figures involving TbRabex and TbAlsin. The current figures have 3-5 panels, making it difficult to merge them yet properly display individual images. Also, although our work suggests that TbAlsin and TbRabex have partly redundant functions (and hence why we focus on the effect of the double-knockdown on endocytosis), the two Vps9 proteins vary in their localization and ability to interact with Rab5 family proteins, which we feel are necessary results to show. However, if the reviewer feels strongly about the number of figures and has specific suggestions regarding how to rework them, we are willing to consider those changes.

What the manuscript leaves unresolved is the precise relationship between the different TbRab5 proteins (5A/5B/21) and TbAlsin/Rabex. This is due to incomplete data in key figures. Figure 8 shows the interaction of TbAlsin with Rab5A, but does not investigate TbRabex.

In this figure we examine both the interaction of Rabs with TbAlsin and TbRabex (panels B and C). We also provide a control pullout in panel D. From these data we conclude that the only evidence for interaction is with TbAlsin and Rab5A - a faint band is seen in the TbRabex pullouts, but here the intensely is equal to that from the negative control,

leading us to conclude that, under these conditions, we are unable to coIP a Rab/Rabex complex. We apologise if this is unclear - these sorts of figures are quite complex.

Do the Rabs 5B/21 that were negative for TbAlsin give a positive signal with Rabex? Ideally, they need to perform exchange factor assays for the different Rab5 family members and Vps9 domain proteins, but I assume this is not possible due to insufficient amounts of the proteins.

These data are shown in Figure 8, and we did not find evidence. The reviewer is correct that production of sufficient material for exchange assays does require significant amounts of material, and in this experiment a simple in vitro translation assay was used to generate protein, so indeed we do not have 'biochemical' quantities available.

Other points:
It would be helpful if the authors provided a schematic in Figure 1 showing the domain organisation of the different Vps9-domain protein subfamilies. This will help a general readership.

While we agree that a diagram of the domain organisation of Vps9 domain-containing proteins would be helpful for readers, the numbers of main and supplementary figures in the manuscript are the maximum allowed by Traffic. If the reviewer feels strongly that this would be beneficial for the manuscript, we would gladly include a diagram as part of Supplementary Figure 1.

Gray-shading of some figures makes them harder to read. I'd recommend converting these to simple black on white. If particular branches need highlighting then perhaps this could be done using coloured text as in Figure 1.

We have removed gray shading from Figure 3, and have lightened the shading in the trees in Supplementary Figure 1. We find that denoting clades only by boxes makes them too 'busy' (particularly the Rabex+GAPVD1, Rabex, and GAPVD1 clades) and difficult to interpret. If the trees in Supplementary Figure 1 are still hard to read, we will remove the gray shading altogether. However, we feel that the lighter gray colour has improved readability.

Some organisms listed in Figure 1 have very large numbers of unclassified Vps9 domain proteins. The authors might want to comment on this in the main text.

The reviewer raises an interesting point worth exploring; we have added the following text to the Results section (page 6 lines 2-9):
"While many Vps9 domain-containing sequences across eukaryotes could be classified as orthologous to those characterized in human and yeast, others could not be classified in this way (Figure 1). In some cases, these sequences are clade-specific expansions that have not yet been functionally characterized due to their absence from typical model organisms (e.g. a clade of Stramenopile-specific Vps9 domain-containing proteins, Supplementary Table 1). In other cases, the failure to classify these sequences may be due to high levels of sequence divergence, raising the possibility of neofunctionalisation."

Page 8, line 26. "Alsin has a C-terminal Vps9 domain which mediates its interaction with Rab5". This text is a little odd, since the current view is that Vps9 domains promote nucleotide exchange rather than serving as interaction sites.

We have clarified this in the text (page 9 line 6) "Alsin has a C-terminal Vps9 domain which promotes the dissociation of GDP from Rab5..."

Page 8. Define SAR (Stramenopiles / Alveolata / Rhizaria). SAR first appears in a long subsection heading on Page 8. Ideally, the text should be rewritten to avoid this.

We have now spelled out 'SAR' in the heading (page 9 line 3).

Page 10, lines 5-7. This sentence doesn't entirely make sense; there is either a typo or a word missing. "Rabex5 (or Vps9p in yeast) acts downstream, and in complex with the Rab5 effector Rabaptin-5, is essential for homotypic endosome fusion (Horiuchi et al., 1997)".

We have changed this sentence (page 10 lines 17-19): "Rabex5 (or Vps9p in yeast) acts downstream, and in complex with the Rab5 effector Rabaptin-5, it is essential for homotypic endosome fusion (Horiuchi et al., 1997)."

Microscope images lack specific organelle markers. This is important since the text (see page 13, line 10-12) notes that the region stained contains Golgi as well as endosomes.

The point about this figure is the close proximity to endosomes, and specifically Rab5A, and hence supporting evidence for an interaction between the Vps9 proteins and the Rab. It will be very difficult for us to provide additional markers (a lab move and student graduating), and we also submit that this is a considerable amount of work, which does not add to the point that we are trying to make - we do not wish to suggest that ALL Vps9 is present at an endosome, although the overlap with Rab5A is highly suggestive of this. Further, the Golgi complex is significantly closer to the nucleus that the endosomes, and we do not observe localisation at that region.

Figure 4C. The image for TbAlsin is not of the same clarity as those shown for TbRabex, and makes it difficult to comment on localisation. Can the authors provide a clearer example?

The image is representative, and we do not have 'better'. TbAlsin is a soluble protein, and as expected, is quite diffuse, with only a small number of puncta visible - likely points of interaction at an organelle. We do consider that the image is fully reflecting the location where TbAlsin is present. We have increased the contrast and gating in a new version of the figure, which we hope clarifies this sufficiently.

Figure 8 shows a characterisation of the interaction of TbAlsin with Rab5 mutants. The authors find that Rab5 binds to a nucleotide-free SN mutant, as expected for an exchange factor. This is referred to as a GDP-bound mutant in the text, and this should be corrected.

The SN mutant is thought to be GDP-locked (Nuoffer et al. 1994, Lee et al. 2009). We did not test nucleotide-free Rab mutants in these experiments.

Referee: 2

Comments to the Author
This interesting manuscript describes whole genome phylogenetic analyses of Vps9 domain proteins from many diverse organisms, allowing reconstruction of broad evolutionary relationships across eukaryotes dating back to the last eukaryotic common ancestor. Vps9 domain proteins have been investigated mainly in animals and fungi, where they function as GEFs for Rab5, Rab21 and possibly other Rabs. The manuscript also provides the first characterization of the localization and function of the Vps9 domains proteins from an organism beyond opisthokonts. The evolutionary analyses and functional experiments appear to be well executed. Overall, the manuscript is well written and represents a valuable contribution to the filed, including a broad overview on the evolution of the Vps9 family as well as a rather unique perspective on the functional role of the two Vps9 proteins in a trypanosome.

Specific comments:

1) Following the zinc finger, Rabex-5 has a second ubiquitin binding domain (dubbed the MIU since it appears to be an inverted UIM; Penengo et al. 2006 Cell 124 1183-1195; Lee et al. 2006 NSMB 13 264-271). Also, the coiled coil after the Vps9 domain contributes to endosome recruitment through interaction with Rabaptin-5 (Mattera et al. 2008). Is it possible to determine whether the MIU and/or coiled were acquired at the same stage in the evolutionary lineage as the zinc finger or perhaps not?

The reviewer raises an interesting question. The zinc finger appears to have been gained in the ancestor of Sphaeroforma arctica, a single-celled organism near the base of the Holozoa. Using a coiled coil prediction server (COILS), we identified a coiled coil region between 50-100 aa in animal Rabex-5 sequences including the mollusc Lottia gigantea. There are some potential coiled coils in this region in Rabex5 sequences from the anemone Nematostella vectensis and choanoflagellate Salpingoeca rosetta (both predicted with confidence values < 0.4). It would therefore appear that the coiled coil was gained at some point after the zinc finger, but was present at least in the common ancestor of coelomates. The MIU domain seems to be more recent, as the most basal animal it could be identified in confidently is the tunicate Ciona intestinalis. This is in line with the findings by Penengo and colleagues (Figure 1B in that paper). Based on these analyses, the zinc finger, coiled coil, and MIU domains were gained in Rabex-5 sequentially over the evolution of animals and their single-celled relatives. In an effort to not expand an already lengthy manuscript, we have not included these new analyses, but we hope the reviewer finds this information helpful.

2) From the description of the western blots on p. 14-15, it is not entirely clear if the knockdown efficiency is the same for the single vs. double knockdowns. Is there a difference in protein levels for one or both proteins in the single vs. double knockdowns? If so, could the stronger phenotype in the double knockdown be a consequence of lower protein levels rather than functional overlap? There are many examples of synthetic effects for genes that function in distinct pathways.

In the single knockdown, both Vps9 proteins are silenced very efficiently, with in one case no protein visible and in the second ~20%. We think it very unlikely that the residual 20% is sufficient to mask an impact that is attributable to a synthetic interaction, and in the case of the complete loss, very difficult to argue. Because of this high efficiency silencing in the single knockdowns, we are confident that the impact we see if highly unlikely to be the result of a synthetic interaction - but we have amended to text to explain this and to be open towards the very low probability that this is the case (page 15 lines 23-25).

Minor:

3) P. 3, lines 25-26. "GTP loading" is somewhat ambiguous (e.g. does it mean exchange, GTP association, etc). It would be more precise to say that GEFs accelerate exchange of GDP for GTP.

We have modified the text (page 3 lines 24-26): “The GTP cycle of Rabs is regulated by GTP-activating proteins (GAPs) and Guanine nucleotide Exchange Factors (GEFs), which accelerate the hydrolysis of GTP and exchange of GDP for GTP, respectively.”

4) P. 4, lines 2-4. The structural details of Rab21 and a plant Rab5 bound to Vps9 domains have been described:
Delprato et al. 2007 Nat Struct Mol Biol 14 406-412.
Uejima et al. 2010 J Biol Chem 285 36689-36697.

We have modified the text to include these references (page 4, lines 3-5): “Rab5 binds in a shallow hydrophobic groove between the V4 and V6 helices of the Vps9 domain in both human (Delprato & Lambright, 2007; Delprato et al., 2004) and plant (Uejima et al., 2010) structural analyses.”

5) P. 4, line 12. Define MTS.

We have defined MTS.

6) P. 6, line 7. What is a MrBayes supergroup? Probably won't be obvious to most readers.

We have clarified this sentence (page 6 lines 15-17): “The shortest-branching taxon from each subclade in each MrBAYES-generated supergroup tree was selected and carefully aligned.”

7) P. 6, line 15. What is meant by deep? An early duplication or something else?

We have clarified this sentence to indicate that the split occurred prior to the divergence to two supergroups (and is therefore relatively ancient) (page 6 lines 22-26): “The Rabex5+GAPVD1 clade splits into clear Rabex5 and GAPVD1 sequences in the Amorphea (Amoebozoa and Opisthokonta, Supplementary Figure 1A-K), suggesting an ancient, but post-LECA, gene duplication event prior to the split of the Amoebozoa and Opisthokonta supergroups.”

Decision and Reviews

Dear Dr. Herman and Dr. Field,

Thank you for submitting your revised manuscript "Regulation of early endosomes across eukaryotes: Evolution and functional homology of Vps9 proteins" to Traffic. I have read your revised paper and your response to the referees, and I am of the view that you have adequately addressed the concerns of the referees. This manuscript is now accepted for publication in Traffic.

Sincerely,

Christian Ungermann, Ph.D
Associate Editor
